# Integrating hypertension care into the existing HIV services package in Botswana (InterCARE): a pair-matched, cluster-randomised, type 2 hybrid effectiveness–implementation trial

**DOI:** 10.1016/j.langlo.2026.104041

**Published:** 2026-08-12

**Authors:** Mosepele Mosepele, Tendani Gaolathe, Kago Kebotsamang, Thato Moshomo, Moagedi Mawi, Khumo Seipone, Keonayang Kgotlaetsile, Kesaobaka Molebatsi, Onkabetse J Molefe-Baikai, Matlhogonolo Kelepile, Mareko Ramotsababa, Edwin G Mogaetsho, Ponego Ponatshego, Joseph N Jarvis, Pooja Gala, Amelia Van Pelt, Nthabiseng Phaladze, Laura Bogart, Shabbar Jaffar, Duolao Wang, Lisa R Hirschhorn

**Affiliations:** Department of Internal Medicine, University of Botswana, Gaborone, Botswana (Prof M Mosepele PhD, T Gaolathe MD, T Moshomo MMed, M Mawi BSc, K Kgotlaetsile MSc, O J Molefe-Baikai MMed, M Ramotsababa MMed, E G Mogaetsho MSc); Department of Statistics, University of Botswana, Gaborone, Botswana (K Kebotsamang MSc); African Comprehensive HIV/AIDS Partnership, Gaborone, Botswana (K Seipone MPH); Botswana Harvard Health Partnership, Gaborone, Botswana (K Molebatsi PhD, P Ponatshego MSc); Department of Environmental Science, University of Botswana, Gaborone, Botswana (M Kelepile PhD); Department of Clinical Research, London School of Hygiene & Tropical Medicine, London, UK (Prof J N Jarvis PhD); Department of Medicine, NYU Langone Grossman School of Medicine, New York, NY, USA (P Gala MPH); Department of Medical Social Sciences, Northwestern University Feinberg School of Medicine, Chicago, IL, USA (A Van Pelt PhD, Prof L R Hirschhorn MPH); School of Nursing, University of Botswana, Gaborone, Botswana (Prof N Phaladze PhD); RAND Corporation, Santa Monica, CA, USA (Prof L Bogart PhD); Institute for Global Health, University College London, London, UK (Prof S Jaffar PhD); Liver Research Center & State Key Laboratory of Digestive Health, Beijing Friendship Hospital, Capital Medical University, Beijing, China (Prof D Wang PhD); Department of Clinical Sciences, Liverpool School of Tropical Medicine, Liverpool, UK (Prof D Wang PhD)

## Abstract

**Background:**

People with HIV have poor access to care for other chronic conditions despite the increasing disease burden and associated mortality. We evaluated the impact of integrated HIV and hypertension care on blood pressure control, electronic prescribing of antihypertensive medicines, and HIV viral suppression in Botswana.

**Methods:**

We conducted a 24-month, pair-matched, cluster-randomised, type 2 hybrid effectiveness–implementation trial at 14 HIV clinics among adults aged 20–75 years with HIV and hypertension. HIV clinics were eligible if they had the Patient Integrated Medical Record System (PIMS), the national electronic health record (EHR) platform for HIV care in Botswana. Clinics were pair-matched by catchment population size, antiretroviral therapy access, age structure, and geographical location before one clinic from each pair was randomly allocated to the intervention or standard-of-care group. The main implementation strategy in the intervention group included HIV health-care provider training, ongoing coaching, engagement of community treatment partners, and the use of the EHR to support diagnosis, management, and electronic prescribing during routine HIV clinic visits. The coprimary outcomes, at 12 months, were (1) the proportion of participants taking antihypertensive medicines with blood pressure controlled within the targets (systolic and diastolic blood pressure <140 mm Hg and <90 mm Hg, respectively; or <130 mm Hg and <80 mm Hg for those with diabetes or chronic kidney disease), and (2) the proportion of clinic encounters with documented antihypertensive prescriptions in the EHR (ie, prescriptions for antihypertensive medicines generated electronically using the PIMS). The trial was registered at ClinicalTrials.gov (NCT05414526) and is complete.

**Findings:**

Between Jan 13, 2023, and Sept 10, 2025, 4654 participants were enrolled (2327 per group). At 12 months, blood pressure control among participants receiving antihypertensive medication was attained in 719 (67·1%) of 1072 participants in the intervention group versus 593 (51·5%) of 1152 in the standard-of-care group (risk ratio [RR] 1·29, 95% CI 1·10–1·51; p=0·0013 unadjusted). The prescription for antihypertensive medicines was issued electronically to 428 (39·9%) of the 1072 eligible encounters in the intervention group compared with 124 (10·8%) of the 1152 in the standard-of-care group (RR 5·95, 2·18–16·2; p=0·0005 unadjusted). At 12 months, serious adverse events were infrequent and did not differ significantly between the intervention and standard-of-care groups (22 [0·9%] of 2127 *vs* 20 [0·9%] of 2188; p=0·69), and viral suppression rates also did not differ significantly between the two groups (1759 [98·6%] of 1784 *vs* 1821 [99·0%] of 1839; p=0·39).

**Interpretation:**

Integrating hypertension care into HIV care programmes is an effective way to achieve blood pressure control and to increase the prescription of antihypertensive medicines electronically among people with HIV without compromising HIV outcomes.

## Introduction

WHO estimates that 1·4 billion adults worldwide were living with hypertension in 2024 and that hypertensidon contributes to 10 million deaths every year.^1^ Treatment coverage is lowest in the WHO African region, being estimated at 27%, and among those on treatment, only 12% had attained blood pressure control in 2019.^2^ Effective treatment of at least 50% of adults living with hypertension could avert an estimated 76 million hypertension-related deaths, 120 million strokes, and 97 myocardial infarctions by 2050.^1^

Sub-Saharan Africa has the highest proportion of people living with HIV globally.^3^ People with HIV are experiencing a rising burden of hypertension.^4^ In Botswana, hypertension prevalence and control rates among people with HIV across 22 communities were estimated to be 27% and 42%, respectively, in 2017–18.^5^ The technical package for the management of hypertension (WHO HEARTS) emphasises an adequate supply of antihypertensive medicines, using automated electronic blood pressure machines, team-based care, and digital information systems for monitoring blood pressure at the individual patient level plus the health system level.^6^ There is insufficient evidence to adopt some or all the elements of the WHO HEARTS technical package for the integration of HIV and hypertension care within the HIV service delivery platform. The 2025 WHO guideline on HIV service delivery recommendation for HIV and hypertension integration is based on moderate certainty evidence for blood pressure control within HIV service platforms and is silent on the electronic prescription of antihypertensive medicines where HIV care is delivered using an electronic health record (EHR) system,^4^ but the Botswana non-communicable disease strategy (2018–23) encourages electronic prescribing to track demand and efficiently inform the prediction of medication requirements.^7^ The WHO guideline on HIV service delivery acknowledges that integration carries risks to HIV outcomes;^4^ thus, integrated care requires careful evaluation.

The present study, Integrating Hypertension Care into the Existing HIV Services Package in Botswana (InterCARE), was designed to assess the effectiveness of a set of multicomponent and multilevel implementation strategies that deliver integrated hypertension care into the existing HIV services package in Botswana, aiming to improve blood pressure control and to adopt the electronic prescription of antihypertensive medicines using an adapted HIV EHR system.

## Methods

### Study design

We conducted a type 2 hybrid effectiveness–implementation, pair-matched, cluster-randomised clinical trial across 14 peri-urban and rural HIV clinics in Botswana. The sites were randomly selected from a total of 30 pair-matched sites from the completed Botswana Combination Prevention Project (BCPP).^3^ The trial was approved by the University of Botswana and the Botswana Ministry of Health ethics committees (HPRD6/14/1). The trial was registered at ClinicalTrials.gov (NCT05414526) and is complete. The protocol has been published.^8^ To prepare for the current trial, a single-arm pilot implementation study of 290 adults with HIV and hypertension was conducted between October, 2021, and November, 2022,^9^ at two geographically distant HIV clinics in Botswana that had a functional EHR system. These two pilot communities did not participate in the main trial.

The trial was implemented in 14 clinics in southern and northeastern Botswana. HIV clinics were eligible if they provided comprehensive HIV services, used the Patient Integrated Medical Record System (PIMS), the national EHR platform for HIV care in Botswana, and were located in communities that had participated in the BCPP.^3^ For additional details of inclusion and exclusion criteria, see appendix 2 (pp 3–4).

An independent Data Safety Monitoring Board provided oversight, and the Research Coordinating Centre^10^ conducted several in-person and virtual monitoring visits throughout the trial. All of the investigators vouched for the completeness and accuracy of the data, adherence to the trial protocol, and accurate reporting of adverse events. For additional details, see appendix 2 (pp 4–5).

### Participants

Eligible patients were adults aged 20–75 years with HIV who were taking antiretroviral therapy, who had a diagnosis of hypertension or whose blood pressure was elevated at screening (defined as a systolic blood pressure ≥140 mm Hg or a diastolic blood pressure ≥90 mm Hg, or ≥130/80 mm Hg among those with diabetes or chronic kidney disease), and who were receiving regular HIV care at the selected HIV clinics for more than 6 months at the time of enrolment. All participants provided written informed consent. Patients with HIV-associated dementia or who were unable or unwilling to provide written informed consent were excluded.

Each eligible participant at the intervention site was encouraged to select a treatment partner who would assist them with high blood pressure and general cardiovascular care. Treatment partners were eligible if they were aged 18 years or older and were selected to be the participant’s treatment partner.

Biological sex was self-reported. Participant blood pressure status was derived from a mean of three blood pressure readings using a clinic electronic blood pressure machine from the right arm at each study visit in accordance with the recommendations of the American Heart Association for measuring blood pressure and recording it in mm Hg.^11^ Electronic prescriptions of antihypertensive medication were extracted from the PIMS at each study visit. For additional details of participant eligibility and study procedures, see appendix 2 (pp 4–5).

### Randomisation and masking

The clinics were pair-matched by catchment population size, antiretroviral therapy access, age structure, and geographical location, as per a previously completed cluster-randomised trial.^3^ To be included from the 14 original clinic pairs, two matched clinics were randomly assigned to intervention or standard of care through a simple randomisation method (flip of a coin) centrally, and only enrolled if both HIV clinics in the selected pair met the inclusion criteria. However, if one or both HIV clinics in the randomly selected pair did not meet the inclusion criteria, then the entire pair was excluded, and the next randomly selected pair was evaluated for inclusion until a total of seven pairs met inclusion criteria. Neither the patients, their treatment partners, nor their health-care providers were masked to group assignment. However, the participants and health-care workers at standard-of-care sites were not made aware of what was happening at the intervention sites to minimise contamination. For additional details, see appendix 2 (p 4).

### Procedures

The trial tested a multicomponent, multilevel implementation strategy operating at the community, clinic, and patient levels to deliver integrated care. Hypertension services were provided within HIV clinics in accordance with adapted Botswana primary care guidelines, with additional community-based support provided through treatment partners. To achieve this goal, implementation strategies were developed to include (1) health-care provider training and coaching; (2) adaptation of an existing EHR system for electronic documentation of blood pressure and cardiovascular disease counselling plus the generation of a prescription of antihypertensive medicines electronically in the EHRs; and (3) the use of community-based treatment partners to support people with HIV and hypertension.

Participants received integrated care during routine HIV clinic visits. These visits included a blood pressure measurement, waist and hip circumference measurement, cardiovascular risk assessment, lifestyle counselling (smoking cessation, physical activity, and healthy eating), anti-hypertensive medicine initiation or dose titration, and assessments of the patient’s level of knowledge on cardiovascular risk factors using the Heart Disease Fact Questionnaire (HDFQ).^12^

The health-care providers in this trial, mostly nurses together with medical officers, were trained to screen for hypertension, provide counselling, initiate and titrate antihypertensive treatment, and ensure follow-up care for both medical conditions at each subsequent HIV clinic visit. Training materials were developed jointly with the Ministry of Health and delivered using interprofessional education methods to promote collaborative care.^13,14^ The health-care providers underwent pre-training and post-training assessments at the time of site activation. Ongoing coaching through audit feedback on hypertension diagnosis, treatment initiation, and titration was available from the study team every month and then as needed from experienced colleagues within their HIV clinic when they reported to be confident in managing hypertension. Both medical doctors (non-specialists) and family nurse practitioners served as in-clinic experienced supporters of junior general nurses. Health-care providers were required to have a post-training score of more than 70%; those who did not repeated the training. Repeat training was conducted if there was 50–75% turnover (transfer) in health-care providers involved in the delivery of the intervention. Additionally, the EHR system was adapted to capture cardiovascular risk factors (eg, blood pressure, waist circumference, smoking), counselling on cardiovascular risk reduction (eg, smoking cessation, physical activity, healthy eating), and electronic prescriptions of antihypertensive medicines, which included updating the drop-down menu for a list of antihypertensive medicines in the EHR prescription menu.

All participants in the intervention group were encouraged to identify a supportive member from the community who then received education on hypertension plus training in a modified motivational interviewing-informed communication style to provide effective support for the index person living with HIV and hypertension. This community-based treatment partner strategy served to promote the integration of HIV and hypertension into the community (beyond the HIV clinic). These treatment partners received education and training in person or virtually once at the beginning of the intervention. A standard script was available for use by the HIV clinic health-care provider during in-person or virtual educational and skills training of the treatment partner. A short video on how to offer support was available for treatment partners to view at a convenient time. Treatment partners were contacted every month and encouraged to provide support for their selected participant until blood pressure was controlled for at least 3 months. However, if the participant’s blood pressure was controlled, treatment partners were only asked about the support they gave the participant every 6 months.

Standard-of-care clinic providers did not receive training or coaching from the InterCARE team. They were expected to provide hypertension care as described in the Botswana primary guidelines. They could offer integrated care or refer adults with HIV and hypertension to another non-HIV primary care clinic for separate high blood pressure care. Clinical and implementation outcomes at standard-of-care clinics were measured in the same way as they were for the intervention HIV clinics. Participants at the standard-of-care sites were not told to bring a community-based treatment partner to support them in attaining blood pressure control. The standard-of-care HIV clinic EHR system was not modified. Health-care providers continued to provide health services and had access to a pre-existing EHR that they could use to document care and prescribe HIV and antihypertensive medicines electronically.

Both intervention and standard-of-care clinics provided routine HIV services, including free medical consultations, laboratory testing, and antiretroviral therapy, typically 2–4 days per week (weekdays only). For additional details, see appendix 2 (p 4).

### Outcomes

The outcomes are based on the reach, effectiveness, adoption, implementation, and maintenance (RE-AIM) framework.^15^

The trial had two coprimary outcomes: (1) effectiveness: the proportion of individuals with HIV and diagnosed hypertension receiving antihypertensive medicine with controlled blood pressure at 12 months (systolic and diastolic blood pressure <140 mm Hg and <90 mm Hg, respectively; or <130 mm Hg and <80 mm Hg for those with diabetes or chronic kidney disease); (2) implementation: the proportion of clinical encounters in EHRs in which antihypertensive medicines are documented as prescribed if indicated at 12 months (ie, antihypertensive medicine prescriptions generated electronically on the PIMS).

A total of 12 secondary outcomes were also assessed across all five RE-AIM domains (appendix 2, pp 5–6).

Adverse events were graded using the National Institutes of Health Department of AIDS adverse event grading scale,^16^ while unintended effects of integrated HIV and hypertension care were reported using HIV viral suppression rates.

### Statistical analysis

The primary intention-to-treat population included only participants on antihypertensive medicines. However, those who missed their 12-month study visit and yet were able to attend a future study visit were excluded from the intention-to-treat population. The primary per-protocol population (a subgroup of the primary intention-to-treat population) excluded participants with major protocol violations or sentinel events before the 12-month study visit. Major protocol violations included participant withdrawal from the trial, loss to follow-up, and incarceration and death as a sentinel event.

The secondary intention-to-treat and per-protocol populations at 12 months included all participants who were randomised (not just those on antihypertensive medicines), and these populations were defined in the same way as the primary intention-to-treat and per-protocol populations. Secondary intention-to-treat and per-protocol populations were analysed for the additional secondary outcomes only.

No interim analysis was planned, and no imputation for missing outcomes was planned.

The coprimary outcomes were analysed using generalised linear mixed models (GLMMs) with a Poisson distribution with robust standard errors and log link function, and the resulting unadjusted risk ratios (RRs) and associated 95% CIs were reported. The GLMMs included treatment as a fixed effect and cluster and pair as random effects. The main conclusions were drawn from the unadjusted results of the coprimary outcomes on the primary intention-to-treat population at the 12-month study visit. In addition, the above GLMMs were further adjusted for age, sex, level of income, waist–hip ratio, physical activity (estimated number of minutes walked per week), and alcohol and tobacco consumption to calculate the adjusted RRs with their 95% CIs.

Subgroup analysis of the two coprimary outcomes was conducted for eight covariates that are important for blood pressure control, namely, age, sex, education level, income, waist–hip ratio, physical activity, cigarette smoking, and alcohol consumption, using the same GLMM as in the primary endpoint analysis. To assess the heterogeneity of the treatment effect across categories of a subgroup variable, an interaction term between the treatment and the subgroup was added to the GLMM, and an associated p value for the interaction test was reported.

All secondary binary outcomes were analysed and reported in the same way as for the primary outcome analyses. Analysis of secondary continuous outcomes was performed using GLMMs with a normal distribution and identity link function. The models had the treatment as a fixed effect and cluster and pair as random effects. The mean differences with 95% CIs between the intervention and standard-of-care groups were derived from the GLMM. All analyses were conducted using R statistical software version 4.4.2.

Additional detailed information on sample size and power calculations and sensitivity analysis is provided in appendix 2 (pp 6–7).

### Role of the funding source

The funder contributed to study design but had no role in data collection, data analysis, data interpretation, or writing of the report.

## Results

Between Jan 13, 2023, and Sept 10, 2025, 4654 patients in 14 HIV clinics (2327 at seven intervention HIV clinics and 2327 at seven standard-of-care HIV clinics) were enrolled. The numbers of patients who declined to participate or did not state a reason for not participating were 40 and five respectively, in the intervention group and 59 and 16, respectively, in the standard-of-care group. A total of 205 (4·4%) participants had discontinued participation by 12 months: 117 (5·0%) in the intervention group and 88 (3·8%) in the standard-of-care group (figure 1). A detailed CONSORT diagram for up to 24 months of follow-up is provided in appendix 2 (p 8) and HIV clinic pair allocation comparison in InterCARE versus the parent trial (BCPP) is also provided in appendix 2 (p 9).

The baseline characteristics for the primary and secondary intention-to-treat population analyses, which included all participants randomly assigned at baseline, were generally similar between the intervention and standard-of-care groups, with a few imbalances reported (table 1). Additional baseline characteristics are presented in appendix 2 (pp 10–12).

Primary intention-to-treat analysis showed that at 12 months, among those receiving antihypertensive medication, the intervention group had a significantly greater proportion of participants with blood pressure control (719 [67·1%] of 1072) than the standard-of-care group (593 [51·5%] of 1152; RR 1·29, 95% CI 1·10–1·51; p=0·0013 unadjusted). The prevalence of antihypertensive medicine prescriptions in the EHRs was significantly greater in the intervention group (428 [39·9%] of 1072) than in the standard-of-care group (124 [10·8%] of 1152; RR 5·95; 2·18–16·2; p=0·0005 unadjusted; table 2). Covariate adjusted analyses showed similar results. The primary per-protocol analysis results for the two coprimary outcomes were similar to the primary intention-to-treat analysis results (table 2). In the post-hoc sensitivity analysis for assessing random cluster-level characteristics, the effects of the intervention remained significantly better: RR 1·23 (95% CI 1·04–1·45; p=0·018) and RR 6·55 (2·23–19·78; p=0·0007; appendix 2 p 38). In analysis assessing fixed cluster-level characteristics, the effect of the intervention was different: RR 1·27 (1·11–1·44; p=0·0003) for the effectiveness outcome and RR 5·55 (95% CI 2·57–12·0; p=0·0001) for the implementation outcome (appendix 2 pp 39, 41). Post-hoc pairwise comparisons showed similar intervention effects across clinic pairs, except for a greater effect in clinic pair 6 (peri-urban) than pair 1 (rural), RR 0·69 (95% CI 0·50–0·94; appendix 2 pp 40, 42).

The effect of the intervention did not differ across all the subgroups for either coprimary outcome (figure 2). There was a non-significant trend towards attaining blood pressure control among participants who were older than 40 years, were female, had a monthly income of P5000 or less, had a normal waist–hip ratio, reported alcohol use, and did not use tobacco. In sensitivity analysis, only blood pressure control was better among females and those older than 40 years: RR 1·26 (95% CI 1·10–1·44) and 1·30 (1·06–1·59), respectively (appendix 2 p 39).

Compared with the standard-of-care group, five of the 12 RE-AIM secondary outcomes were significantly better in the intervention group (table 3): one measure of reach, one measure of effectiveness, two measures of adoption, and one measure of maintenance at 12 months. The same outcomes remained significantly better in the intervention group than in the standard-of-care group at different timepoints (primary intention-to-treat and per-protocol analyses), as reported numerically and by line graphs (where appropriate) in appendix 2 (pp 13–22, 26–33).

Secondary intention-to-treat analysis at the 12-month follow-up revealed that a significantly greater proportion of participants in the intervention group attained blood pressure control (1619 [76·1%] of 2127) than did participants receiving standard-of-care (1132 [51·7%] of 2188; RR 1·44, 95% CI 1·22–1·69; p<0·0001 unadjusted; table 4). Participants in the intervention group were significantly more likely to receive counselling on hypertension diagnosis, recommended physical activity level, and recommended diet for people with hypertension. Participants in the intervention were also more likely to report engaging in more than 150 min of physical activity per week (520 [24·4%] of 2127; RR 17·8, 95% CI 4·54–70·0, p<0·0001 unadjusted; table 4). A high proportion of participants in the intervention group reported receiving support from their treatment partner (1715 [80·6%] of 2127). The results of the secondary per-protocol analysis at 12 months were similar to those of the intention-to-treat analysis (table 4).

At 12 months, serious adverse events were infrequent in both the intervention group (22 [0·9%] of 2127) and the standard-of-care group (20 [0·9%] of 2188; p=0·69, secondary intention-to-treat analysis). Among those with available viral load results, HIV viral suppression rates did not differ between the intervention group (1759 [98·6%] of 1784) and the standard-of-care group (1821 [99·0%] of 1839; p=0·39). Detailed safety analysis results up to 24 months of follow-up are provided in appendix 2 (pp 25, 37).

## Discussion

The InterCARE trial demonstrated that integrating hypertension and HIV care during routine HIV clinic visits was superior to standard of care. Participants in the intervention group were 29% more likely to attain blood pressure control and approximately five times more likely to receive electronic prescriptions for antihypertensive medicines than those in the standard-of-care group. This estimation translates to 67% blood pressure control within an HIV clinic, which is slightly lower than the 80% control reported among people with HIV in a community-based study in Kenya and Uganda.^17^ The per-protocol results were similar. HIV viral suppression rates remained constant across the trial groups from the beginning to the end of the trial.

Several mechanisms could explain the observed effects of the InterCARE strategies, as framed by the RE-AIM implementation framework. Reach was demonstrated by a greater proportion of participants who were not on antihypertensive medicine attaining blood pressure control in the intervention versus standard-of-care group, likely reflecting the use of non-pharmacological, evidence-based lifestyle interventions for blood pressure control given the focus on counselling.^18^ Effectiveness was also evident from a greater mean decrease in systolic blood pressure (6·8 mm Hg), which was modestly lower than the reduction observed in the InterCARE pilot study (10·3 mm Hg decrease in systolic blood pressure),^9^ and greater cardiovascular health knowledge, with 80·2% of participants in the intervention group having a passing score on the HDFQ^12^ (similar to 80% passing in the InterCARE pilot study).^9^ Adoption was reflected by expanded clinical practices, including attending an integrated hypertension–HIV clinic visit, having a 10-year cardiovascular disease risk calculated in the EHRs (which occurred in zero participants in the InterCARE pilot),^9^ and a waist–hip ratio documented in the EHRs. These modest levels in which an HIV clinic-based provider adopted integrated HIV care delivery provide preliminary evidence for study factors associated with or without adoption and the impact on the providers, such as fatigue and workload. Maintenance was evidenced by sustained blood pressure control at subsequent HIV clinic visits and consistent delivery of all intervention components across implementation sites. This finding is consistent with the reported modest levels of reach and adoption that were at least maintained for up to 24 months. Collectively, these indicators highlight how pragmatic, multilevel integration strategies can achieve measurable implementation and clinical gains, even within resource-constrained HIV care settings.

Hypertension awareness in the InterCARE trial was high (>93%) and did not differ by group (reach), which is consistent with findings from the InterCARE pilot study^9^—and was more than twice the level reported among people with HIV in Uganda.^19^ Therefore, treatment success (intervention) and non-success (standard of care) outcomes might not be driven by a lack of awareness of hypertension diagnosis in Botswana. The low level of guideline-concordant antihypertensive medicine prescriptions (52·6% intervention, 53·7% standard of care; fidelity) highlights the limited impact of InterCARE despite the health-care worker training and coaching. This result is slightly better than the 32% guideline-concordant antihypertensive medicine prescription in Ghana.^20^ The lack of difference between the two groups in the proportion of participants who reported receiving antihypertensive medicines (85·9% intervention, 90·1% standard of care; reach) or prescribed antihypertensive medicine (100% intervention, 100% standard of care; adoption) indicates that more data are needed to study the adherence to guidelines on medicine prescription and titration,^21^ adherence patterns, and non-pharmacological effects that produced better blood pressure control in the intervention group. The rates of prescription and reported use (>85%) of antihypertensive medicines are higher than what has been reported from 54 public sector hospitals in South Africa.^21^ These indicators highlight persistent gaps in hypertension treatment fidelity that might have limited the full potential impact of InterCARE. Planned secondary data analyses and post-implementation surveys and interviews will provide additional explanatory insights.

In the secondary intention-to-treat analysis, the beneficial effect of the InterCARE package on blood pressure control reaffirmed InterCARE as a superiority trial. The InterCARE intervention package contributed to better blood pressure control using established effective, scalable, evidence-based blood pressure control interventions (education and counselling to adopt strategies to manage cardiovascular risk factors, social support, etc) without increasing the use of antihypertensive medications.

There are several limitations to our study. Analysing our primary outcomes only among those on antihypertensive medicines reduced the number of participants in the analysis to only a subpopulation of all randomised participants (excluding those who might be using evidence-based lifestyle [biobehavioural] interventions).^18^ However, the effect of InterCARE on blood pressure control was maintained in a secondary intention-to-treat analysis that included all randomised participants, which revealed that it was possible to attain 70% blood pressure control as set out in the Botswana non-communicable disease strategy.^7^ Early implementation activities (interviews, surveys, and health-care worker training for all HIV clinics randomised to intervention) before the first participant was enrolled might have contributed to significantly better baseline blood pressure control in the intervention group. Nevertheless, significantly better blood pressure control was sustained for more than 24 months. Because community matching characteristics were based on factors associated with HIV burden^3^ and not primarily factors associated with hypertension, these factors might explain the minor imbalances observed between the trial groups. Despite these imbalances, the adjusted results remain similar to the unadjusted results. Of note, our study design allowed us to effectively assess how variability in the burden of HIV across the health-care system could impact the integration of hypertension care during HIV clinic visits while maintaining HIV viral suppression. It is possible that health-care workers transferred or communicated, and participants from intervention sites could have interacted with those at standard-of-care sites; thus, it is possible that some aspects of the intervention package were also implemented at standard-of-care sites. Lastly, we detected minimal statistically significant differences in the subgroup analysis, likely because our study was not powered to assess treatment heterogeneity.

Our results add to similar findings of successful integrated care in sub-Saharan Africa as reported in the INTE-AFRICA and INTE-COMM cluster-randomised trials on integrated HIV, diabetes, and hypertension care.^22,23^

In summary, this study shows that in high-HIV-prevalence, resource-limited settings, training and coaching of HIV health-care providers to integrate high blood pressure care for people with HIV and hypertension during an HIV clinic visit is effective in increasing blood pressure control, and leads to more electronic prescriptions. Beyond hypertension care, future studies should evaluate the effect of incremental integration of other non-communicable disease services for people with HIV, and leverage electronic data from integrated services to inform policies on health services planning and forecasting resources.

## Supplementary Material

1

2

3

## Figures and Tables

**Figure 1:**
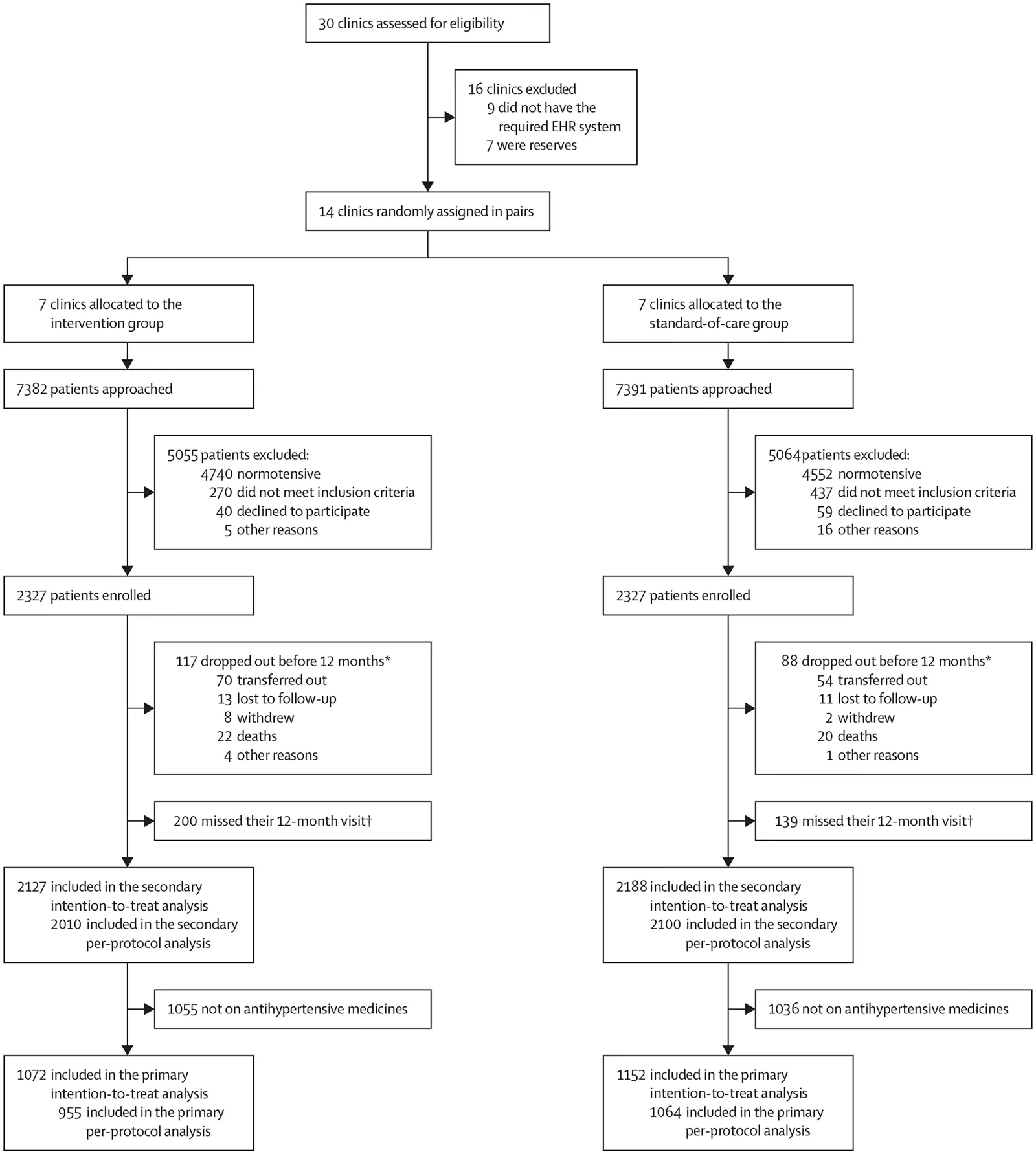
Trial profile EHR=electronic health record. *These patients were excluded from the per-protocol analyses. †These patients were excluded from the intention-to-treat and per-protocol analyses.

**Figure 2:**
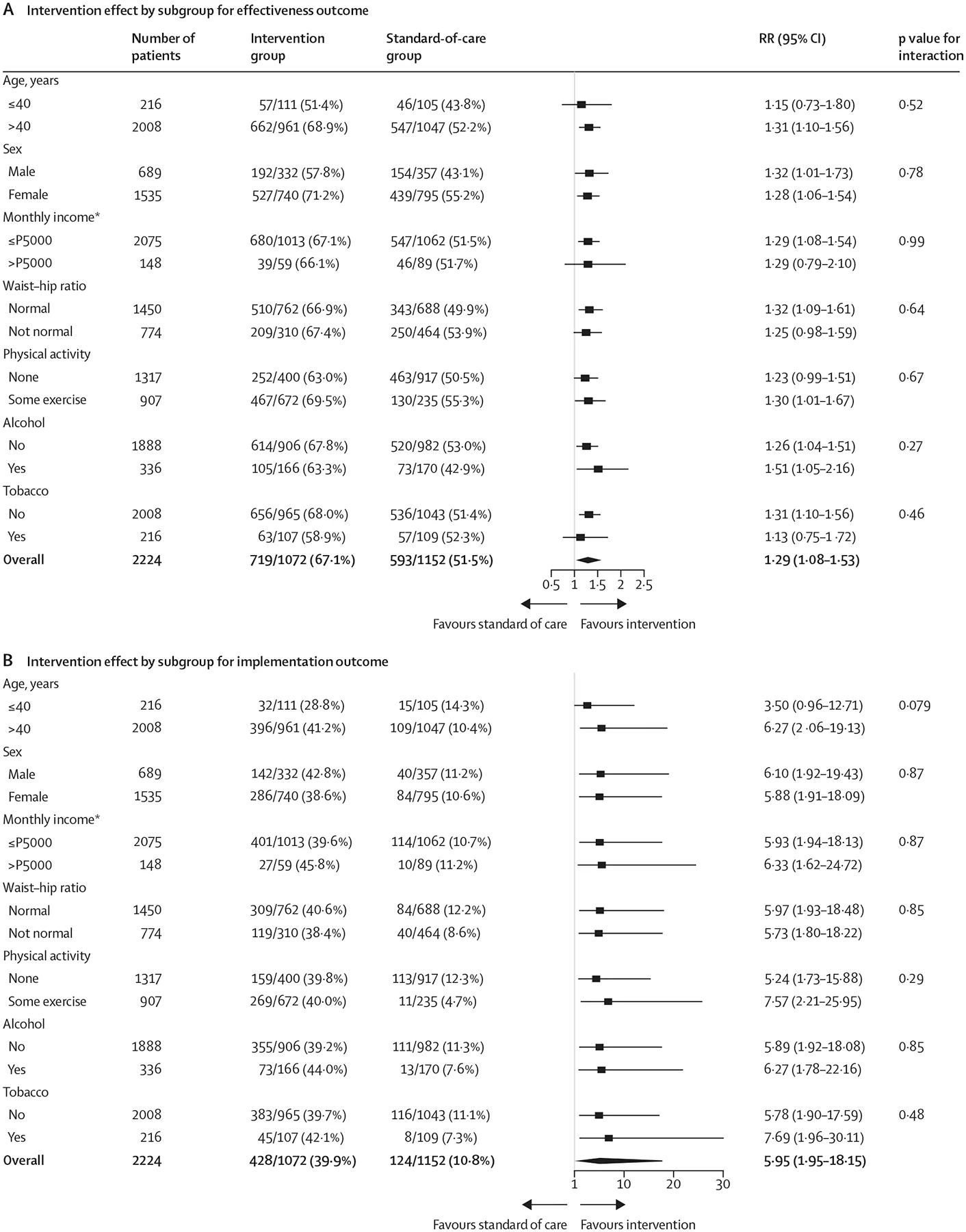
Subgroup analysis of the effectiveness (A) and implementation (B) coprimary outcomes Subgroup analysis was conducted for the covariates (age, sex, monthly income, waist–hip ratio, physical activity, and alcohol and tobacco consumption) using the generalised linear mixed model (GLMM). To assess the heterogeneity of the treatment effect across categories of a subgroup variable, an interaction term between the treatment and the subgroup was added to the GLMM, and an associated p value for the interaction test was reported. RR=risk ratio. *P=pula (Botswana currency; ISO 4217 code BWP).

**Table 1:** Baseline characteristics of the participants

	Primary intention-to-treat population				Secondary intention-to-treat population			
	All (n=2224)	Intervention group (n=1072)	Standard-of-care group (n=1152)	SMD	All (n=4315)	Intervention group (n=2127)	Standard-of-care group (n=2188)	SMD
**Sociodemographics**								
Sex	··	··	··	0·0004	··	··	··	0·032
Female	1535 (69·0%)	740 (69·0%)	795 (69·0%)	··	2651 (61·4%)	1290 (60·6%)	1361 (62·2%)	··
Male	689 (31·0%)	332 (31·0%)	357 (31·0%)	··	1664 (38·6%)	837 (39·4%)	827 (37·8%)	··
Age in years, mean (SD)	54·0 (10·3)	53·5 (10·5)	54·4 (10·1)	0·084	51·3 (11·0)	50·7 (11·1)	51·8 (10·8)	0·098
Marital status	··	··	··	0·11	··	··	··	0·072
Single	1435 (64·5%)	720 (67·2%)	715 (62·1%)	··	3084 (71·5%)	1548 (72·8%)	1536 (70·2%)	··
Married	543 (24·4%)	246 (22·9%)	297 (25·8%)	··	903 (20·9%)	436 (20·5%)	467 (21·3%)	··
Divorced or separated	72 (3·2%)	32 (3·0%)	40 (3·5%)	··	94 (2·2%)	41 (1·9%)	53 (2·4%)	··
Widowed	174 (7·8%)	74 (6·9%)	100 (8·7%)	··	234 (5·4%)	102 (4·8%)	132 (6·0%)	··
**Hypertension history**								
Aware of hypertension	··	··	··	0·086	··	··	··	0·50
Yes	2194 (98·7%)	1063 (99·2%)	1131 (98·2%)	··	3978 (92·2%)	2101 (98·8%)	1877 (85·8%)	··
No	30 (1·3%)	9 (0·8%)	21 (1·8%)	··	337 (7·8%)	26 (1·2%)	311 (14·2%)	··
Duration of hypertension diagnosis in years, median (IQR)	4·90 (1·14–10·7)	3·86 (0·62–9·83)	5·89 (1·90–11·4)	0·089	1·24 (0·17–6·39)	0·91 (0·00–4·99)	1·97 (0·41–7·96)	0·11
Duration of hypertension medicines in years, median (IQR)	6·0 (2·1–11·8)	5·4 (2·0–11·8)	6·1 (2·4–11·9)	0·057	5·92 (2·15–11·71)	5·33 (2·04–11·67)	6·14 (2·39–11·82)	0·060
Number of antihypertensives, median (IQR)	2·0 (1·0–2·0)	1·0 (1·0–2·0)	2·0 (1·0–2·0)	0·21	2·0 (1·0–2·0)	1·0 (1·0–2·0)	2·0 (1·0–2·0)	0·15
Number of prescribed antihypertension medicine classes	··	··	··	0·082	··	··	··	0·071
One drug class	944/1835 (51·4%)	432/817 (52·9%)	512/1018 (50·3%)	··	989/1895 (52·2%)	443/834 (53·1%)	546/1061 (51·5%)	··
Two drug classes	748/1835 (40·8%)	322/817 (39·4%)	426/1018 (41·8%)	··	763/1895 (40·3%)	328/834 (39·3%)	435/1061 (41·0%)	··
Three drug classes	132/1835 (7·2%)	56/817 (6·9%)	76/1018 (7·5%)	··	132/1895 (7·0%)	56/834 (6·7%)	76/1061 (7·2%)	··
Four or more drug classes	11/1835 (0·6%)	7/817 (0·9%)	4/1018 (0·4%)	··	11/1895 (0·6%)	7/834 (0·8%)	4/1061 (0·4%)	··
**HIV parameters**								
HIV duration in years, median (IQR)	11·8 (7·28–16·5)	10·8 (6·90–15·5)	12·6 (8·05–17·1)	0·20	10·8 (6·9–15·4)	10·3 (6·7–15·0)	11·3 (7·2–16·0)	0·13
**Blood pressure measurements at baseline**								
Right arm SBP, mm Hg	136·0 (125·0–147·0)	136·0 (125·0–146·0)	136·0 (124·0–148·0)	0·069	140·0 (129·0–147·0)	139·0 (130·0–145·0)	140·0 (129·0–150·0)	0·16
Right arm DBP, mm Hg	84·0 (76·0–91·0)	83·0 (76·0–90·0)	84·0 (76·0–91·0)	0·065	85·0 (78·0–91·0)	84·0 (78·0–90·0)	87·0 (79·0–92·0)	0·19
Left arm SBP, mm Hg	136·0 (125·0–147·0)	135·5 (124·0–145·0)	136·0 (125·0–148·0)	0·11	140·0 (130·0–147·0)	139·0 (130·0–145)	141·0 (129·0–149·0)	0·18
Left arm DBP, mm Hg	83·0 (76·0–91·0)	82·0 (76·0–90·0)	84·0 (76·0–91·0)	0·11	85·0 (79·0–91·0)	84·0 (78·0–90·0)	87·0 (79·0–92·0)	0·23
Controlled blood pressure (yes)	1121 (50·4%)	554 (51·7%)	567 (49·2%)	0·049	1765 (40·9%)	960 (45·1%)	805 (36·8%)	0·17
**Other parameters**								
Waist circumference in cm, median (IQR)	89·5 (81·0–99·1)	88·0 (78·0–96·0)	92·0 (83·0–101·6)	0·31	88·0 (79·0–97·0)	86·0 (77·0–95·0)	90·0 (81·3–99·1)	0·39
Tobacco use (yes)	216 (9·7%)	107 (10·0%)	109 (9·5%)	0·018	551 (12·8%)	273 (12·8%)	278 (12·7%)	0·0040
Alcohol use (yes)	336 (15·1%)	166 (15·5%)	170 (14·8%)	0·020	869 (20·1%)	423 (19·9%)	446 (20·4%)	0·012
Cardiovascular risk estimation in PIMS (yes)	106 (4·8%)	101 (9·4%)	5 (0·4%)	0·43	181 (4·2%)	172 (8·1%)	9 (0·4%)	0·39

Data are n (%) or n/N (%) unless otherwise specified. SMD=standardised mean difference. SBP=systolic blood pressure. DBP=diastolic blood pressure. PIMS=Patient Integrated Medical Record System.

**Table 2:** Blood pressure control and antihypertensive medicine electronic prescription at 12 months among participants who were prescribed antihypertensive medicines

	Intervention group	Standard-of-care group	Unadjusted risk ratio (95% CI)*	p value	Adjusted risk ratio (95% CI)†	p value
**Primary intention-to-treat population**						
Blood pressure control	719/1072 (67·1%)	593/1152 (51·5%)	1·29 (1·10–1·51)	0·0013	1·25 (1·07–1·46)	0·0053
Antihypertensive medicine electronic prescribing	428/1072 (39·9%)	124/1152 (10·8%)	5·95 (2·18–16·2)	0·0005	5·81 (2·11–16·0)	0·0007
**Primary per-protocol population**						
Blood pressure control	719/955 (75·3%)	593/1064 (55·7%)	1·33 (1·13–1·57)	0·0006	1·30 (1·10–1·54)	0·0019
Antihypertensive medicine electronic prescribing	428/955 (44·8%)	124/1064 (11·7%)	6·09 (2·29–16·2)	0·0003	6·11 (2·31–16·1)	0·0003

* Risk ratios with 95% CIs were calculated from generalised linear mixed models (GLMMs) with a Poisson distribution and log link function and with the treatment (treatment *vs* control) as a fixed effect and clusters and pairs as random effects.

† The GLMMs used to calculate the unadjusted values were further adjusted for age, sex, level of income, waist–hip ratio, physical activity (estimated number of minutes walked per week), and alcohol and tobacco consumption.

**Table 3:** Secondary outcomes for the primary intention-to-treat population at 12 months

	Intervention group (n=1072)	Standard-of-care group (n=1152)	Unadjusted treatment effect (95% CI)*	p value
**Reach**				
Proportion aware of hypertension status	882 (82·3%)	997 (86·5%)	0·96 (0·87–1·05)	0·34
Proportion with diagnosed hypertension receiving antihypertensive medicine	921 (85·9%)	1038 (90·1%)	0·95 (0·87–1·04)	0·29
Proportion with blood pressure control not on medicines	908/1055 (86·1%)	539/1036 (52·0%)	1·64 (1·37–1·95)	<0·0001
**Effectiveness**				
Decrease in mean SBP, mm Hg (95% CI)	−6·79 (−7·64 to −5·94)	−2·54 (−3·59 to −1·50)	−4·06 (−7·21 to −0·91)	0·016
Decrease in mean DBP, mm Hg (95% CI)	−3·49 (−4·03 to −2·96)	1·54 (−2·98 to 6·06)	−5·08 (−11·4 to 1·21)	0·10
Proportion with >70% pass on HDFQ†	860 (80·2%)	640 (55·6%)	1·41 (0·93–2·15)	0·11
**Adoption**				
Proportion receiving HIV and hypertension care during same clinic visit	855 (79·8%)	509 (44·2%)	1·99 (1·36–2·93)	<0·0001
Proportion with 10-year cardiovascular disease risk calculated in the EHRs	469 (43·8%)	1 (0·1%)	437·96 (61·49–3119·16)	<0·0001
Proportion of participants prescribed antihypertensive medicine	1072 (100%)	1152 (100%)	1·00 (0·92–1·09)	>0·90
Proportion with waist and hip circumference recorded in the EHRs	590 (55·0%)	0	NA	NA
**Implementation fidelity**				
Proportion on medication with correct regimen per national guideline	564 (52·6%)	619 (53·7%)	0·99 (0·86–1·14)	0·86
**Maintenance**				
Proportion of intervention clinics that implemented all three InterCARE strategies†	7/7 (100%)	NA	NA	NA
Proportion enrolled in InterCARE with blood pressure control (at baseline) who maintain blood pressure control in the next study visit	725 (67·6%)	598 (51·9%)	1·29 (1·10–1·51)	0·0013

Data are n (%) or n/N (%) unless otherwise specified. SBP=systolic blood pressure. DBP=diastolic blood pressure. HDFQ=Heart Disease Fact Questionnaire. EHR=electronic health record. NA=not applicable.

* For binary outcomes, the unadjusted risk ratio with 95% CI was calculated as a measurement of the treatment effect from a generalised linear mixed model (GLMM) with a Poisson distribution and log link function and with the treatment (intervention *vs* standard of care) as a fixed effect and clusters and pairs as random effects. For continuous outcomes, the mean difference with 95% CI was calculated from the GLMM with a normal distribution and identity link function and with the treatment as a fixed effect and clusters and pairs as random effects.

† Assessed at 24 months as per the statistical analysis plan.

**Table 4:** Overall impact of the intervention on blood pressure control, cardiovascular disease risk counselling, self-reported physical activity level, and treatment partner support

	Intervention group	Standard-of-care group	Unadjusted risk ratio (95% CI)*	p value
**Secondary intention-to-treat population**				
Blood pressure control	1619/2127 (76·1%)	1132/2188 (51·7%)	1·44 (1·22–1·69)	<0·0001
Counselling on antihypertensive medicines†	592/1072 (55·2%)	391/1155 (33·9%)	2·32 (1·32–4·06)	0·0034
Counselling on physical activity	1531/2126 (72·0%)	321/2188 (14·7)	4·50 (1·50–13·5)	0·0071
Counselling on diet	1560/2127 (73·3%)	470/2188 (21·5%)	4·64 (1·49–14·5)	0·0080
Self-reported physical activity >150 min per week	520/2127 (24·4%)	39/2188 (1·8%)	17·8 (4·54–70·0)	<0·0001
Treatment partner support	1715/2127 (80·6%)	NA	NA	NA
**Secondary per-protocol population**				
Blood pressure control	1619/2010 (80·5%)	1132/2100 (53·9%)	1·46 (1·24–1·72)	<0·0001
Counselling on antihypertensive medicines†	592/955 (62·0%)	391/1067 (36·6%)	2·80 (1·28–6·16)	0·0103
Counselling on physical activity	1531/2009 (76·2%)	321/2100 (15·3%)	4·57 (1·51–13·8)	0·0071
Counselling on diet	1560/2010 (77·6%)	470/2100 (22·4%)	4·72 (1·49–14·9)	0·0082
Self-reported physical activity >150 min per week	520/2010 (25·9%)	39/2100 (1·9%)	18·2 (4·65–70·9)	<0·0001
Treatment partner support	1715/2010 (85·3%)	NA	NA	NA

Data are n/N (%) unless otherwise specified. NA=not applicable.

* Unadjusted risk ratios and 95% CIs were calculated from a generalised linear mixed model with a Poisson distribution and log link function and with the treatment (intervention *vs* standard of care) as a fixed effect and clusters and pairs as random effects.

† Applies to only those on antihypertensive medicines.

## Data Availability

Published and de-identified data with the data dictionaries, study protocol, statistical analysis plan, and a sample of the informed consent form are available upon request to the corresponding author.

## Research in context

### Evidence before this study

We searched PubMed from database inception to Jan 31, 2026, for all studies on integrated HIV and hypertension care within HIV clinics using the keywords “integrated HIV care” AND “hypertension” OR “high blood pressure” with no language restrictions. A total of three studies, two in South Africa and one in Malawi, assessed integrated HIV and hypertension care during routine HIV clinic visits. There were no data on the electronic prescription of antihypertensive medicines in electronic health record (EHR) systems developed for HIV care in sub-Saharan Africa. The search was then expanded to include studies if they reported on hypertension prevalence, control, or management among people with HIV in any setting. From the 4627 articles identified, a systematic review and meta-analysis revealed that in sub-Saharan Africa, an estimated one in five people with HIV have hypertension and have low blood pressure control rates (13·4%).

### Added value of this study

This is the first large pragmatic trial that revealed an increase in both blood pressure control and the adoption of electronic prescriptions of antihypertensive medicines in EHR during an HIV clinic visit. The study provides evidence for integrating high blood pressure care during HIV clinic visits, including using an HIV EHR system, without negatively impacting on HIV outcomes in resource-limited settings with high HIV prevalence.

### Implications of all the available evidence

These findings, in addition to those concerning sustained viral suppression, provide strong evidence supporting the integration of hypertension care into HIV services and are supportive of updating the WHO HIV service delivery recommendations from moderate-certainty to high-certainty evidence for blood pressure management within HIV care platforms. The findings also support consideration of using electronic prescriptions for antihypertensive medicines where HIV services are offered through the EHR system.
