# Supplementary material for: Integrating hypertension care into the existing HIV services package in Botswana (InterCARE): a pair-matched, cluster-randomised, type 2 hybrid effectiveness–implementation trial
Source: Lancet Glob Health. Author manuscript; Available in PMC 2026 Oct 23. (PMC13691192; doi:10.1016/j.langlo.2026.104041)
Supplement: Supplementary file 1 [file NIHMS2215925-supplement-1.pdf]

THE LANCET
Global Health

Supplementary appendix 1

This translation in Setswana was submitted by the authors and we reproduce it as supplied. It has not been peer reviewed. *The Lancet*'s editorial processes have only been applied to the original in English, which should serve as reference for this manuscript.

Thanodi e ya Setswana re e filwe ke bakwadi ba patlisiso e, re e bega jaaka ba re e file. Ga e a kanokiwa ke baitsaanape. Bakwadi le bagatise ba Lancet ba sekasesike mokwalo o mo puong ya sekgowa fela, ke one o tla dirisiwang go ka kaya mokwalo o.

Supplement to: Mosepele M, Gaolathe T, Kebotsamang K, et al. Integrating hypertension care into the existing HIV services package in Botswana (InterCARE): a pair-matched, cluster-randomised, type 2 hybrid effectiveness–implementation trial. *Lancet Glob Health* 2026. https://doi.org/10.1016/j.langlo.2026.104041

InterCARE Abstract

Background/Ketapele: Batho ba ba tshelang le mogare wa HIV ga ba kgone go bona tlhokomelo ya makoa a bone sentle le fa bokete ja bolwetse le dintsho di oketsega. Re ne ra sekaseka seabe sa go tlhofofatsa le go kopanya tlhokomelo ya HIV le ya high blood mabapi le go laola blood pressure, ga mmogo le tiriso ya boranyane go kwalela balwetse melemo ya high blood le seabe sa gone mo go fokotseng bokete ja mogare wa HIV mo Botswana.

Methods/Ditsamaiso: Re ne ra dira tshekatsheko mo dikokelong tse di tlhokomelang batho ba ba tshelang le HIV di le 14 mo Botswana. Tshekatsheko e ne e dirwa mo bagolong ba dingwaga tse 20 go ya ko go tse 75 ba ba tshelang le HIV ebile ba na le high blood pressure. Go ne go akarediwa dikokelo tsa HIV tse di dirisang boranyane ja Patient Information Management System (PIMS). PIMS ke mofuta wa boranyane o o dirisiwang go tlhokmela mekwalo ya bongaka kgotsa ya botsogo ya balwetse ba HIV. Dikokelo di ne di bapisiwa ka bobedi-bobedi go ya ka lefelo le kokelo e leng mo go lone; palo ya batho ba lefelo leo, go nna teng ga melemo ya di-ARV, e e leng teng, dingwaga tsa batho ba lefelo leo. Go tswa foo, kokelo e le nngwe go tswa mo bobeding e ne e tsenngwa ka khupelekhupele mo setlhopheng sa lenaneo la tshekatsheko kgotsa lenaneo la gale la tlhokomelo/kalafi. Lenaneo la tshekatsheko le ne le akaretsa thuthuntsho ya badiri ba botsogo, go tswelela ka boakedi, tiriso ya badiredi kgotsa bapati ba kalafi ba sechaba le tiriso ya mekwalo ya bongaka ya balwetse ya boranyane go thusa go tlhomamisa bolwetse, tlhokomelo ka kakaretso le tiriso ya go kwalela balwetse melemo ka boranyane ka nako ya fa ba tlile maetong a tlhokomelo ya HIV. Maikalelo e ne e le go bona gore ka kgwedi ya bo lesome le bobedi, dipalo tsa batsayakarolo ba ba tsayang melemo ya highblood ba blood pressure ya bone e neng ya laolesega jaaka go ikaeletswe (balwetse ba sukiri kgotsa ba bolwetse ja diphilo) le dipalo tsa balwetse ba ba tlang ko kokelong ba go supagagalng ba kwaletswe melemo ya bone ya high blood ka tiriso ya boranyane (sekai, tiriso ya PIMS go kwalela molwetse melemo). Tshekatsheko e setse e wetse mme e kwadisitswe mo boranyaneg ja: ClinicalTrials.gov (NCT05414526).

Findings/Maduo: Fa gare ga kgwedi ya January 13 2023 le kgwedi ya September 10 2025, batsayakarolo ba le 4654 ba na ba kwadisiwa mo tshekatshekong. Ka kgwedi ya bo lesome le bobedi, taolo ya blood pressure mo batsayakarolong ba ba neng ba tsaya molemo wa high blood e ne ya fitlhelelwa mo go ba le 719 (kgotsa ba le 67 mo go ba le 100) go tswa mo go ba le 1072 ba lenaneo la tshekatsheko kgatlhanong mo go ba le 593 (kgotsa ba le 52 mo go ba le 100) mo go ba le 1152 ba lenaneo la gale la tlhokomelo/kalafi. Go kwalela balwetse melemo ka maranyane go ne ga dirwa mo balwetseng ba le 428(kgotsa balwetse ba le 40 mo go ba le 100) mo go ba le 1072 ba lenaneo la tshekatsheko fa go tshwangtshanngwa le ba le 124 (kgotsa ba le 11 mo go ba le 100) mo go ba le 1152 mo lenaeong la gale la tlhokomelo/kalafi. Ditlamorago tse di maswe di ne di sa diragale thata ebile di ne di sa farologane thata fa gare ga ba lenaneo la tshekatsheko le ba lenaneo la gale la tlhokomelo/kalafi ka kgwedi ya

lesome le bobedi. Go fokotsega kgotsa go wela tlase ga mogare le gone go ne go sa farologane thata fa gare ga ditlhopha ka bobedi.

Interpretation/Tlhaloso ya maduo: Go akaretsa tlhokomelo ya high blood mo lenaneong la tlhokomelo ya HIV ke tsela e e siameng ebile e bereka sentle thata go laola blood pressure le go oketsa tiriso ya go kwalela balwetse ba ba tshelang ka HIV melemo ya high blood ka boranyane go sa kgoreletse maduo a bone a botsogo.

Funding/Thuso tsa madi: Ke ba National Heart, Lung, and Blood Institute mo lekalaneng la US National Institutes of Health.
