# Supplementary material for: Integrating hypertension care into the existing HIV services package in Botswana (InterCARE): a pair-matched, cluster-randomised, type 2 hybrid effectiveness–implementation trial
Source: Lancet Glob Health. Author manuscript; Available in PMC 2026 Oct 23. (PMC13691192; doi:10.1016/j.langlo.2026.104041)

THE LANCET
Global Health

Supplementary appendix 2

This appendix formed part of the original submission and has been peer reviewed.
We post it as supplied by the authors.

Supplement to: Mosepele M, Gaolathe T, Kebotsamang K, et al. Integrating hypertension care into the existing HIV services package in Botswana (InterCARE): a pair-matched, cluster-randomised, type 2 hybrid effectiveness–implementation trial. *Lancet Glob Health* 2026. https://doi.org/10.1016/j.langlo.2026.104041

Study Title: Integrating hypertension and cardiovascular disease care into existing HIV services package in Botswana (InterCARE): a type 2 pair-matched hybrid effectiveness-implementation cluster randomized trial

SECTION 1: SUPPLEMENTARY METHODS & BASELINE CLINIC & PARTICIPANTS CHARACTERISTICS ... 3

SUPPLEMENTARY METHODS 1 ... 3
SUPPLEMENTARY FIGURE 1: FULL INTERCARE CONSORT DIAGRAM ... 8
SUPPLEMENTARY TABLE S1: PAIR ALLOCATION TO INTERVENTION VERSUS STANDARD-OF CARE FOR PARENT TRIAL (BCPP) AND CURRENT TRIAL (INTERCARE) ... 9
SUPPLEMENTARY TABLE S2: ADDITIONAL BASELINE CHARACTERISTICS OF THE STUDY PARTICIPANTS ... 10

SECTION 2: SECONDARY OUTCOMES FOR THE PRIMARY INTENTION-TO-TREAT (ITT) POPULATION ... 13

SUPPLEMENTARY FIGURE 2: PROPORTION OF PARTICIPANTS AWARE OF THEIR HYPERTENSION STATUS AT 6, 12, 18, 24 MONTHS STUDY VISIT (REACH) (PRIMARY INTENTION-TO-TREAT POPULATION) ... 13
SUPPLEMENTARY FIGURE 3: DECREASE IN MEAN SYSTOLIC AND DIASTOLIC BLOOD PRESSURES AT 6, 12, 18 AND 24 MONTHS VISIT RELATIVE TO THE BASELINE VALUES (EFFECTIVENESS) (PRIMARY INTENTION-TO-TREAT POPULATION) ... 14
SUPPLEMENTARY FIGURE 4: PROPORTION OF PARTICIPANTS RECEIVING HIV AND HYPERTENSION CARE AT SAME CLINIC DURING 6, 12, 18, 24 MONTHS VISIT (ADOPTION) (PRIMARY INTENTION-TO-TREAT POPULATION) ... 15
SUPPLEMENTARY FIGURE 5: PROPORTION OF PARTICIPANTS WITH >70% PASS ON HEART DISEASE FACT QUESTIONNAIRE (HDFQ) DURING THE 6, 12, 18 AND 24 MONTHS VISIT (EFFECTIVENESS) (PRIMARY INTENTION-TO-TREAT POPULATION) ... 16
SUPPLEMENTARY FIGURE 6: PROPORTION OF THE PARTICIPANTS WITH CONTROLLED BLOOD PRESSURE AT BASELINE WHO MAINTAIN BLOOD PRESSURE CONTROL IN NEXT STUDY VISIT: (MAINTENANCE) (PRIMARY INTENTION-TO-TREAT POPULATION) ... 17
SUPPLEMENTARY FIGURE 7: PROPORTION OF THE PARTICIPANTS WITH 10-YEAR CARDIOVASCULAR RISK CALCULATED IN THE ELECTRONIC HEALTH RECORDS (PRIMARY INTENTION-TO-TREAT POPULATION) ... 18
SUPPLEMENTARY FIGURE 8: PROPORTION OF THE PARTICIPANTS ON MEDICATIONS WITH CORRECT REGIMEN PER NATIONAL GUIDELINES (PRIMARY INTENTION-TO-TREAT POPULATION) ... 19
SUPPLEMENTARY FIGURE 9: PROPORTION OF THE PARTICIPANTS WITH WAIST AND HIP CIRCUMFERENCE RECORDED IN ELECTRONIC HEALTH RECORDS (PRIMARY INTENTION-TO-TREAT POPULATION) ... 20
SUPPLEMENTARY FIGURE 10: PROPORTION OF PARTICIPANTS WITH BLOOD PRESSURE CONTROL NOT ON ANTIHYPERTENSIVES AT 6, 12, 18 AND 24 MONTHS STUDY VISITS (REACH) (PRIMARY INTENTION-TO-TREAT POPULATION) ... 21
SUPPLEMENTARY FIGURE 21: PROPORTION OF PARTICIPANTS WITH BLOOD PRESSURE CONTROL NOT ON ANTI-HYPERTENSIVE MEDICATIONS AT 6, 12, 18 AND 24 MONTHS STUDY VISIT (REACH) (PRIMARY INTENTION-TO-TREAT POPULATION) ... 22
ABBREVIATIONS: SOC: STANDARD-OF-CARE ... 22
SUPPLEMENTARY FIGURE 11: PROPORTION OF PARTICIPANTS PRESCRIBED ANTI-HYPERTENSIVE MEDICATION AT 6, 12, 18 AND 24 MONTHS STUDY VISITS (SECONDARY INTENTION-TO-TREAT POPULATION) ... 23
SUPPLEMENTARY FIGURE 12: PROPORTION OF PARTICIPANTS RECEIVING ANTI-HYPERTENSION MEDICATION AT 6, 12, 18 AND 24 MONTHS STUDY VISITS (SECONDARY INTENTION-TO-TREAT POPULATION) ... 24
SUPPLEMENTARY TABLE S3: UNINTENDED EFFECTS OF INTERCARE (PRIMARY INTENTION-TO-TREAT POPULATION) ... 25

SECTION 3: SECONDARY OUTCOMES FOR THE PRIMARY PER PROTOCOL POPULATION ... 26

SUPPLEMENTARY FIGURE 13: PROPORTION OF PARTICIPANTS AWARE OF THEIR HYPERTENSION STATUS AT 6, 12, 18 AND 24 MONTHS STUDY VISITS (REACH) (PRIMARY PER-PROTOCOL POPULATION) ... 26

Supplementary Figure 14: Mean decrease in systolic and diastolic blood pressure at 6, 12, 18 and 24 months study visits (EFFECTIVENESS) (Primary per-protocol population) 27
Supplementary Figure 15: Proportion of participants receiving HIV and hypertension care at same clinic visit at 6, 12, 18 and 24 months study visit (Adoption) (Primary per-protocol population): 28
Supplementary Figure 16: Proportion of participants with >70% pass on Heart Disease Fact Questionnaire (HDFQ) (EFFECTIVENESS) (Primary per-protocol population) 29
Supplementary Figure 17: Proportion of participants with blood pressure control at baseline who maintain blood pressure control in next study visit: (MAINTENANCE) (Primary per-protocol population) 30
Supplementary Figure 18: Proportion of participants with 10-year cardiovascular risk calculated in the electronic health records (Primary per-protocol population). 31
Supplementary Figure 19: Proportion of participants on medications with correct regimen as per the national guidelines at 6, 12, 18 and 24 months study visits (Primary per-protocol population) 32
Supplementary Figure 20: Proportion of participants with waist and hip circumference recorded in Electronic Health Records at 6, 12, 18 and 24 months study visits (Primary per-protocol population) 33
Supplementary Figure 22: Proportion of participants prescribed anti-hypertensive medication at 6, 12, 18 and 24 months study visits (Secondary per-protocol population) 34
Supplementary Figure 23: Proportion of participants with receiving anti-hypertension medication at 6, 12, 18 and 24 months study visit (Secondary per-protocol population). 35
Supplementary Table S4: Proportion of intervention clinics that implemented all three InterCARE strategies at 24 months study visit (Primary intention-to-treat population) 36
Supplementary Table S5: Unintended effects of InterCARE at 12 and 24 months study visits (Secondary intention-to-treat population) 37

SECTION 4: SENSITIVITY ANALYSIS OF THE PRIMARY OUTCOMES 38

Supplementary Table S6: Sensitivity analysis of the primary outcomes adjusting for cluster level characteristics using the primary intention-to-treat population at 12 months study visit 38
Supplementary Table S7: Adjusted generalised linear mixed model for primary effectiveness outcome with clinic pairs as fixed effects 39
Supplementary Figure 23: Post-Hoc Pairwise Comparisons of Clinic Pairs for Effectiveness Outcome 40
Supplementary Table S8: Adjusted generalised linear mixed model for primary implementation outcome with clinic pairs as fixed effects 41
Supplementary Figure 24: Post-Hoc Pairwise Comparisons of Clinic Pairs for Implementation Outcome 42

Section 1: Supplementary Methods & Baseline clinic & Participants characteristics

Supplementary Methods 1

Study Design

In brief, BCPP was a multicomponent, multilevel household-based implementation science cluster randomised control trial designed to reduce HIV incidence across the 30 communities. The communities met the eligibility criteria if they were rural or peri-urban, with an average population of 6,000 people. Community pairs were assigned by random draw by people not involved in the BCPP trial. Pair-matched sites on HIV-related variables were maintained for InterCARE to understand the effects of integrating hypertension control and cardiovascular care on the established HIV outcomes, such as high rates of HIV viral suppression. None of the BCPP community pairs (or their clinics) closed down before InterCARE started.

Setting

Each had a catchment population of between 3,995 and 12,340 inhabitants (11). The prevalence of HIV across the 14 InterCARE communities was estimated to range from 25.7–33.3% at 5 sites [3 intervention], 21.7–25.5% at 1 site, 19.2–21.6% at 4 sites [3 intervention], 15.9–19.1% at 3 sites and 11.1–15.8% at 1 site [1 intervention]. In these communities, the participants were Black people primarily from the "Tswana" ethnic group of Botswana. In each community, the InterCARE study clinic was the main HIV clinic and was operated within the larger general primary care clinic. All clinics had access to electronic health record (EHR)- mostly PIMS. The PIMS© is a locally developed EHRs that was designed at the beginning of the Botswana national ARV roll-out program to document important program variables for HIV and transmit the variables to a national data warehouse (12). The PIMS©

has not been evaluated for the comprehensive delivery of integrated HIV services at HIV clinics in Botswana.

There were between 500 and 1,800 PWH who participated at each HIV clinic site. All the clinics had access to running water, electricity, computers with EHR software, and paper medical records. HIV care, including healthcare worker medical consultation, provision of antiretroviral therapy (ART), laboratory tests, and any other primary care services, such as those for hypertension, are provided for free.

Randomisation and masking

The paired sites were geographically distant to reduce contamination, with some pairs being 95 kilometres apart. Participant screening and recruitment occurred only once the selection of the BCPP pairs was complete.

Participants (treatment partners)

It was not mandatory to select a treatment partner; participants who opted to select a treatment partner were advised to select someone with whom they felt close and who had regular contact and who was willing to serve as a treatment partner. Treatment partners could be a relative, a friend, or a spouse/partner. After the start of the trial, we requested ethics approval to contact treatment partners via phone (which did not require them to visit the HIV clinic to make it easy for them to participate). Treatment partners could also be the participants' treatment supporters for HIV care. Treatment partners did not have to be living with HIV or hypertension. Chosen treatment partners could have been anyone whom the patient trusted, regardless of HIV or hypertension status, or enrolment in the study. Additionally, treatment partners did not have to live in the same community as the index patient; they just had to show that they had a supportive and trusting relationship, as reported by the participant (16).

Procedures

All study staff members completed training in good clinical practice (GCP) and received training on the protocol prior to the start of the trial. Study data (sociodemographic characteristics, comorbidities, medication use, cardiovascular risk assessment, and treatment partner interactions—to measure the delivery of support, such as frequency, type, and related activities) were collected at baseline and at 6, 12, 18, and 24 months and entered into RedCAP© (17). The study visit window after the baseline visit could have occurred as early as one month prior to or as late as 3 months after the scheduled study visit. Booster training sessions were provided to healthcare providers during site visits or virtually throughout the trial if the study team detected a decrease in fidelity to the InterCARE protocol. Trial activities and progress were reviewed weekly.

Secondary outcomes (RE-AIM)

These outcomes provide insights into the degree of potential public health and policy impact of this trial while also providing associated explanatory supporting data and narratives (mechanisms behind the impact achieved). A total of 12 secondary outcomes were assessed across all five RE-AIM domains and at various timepoints:

- **Reach**: i) proportion of PWH aware of their hypertension status; ii) proportion of PWH whose blood pressure is not controlled with antihypertensive medicine; iii) proportion of PWH with hypertension receiving antihypertensive medicines (all at 6, 12, 18 and 24 months).
- **Effectiveness**: i) decrease in systolic and diastolic blood pressure at 12 and 24 months; ii) proportion of PWH with >70% pass on the Heart Disease Fact Questionnaire (HDFQ) (19) at 6, 12, 18 and 24 months.
- **Adoption**: i) proportion of participants receiving HIV and hypertension care at the same clinic visit; ii) proportion of participants with 10-year cardiovascular risk calculated in the EHRs; iii) proportion of participants prescribed antihypertensive

medicine; iv) proportion of participants with waist and hip circumference recorded in the EHRs (all measured at 6, 12, 18 and 24 months).

- **Implementation Fidelity**: i) Proportion of PWH and HTN on correct antihypertensive regimens as per local guidelines at 6, 12, 18 and 24 months.
- **Maintenance**: i) proportion of intervention clinics that implemented all 3 InterCARE strategies at 24 months; ii) proportion of PWH with hypertension enrolled in InterCARE with BP control (at baseline) who maintain BP control at subsequent visits (6, 12, 18 and 24 months).

Consistent with the time of ascertaining the prespecified trial outcomes, teams of trained research assistants and registered nurses based at each trial site obtained trial-related data from patients and clinic records (e.g., EHRs) at baseline and at 6, 12, 18 and 24 months following randomisation. Data were entered into the trial data collection form on RedCap© (17). A dedicated data quality monitoring team, which was separate from the team that collected data at the trial sites, provided daily feedback to the trial site teams on data quality and completeness for improvement and correction.

Statistical analysis

Our main sample size calculations for the primary ITT populations were based on each of two independent effectiveness and implementation coprimary outcomes. Our primary effectiveness outcomes were restricted to those on antihypertensive medicines. Based on a recent HIV incidence cluster randomised control trial in Botswana (3), we assumed an unequal number of participants given the variability in the HIV clinic population size in each community and a within-clinic correlation coefficient of 0.055. Additionally, assuming 10% refusal rate, a 10% loss to follow-up at 12 months, and a two-sided 95% confidence interval, then we would need to enrol a total N=2,338 participants on antihypertensive medicines (167 per HIV clinic) to achieve a valid sample size of 1872 participants with a primary endpoint measurement, across 7 interventions versus 7 standard-of-care HIV clinics, in order to have 80% power to detect a minimum difference of 18.3% in the proportion with blood pressure control and 14.9% in the uptake of electronic prescription of antihypertensive medicines. This assumes 41.7% blood pressure control and 14% e-prescriptions to the standard-of-care group at the 12-month study visit.

For the secondary ITT (all participants irrespective whether they were on antihypertensive or not), sample and power calculations were calculated for the primary effectiveness outcomes only because primary implementation outcome (prescribing of antihypertensive medicines on EHR) was not relevant for this population. Therefore, considering 50% of participants will be on antihypertensive medicines, a 10% refusal rate, a 10% loss to follow-up at 12 months, and a two-sided 95% confidence interval, the trial had to enrol an estimated 2,331 participants in the intervention group and 2,331 participants in the standard-of-care group (total N=4,662, 333 per HIV clinic, to achieve a valid sample size of 3730 participants with a primary endpoint measurements) across 7 interventions versus 7 standard-of-care HIV clinics to detect at least a 15.8% (assuming 19% blood pressure control in the standard-of-care) difference in the primary effectiveness outcome. More information on sample size determination can be found in the protocol paper [7]. No alpha adjustment was made in the sample size calculation or primary outcome analyses.

In post hoc sensitivity analysis, we assessed the effect of cluster-level characteristics and the imbalance between study arms. Finally, we also reran adjusted GLMM with clinic pairs as fixed effects plus post-hoc pairwise comparisons of clinic pairs for both primary outcomes.

Supplementary Figure 1: Full InterCARE consort diagram

EHR: Electronic Health Record System

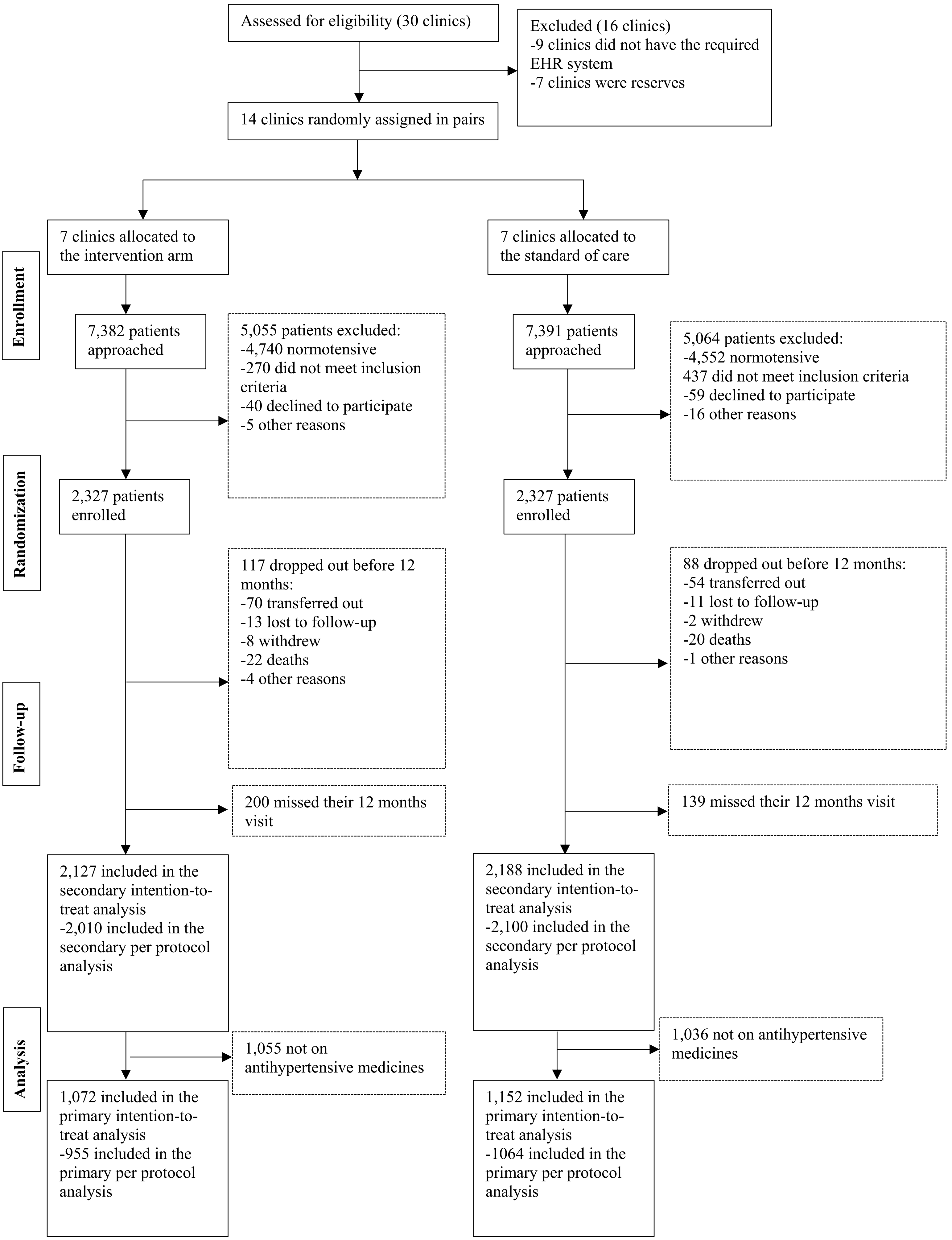

Supplementary Table S1: Pair allocation to intervention versus standard-of care for parent trial (BCPP) and current trial (InterCARE)

**Pairs**	**BCPP**	**InterCARE**
Pair 1	Intervention	Intervention
	SOC	SOC
Pair 2	Intervention	Intervention
	SOC	SOC
Pair 3	Intervention	Intervention
	SOC	SOC
Pair 4	Intervention	Intervention
	SOC	SOC
Pair 5	SOC	Intervention
	Intervention	SOC
Pair 6	Intervention	SOC
	SOC	Intervention
Pair 7	Intervention	Intervention
	SOC	SOC

Abbreviations: SOC: Standard of Care

Supplementary Table S2: Additional baseline characteristics of the study participants

	Primary intention-to-treat population			Secondary intention-to-treat population		
	All (N = 2,224)	Intervention (N = 1,072)	Standard of care (N = 1,152)	All (N = 4,315)	Intervention (N = 2,127)	Standard of care (N = 2,188)
**Socio-demographics**						
**Sex, N (%)**						
Female	1,535 (69.0%)	740 (69.0%)	795 (69.0%)	2,651 (61.4%)	1,290 (60.6%)	1,361 (62.2%)
Male	689 (31.0%)	332 (31.0%)	357 (31.0%)	1,664 (38.6%)	837 (39.4%)	827 (37.8%)
**Age, Mean (SD)**	54.0 (10.3)	53.5 (10.5)	54.4 (10.1	51.3 (11.0)	50.7 (11.1)	51.8 (10.8)
**Marital Status, N (%)**						
Single	1,435 (64.5%)	720 (67.2%)	715 (62.1%)	3,084 (71.5%)	1,548 (72.8%)	1,536 (70.2%)
Married	543 (24.4%)	246 (22.9%)	297 (25.8%)	903 (20.9%)	436 (20.5%)	467 (21.3%)
Divorced/Separated	72 (3.2%)	32 (3.0%)	40 (3.5%)	94 (2.2%)	41 (1.9%)	53 (2.4%)
Widowed	174 (7.8%)	74 (6.9%)	100 (8.7%)	234 (5.4%)	102 (4.8%)	132 (6.0%)
**Employment, N (%)**						
Employed	782 (35.2%)	361 (33.7%)	421 (36.5%)	1,751 (40.6%)	840 (39.5%)	911 (41.6%)
Not employed	1,442 (64.8%)	711 (66.3%)	731 (63.5%)	2,564 (59.4%)	1,287 (60.5%)	1,277 (58.4%)
**Main job, N (%)**						
Casual	156 (19.9%)	75 (20.8%)	81 (19.2%)	334 (19.1%)	168 (20.0%)	166 (18.2%)
Seasonal	44 (5.6%)	22 (6.1%)	22 (5.2%)	102 (5.8%)	61 (7.3%)	41 (4.5%)
Formal (Part time)	45 (5.8%)	11 (3.0%)	34 (8.1%)	98 (5.6%)	28 (3.3%)	70 (7.7%)
Formal (Full time)	312 (39.9%)	134 (37.1%)	178 (42.3%)	690 (39.4%)	296 (35.2%)	394 (43.2%)
Self-employed	217 (27.7%)	118 (32.7%)	99 (23.5%)	511 (29.2%)	286 (34.0%)	225 (24.7%)
Other	8 (1.0%)	1 (0.3%)	7 (1.7%)	16 (0.9%)	1 (0.1%)	15 (1.6%)
***Monthly income, N (%)**						
No income	494 (22.2%)	233 (21.7%)	261 (22.7%)	957 (22.2%)	464 (21.8%)	493 (22.5%)
Less than $6.74	884 (39.7%)	466 (43.5%)	418 (36.3%)	1,555 (36.0%)	822 (38.6%)	733 (33.5%)
$6.74 – $348.62	697 (31.3%)	314 (29.3%)	383 (33.2%)	1,484 (34.4%)	693 (32.6%)	791 (36.2%)
$348.69 or more	148 (6.7%)	59 (5.5%)	89 (7.7%)	316 (7.3%)	146 (6.9%)	170 (7.8%)
Declined to answer	1 (0.0%)	0 (0.0%)	1 (0.1%)	3 (0.1%)	2 (0.1%)	1 (0.0%)
**Aware of HTN, N (%)**						
Yes	2,194 (98.7%)	1,063 (99.2%)	1,131 (98.2%)	3,978 (92.2%)	2,101 (98.8%)	1,877 (85.8%)
No	30 (1.3%)	9 (0.8%)	21 (1.8%)	337 (7.8%)	26 (1.2%)	311 (14.2%)
**Duration of HTN diagnosis in years, Median (IQR)**	4.90 (1.14 – 10.7)	3.86 (0.62 – 9.83)	5.89 (1.90 – 11.4)	1.24 (0.17 - 6.39)	0.91 (0.00 - 4.99)	1.97 (0.41 - 7.96)
**On HTN medications**						

Yes	1,835 (82.5%)	817 (76.2%)	1,018 (88.4%)	1,895 (43.9%)	834 (39.2%)	1,061 (48.5%)
No	389 (17.5%)	255 (23.8%)	134 (11.6%)	2,420 (56.1%)	1,293 (60.8%)	1,127 (51.5%)
**Duration of HTN meds in years, Median (IQR)**	6.04 (2.23 – 11.9)	5.41 (2.10 – 11.7)	6.40 (2.74 – 12.0)	5.92 (2.15 - 11.71)	5.33 (2.04 - 11.67)	6.14 (2.39 - 11.82)
**No. of antihypertensives, Median (IQR)**	2.0 (1.0 – 2.0)	1.0 (1.0 – 2.0)	2.0 (1.0 – 2.0)	2.00 (1.00 – 2.00)	1.00 (1.00 – 2.00)	2.00 (1.00 – 2.00)
**BP drug classes, N (%)**						
Thiazide	1,266 (69.0%)	541 (66.2%)	725 (71.2%)	1,303 (68.8%)	550 (65.9%)	53 (71.0%)
CCB	899 (49.0%)	406 (49.7%)	493 (48.4%)	918 (48.4%)	413 (49.5%)	05 (47.6%)
ACEi/ARB	360 (19.6%)	151 (18.5%)	209 (20.5%)	370 (19.5%)	155 (18.6%)	15 (20.3%)
Beta-Blockers	184 (10.0%)	83 (10.2%)	101 (9.9%)	187 (9.9%)	85 (10.2%)	102 (9.6%)
Alpha-Blockers	5 (0.3%)	1 (0.1%)	4 (0.4%)	5 (0.3%)	1 (0.1%)	4 (0.4%)
Others	171 (9.3%)	91 (11.1%)	80 (7.9%)	177 (9.3%)	92 (11.0%)	85 (8.0%)
**No. of prescribed classes, N (%)**						
One drug class	944/1835 (51.4%)	432/817 (52.9%)	512/1018 (50.3%)	989/1895 (52.2%)	443/834 (53.1%)	546/1061 (51.5%)
Two drug classes	748/1835 (40.8%)	322/817 (39.4%)	426/1018 (41.8%)	763/1895 (40.3%)	328/834 (39.3%)	435/1061 (41.0%)
Three drug classes	132/1835 (7.2%)	56/817 (6.9%)	76/1018 (7.5%)	132/1895 (7.0%)	56/834 (6.7%)	76/1061 (7.2%)
Four or more drug classes	11/1835 (0.6%)	7/817 (0.9%)	4/1018 (0.4%)	11/1895 (0.6%)	7/834 (0.8%)	4/1061 (0.4%)
**BP meds in PIMS, N (%)**						
Yes	256 (14.0%)	133 (16.3%)	123 (12.1%)	265 (14.0%)	136 (16.3%)	129 (12.2%)
No	1,579 (86.0%)	684 (83.7%)	895 (87.9%)	1,630 (86.0%)	698 (83.7%)	932 (87.8%)
**HIV duration in years, Median (IQR)**	11.8 (7.28 – 16.5)	10.8 (6.90 – 15.5)	12.6 (8.05 – 17.1)	10.8 (6.88 - 15.4)	10.3 (6.65 - 15.0)	11.3 (7.15 - 16.0)
**Viral suppression, N (%)**						
Yes, ≤ 400	2,009 (90.3%)	947 (88.3%)	1,062 (92.2%)	3,872 (89.7%)	1,845 (86.7%)	2,027 (92.6%)
No	20 (0.9%)	14 (1.3%)	6 (0.5%)	41 (1.0%)	23 (1.1%)	18 (0.8%)
Missing data	195 (8.8%)	111 (10.4%)	84 (7.3%)	402 (9.3%)	259 (12.2%)	143 (6.5%)
Current CD4 Count (cell/uL), Median (IQR)	673.0 (483.5 – 872.5)	641.0 (443.5 – 853.0)	702.5 (519.0 – 899.0)	633.0 (458.5 – 841.0)	604.0 (428.0 – 816.0)	659.5 (479.0 – 857.0)
Right SBP, mmHg	136.0 (125.0 – 147.0)	136.0 (125.0 – 146.0)	136.0 (124.0 – 148.0)	140.0 (129.0 – 147.0)	139.0 (130.0 – 145.0)	141.0 (129.0 – 150.0)
Right DBP, mmHg	84.0 (76.0 – 91.0)	83.0 (76.0 – 90.0)	84.0 (76.0 – 91.0)	85.0 (78.0 – 91.0)	84.0 (78.0 – 90.0)	87.0 (79.0 – 92.0)
Left SBP, mmHg	136.0 (125.0 – 147.0)	135.5 (124.0 – 145.0)	136.0 (125.0 – 148.0)	140.0 (130.0 – 147.0)	139.0 (130.0 – 145.0)	141.0 (129.0 – 149.0)
Left DBP, mmHg	83.0 (76.0 – 91.0)	82.0 (76.0 – 90.0)	84.0 (76.0 – 91.0)	85.0 (79.0 – 91.0)	84.0 (78.0 – 90.0)	87.0 (79.0 – 92.0)

Waist circumference in cm, Median (IQR)	89.5 (81.0 – 99.1)	88.0 (78.0 – 96.0)	92.0 (83.0 – 101.6)	88.0 (79.0 – 97.0)	86.0 (77.0 – 95.0)	90.0 (81.3 - 99.1)
Hip circumference in cm, Median (IQR)	100.0 (91.4 – 110.0)	100.0 (91.0 – 109.0)	100.8 (91.6 – 111.8)	98.0 (90.0 - 108.5)	98.0 (89.5 – 107.0)	99.0 (90.0 - 109.2)
**Tobacco use, N (%)**						
Yes	216 (9.7%)	107 (10.0%)	109 (9.5%)	551 (12.8%)	273 (12.8%)	278 (12.7%)
No	2,008 (90.3%)	965 (90.0%)	1,043 (90.5%)	3,764 (87.2%)	1,854 (87.2%)	1,910 (87.3%)
**Consumption of tobacco, N (%)**						
Less than 1 week	16 (7.4%)	8 (7.5%)	8 (7.3%)	32 (5.8%)	14 (5.1%)	18 (6.5%)
Once a week	10 (4.6%)	6 (5.6%)	4 (3.7%)	17 (3.1%)	12 (4.4%)	5 (1.8%)
2 to 3 times a week	42 (19.4%)	25 (23.4%)	17 (15.6%)	129 (23.4%)	70 (25.6%)	59 (21.2%)
More than 3 times a week	140 (64.8%)	68 (63.6%)	72 (66.1%)	357 (64.8%)	177 (64.8%)	180 (64.7%)
Don't want to answer	8 (3.7%)	0 (0.0%)	8 (7.3%)	16 (2.9%)	0 (0.0%)	16 (5.8%)
**Type of tobacco use, N (%)**						
Snuff	84 (38.9%)	35 (32.7%)	49 (45.0%)	156 (28.3%)	59 (21.6%)	97 (34.9%)
Cigarettes	133 (61.6%)	73 (68.2%)	60 (55.0%)	393 (71.3%)	216 (79.1%)	177 (63.7%)
Cigars	1 (0.5%)	0 (0.0%)	1 (0.9%)	4 (1.4%)	1 (0.4%)	5 (0.9%)
Others	1 (0.5%)	0 (0.0%)	1 (0.9%)	5 (1.8%)	1 (0.4%)	6 (1.1%)
**Alcohol use, N (%)**						
Yes	336 (15.1%)	166 (15.5%)	170 (14.8%)	869 (20.1%)	423 (19.9%)	446 (20.4%)
No	1,888 (84.9%)	906 (84.5%)	982 (85.2%)	3,446 (79.9%)	1,704 (80.1%)	1,742 (79.6%)
**Consumption of alcohol, N (%)**						
Less than once a week	92 (27.4%)	40 (24.1%)	52 (30.6%)	236 (27.2%)	110 (26.0%)	126 (28.3%)
Once a week	112 (33.3%)	62 (37.3%)	50 (29.4%)	257 (29.6%)	137 (32.4%)	120 (26.9%)
2 to 3 times a week	72 (21.4%)	38 (22.9%)	34 (20.0%)	204 (23.5%)	103 (24.3%)	101 (22.6%)
More than 3 times a week	53 (15.8%)	25 (15.1%)	28 (16.5%)	148 (17.0%)	68 (16.1%)	80 (17.9%)
Don't want to answer	7 (2.1%)	1 (0.6%)	6 (3.5%)	24 (2.8%)	5 (1.2%)	19 (4.3%)
**CVD risk estimation in PIMS, N (%)**						
Yes	106 (4.8%)	101 (9.4%)	5 (0.4%)	181 (4.2%)	172 (8.1%)	9 (0.4%)
No	2,115 (95.1%)	970 (90.5%)	1,145 (99.4%)	4,127 (95.6%)	1,952 (91.8%)	2,175 (99.4%)
Missing	3 (0.1%)	1 (0.1%)	2 (0.2%)	7 (0.2%)	3 (0.1%)	4 (0.2%)
**Controlled BP, N (%)**						
Yes	1,121 (50.4%)	554 (51.7%)	567 (49.2%)	1,765 (40.9%)	960 (45.1%)	805 (36.8%)
No	1,103 (49.6%)	518 (48.3%)	585 (50.8%)	2,550 (59.1%)	1,167 (54.9%)	1,383 (63.2%)

Abbreviations:
SD: Standard deviation; IQR: Interquartile range; HTN: Hypertension; BP: Blood pressure; SBP / DBP: Systolic / Diastolic blood pressure; CCB: Calcium channel blocker; ACEi: Angiotensin-converting enzyme inhibitor; ARB: Angiotensin receptor blocker; PIMS: Performance Information Management System (electronic health record); CVD: Cardiovascular disease

Section 2: Secondary outcomes for the Primary Intention-to-treat (ITT) population

Supplementary Figure 2: Proportion of participants aware of their hypertension status at 6, 12, 18, 24 months study visit (REACH) (Primary intention-to-treat population)

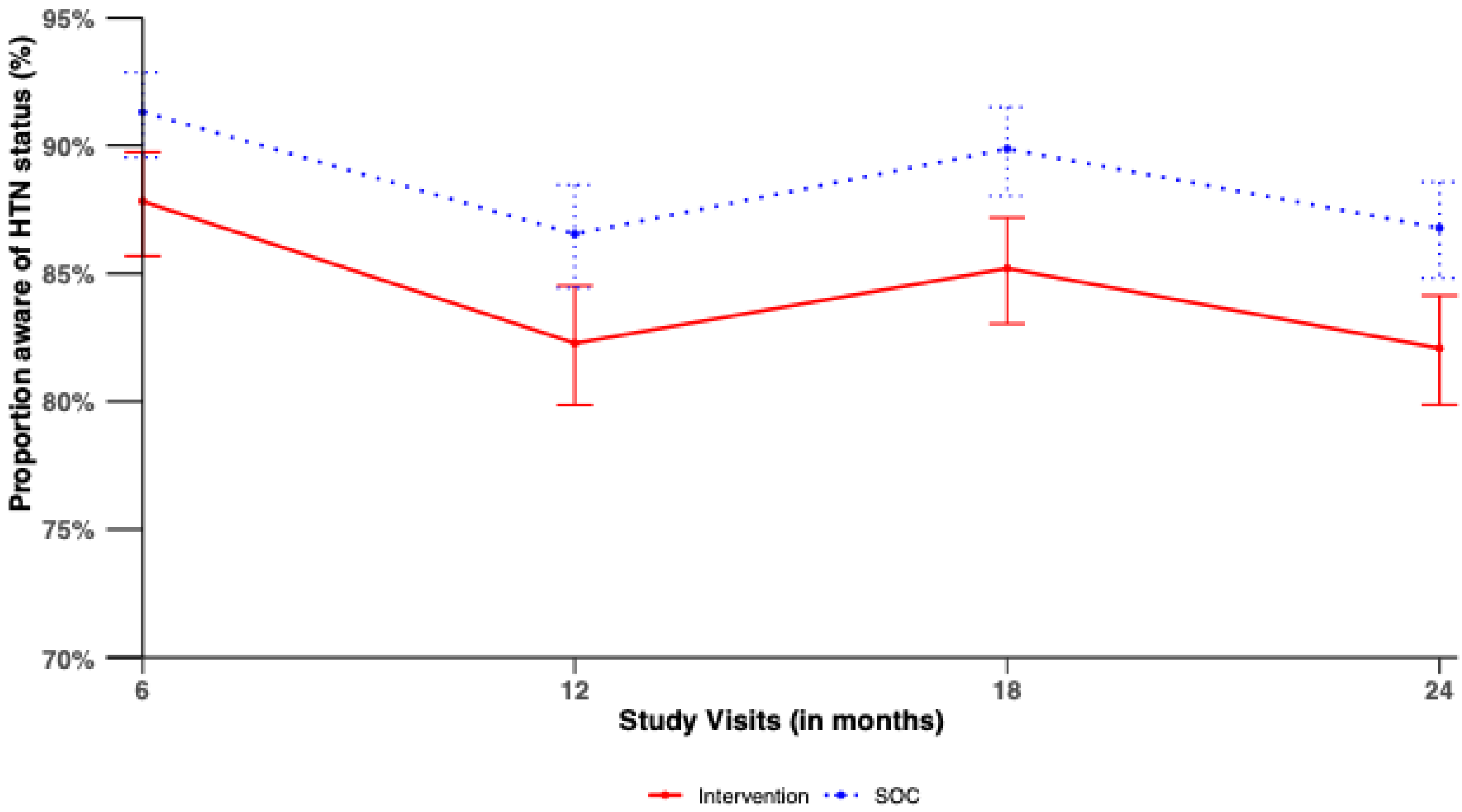

Study Visits	Intervention n/N (%)	Standard-of-care n/N (%)	Unadjusted Risk Ratio (95% CI)	P value
6 months	916/1,043 (87.8%)	1,062/1,163 (91.3%)	0.98 (0.90 – 1.07)	0.64
12 months	882/1,072 (82.3%)	997/1,152 (86.5%)	0.96 (0.87 – 1.05)	0.34
18 months	996/1,169 (85.2%)	1,083/1,205 (89.9%)	0.95 (0.87 – 1.04)	0.26
24 months	1,049/1,278 (82.1%)	1,136/1,309 (86.8%)	0.95 (0.87 – 1.03)	0.19

Abbreviations: HTN: Hypertension; SOC: Standard-of-care

Supplementary Figure 3: Decrease in mean systolic and diastolic blood pressures at 6, 12, 18 and 24 months visit relative to the baseline values (EFFECTIVENESS) (Primary intention-to-treat population)

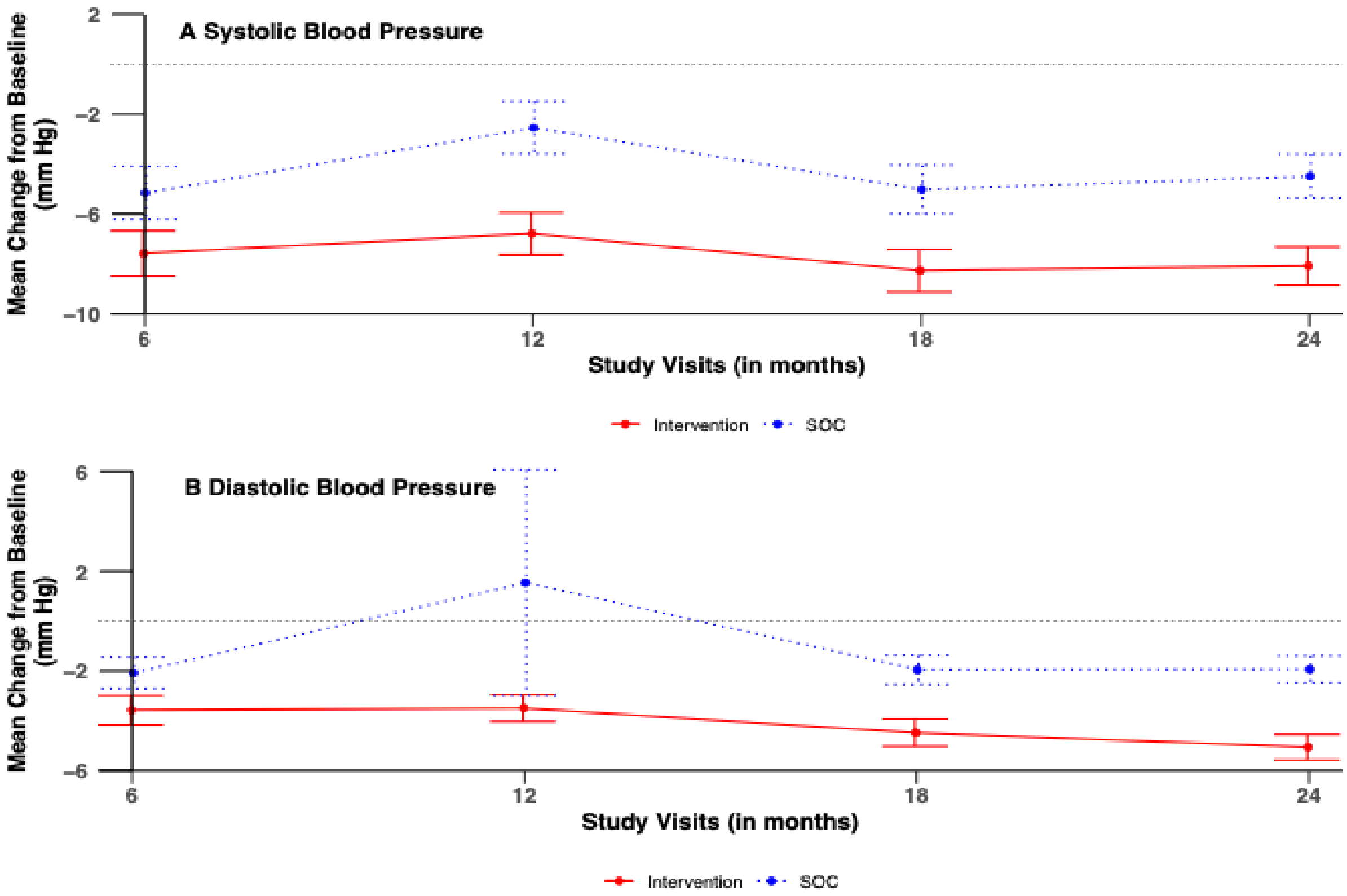

Study Visit	6 Months		12 Months		18 Months		24 Months	
	Intervention (N=1,043)	SOC (N=1,163)	Intervention (N=1,072)	SOC (N=1,152)	Intervention (N=1,169)	SOC (N=1,205)	Intervention (N=1,278)	SOC (N=1,309)
Systolic Mean change (SD)	-7.6(14.6)	-5.2(18.3)	-6.8(13.9)	-2.5(17.8)	-8.3(14.8)	-5.0 (17.3)	-8.1 (14.1)	-4.5 (16.4)
Systolic Mean (SD)	122 (34.4)	128 (31.4)	115 (43.7)	124 (40.7)	110 (47.2)	120 (42.7)	106 (50.7)	116 (47.4)
Mean diff (95%CI)	-4.18 (-11.6, 3.29)		-7.98 (-17.9, 1.96)		-8.23 (-18.9, 2.41)		-9.41 (-19.8, 0.94)	
P value	0.25		0.106		0.12		0.071	
Diastolic mean change (SD)	-3.6(9.4)	-2.1 (10.9)	-3.5 (8.7)	1.5 (76.7)	-4.5(9.6)	-2.0 (10.5)	-5.1(9.4)	-1.9 (10.1)
Diastolic Mean (SD)	75.3 (21.4)	78.7 (19.2)	70.9 (26.9)	78.9 (80.2)	67.5(29.2)	74.0 (26.3)	64.1(31.0)	71.2 (29.2)
Mean diff (95%CI)	-2.54 (-8.01, 2.94)		-7.55 (-16.30, 1.22)		-5.22 (-12.1, 1.66)		-6.53 (-13.8, 0.75)	
P value	0.33		0.085		0.13		0.074	

Abbreviations: Hg: mercury; MM: millimetres; SOC: Standard-of-care

Supplementary Figure 4: Proportion of participants receiving HIV and hypertension care at same clinic during 6, 12, 18, 24 months visit (Adoption) (Primary intention-to-treat population)

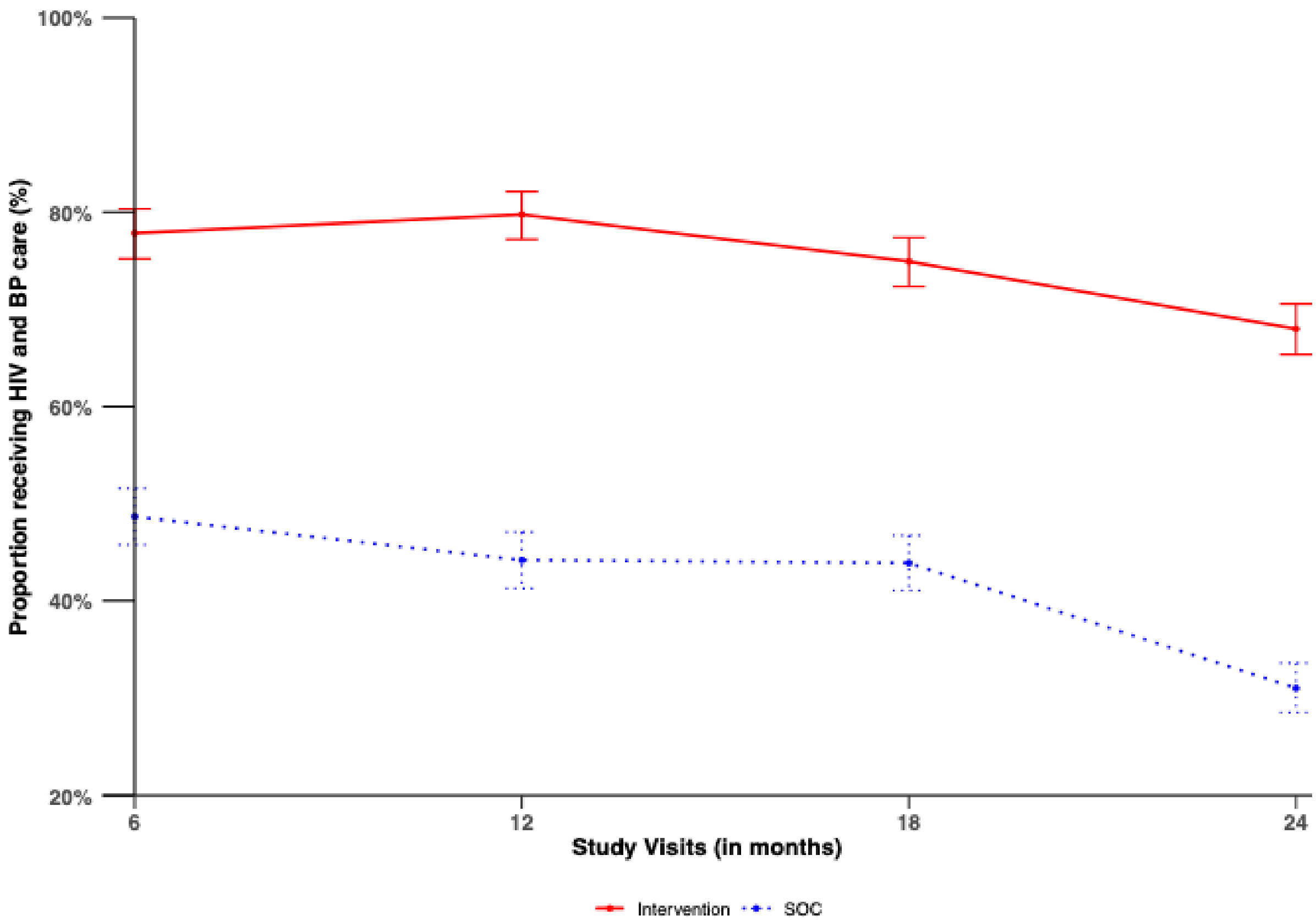

Study Visits	Intervention n/N (%)	Standard-of-care n/N (%)	Unadjusted Risk Ratio (95% CI)	P value
6 months	812/1,043 (77.9%)	566/1,163 (48.7%)	1.76 (1.04 – 2.95)	0.034
12 months	855/1,072 (79.8%)	509/1,152 (44.2%)	1.99 (1.36 – 2.93)	<0.0001
18 months	876/1,169 (74.9%)	529/1,205 (43.9%)	2.87 (1.16 – 7.11)	0.023
24 months	869/1,278 (68.0%)	406/1,309 (31.0%)	3.76 (1.45 – 9.77)	0.0066

Abbreviations: BP: Blood pressure; HIV: Human immunodeficiency virus; SOC: Standard-of-care

Supplementary Figure 5: Proportion of participants with >70% pass on Heart Disease Fact Questionnaire (HDFQ) during the 6, 12, 18 and 24 months visit (EFFECTIVENESS) (Primary intention-to-treat population)

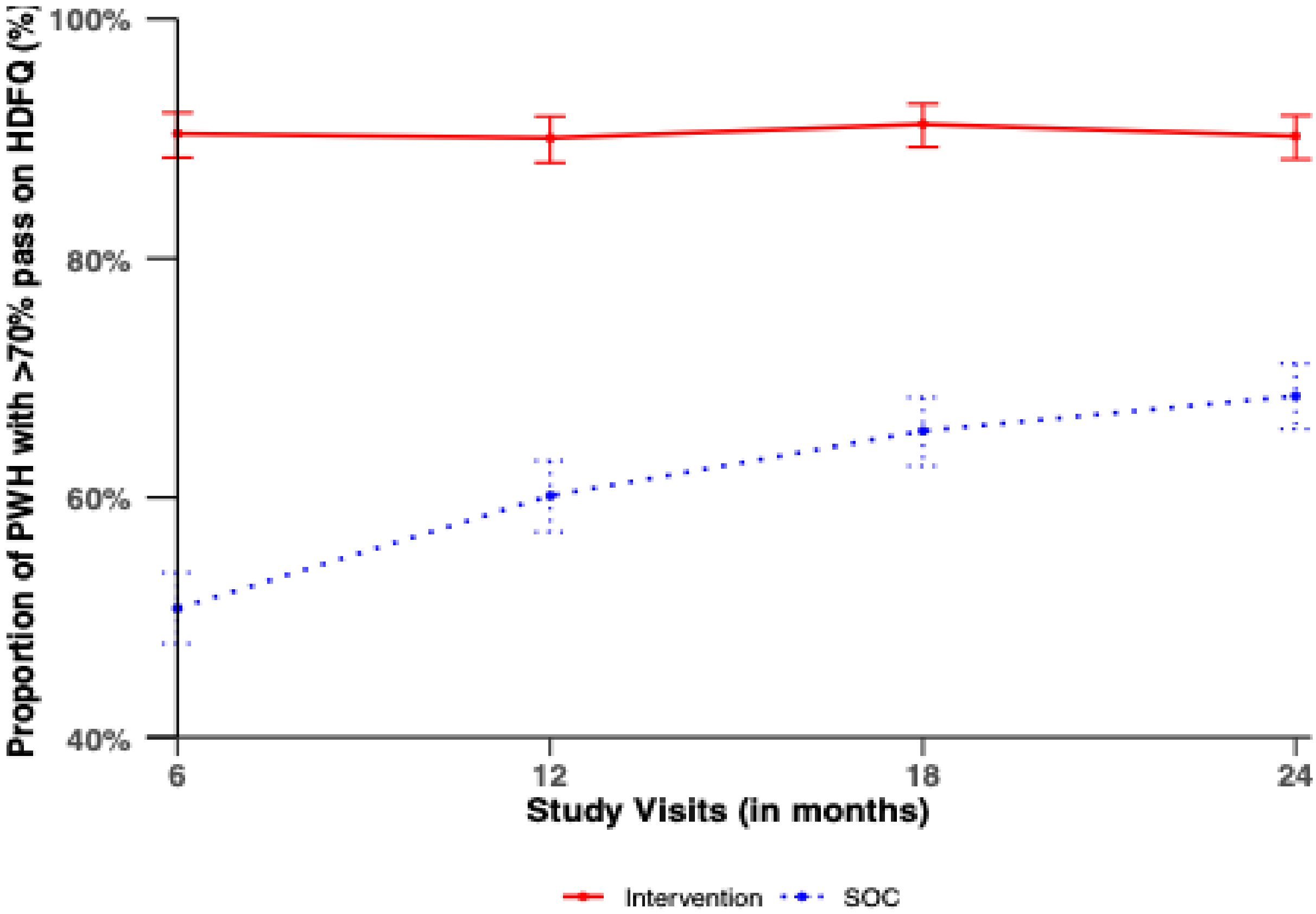

Study Visits	Intervention n/N (%)	Standard-of-care n/N (%)	Unadjusted Risk Ratio (95% CI)	P value
6 months	887/1,043 (85.0%)	568/1,163 (48.8%)	2.37 (1.11 – 5.06)	0.025
12 months	860/1,072 (80.2%)	640/1,152 (55.6%)	1.41 (0.93 – 2.15)	0.11
18 months	914/1,169 (78.2%)	714/1,205 (59.3%)	1.21 (0.91– 1.60)	0.19
24 months	950/1,278 (74.3%)	781/1,309 (59.7%)	1.16(1.04 – 1.28)	0.0048

Abbreviations: HDFQ: Heart Disease Fact Questionnaire; PWH: People living with HIV; SOC: Standard-of-care

Supplementary Figure 6: Proportion of the participants with controlled blood pressure at baseline who maintain blood pressure control in next study visit: (MAINTENANCE) (Primary intention-to-treat population)

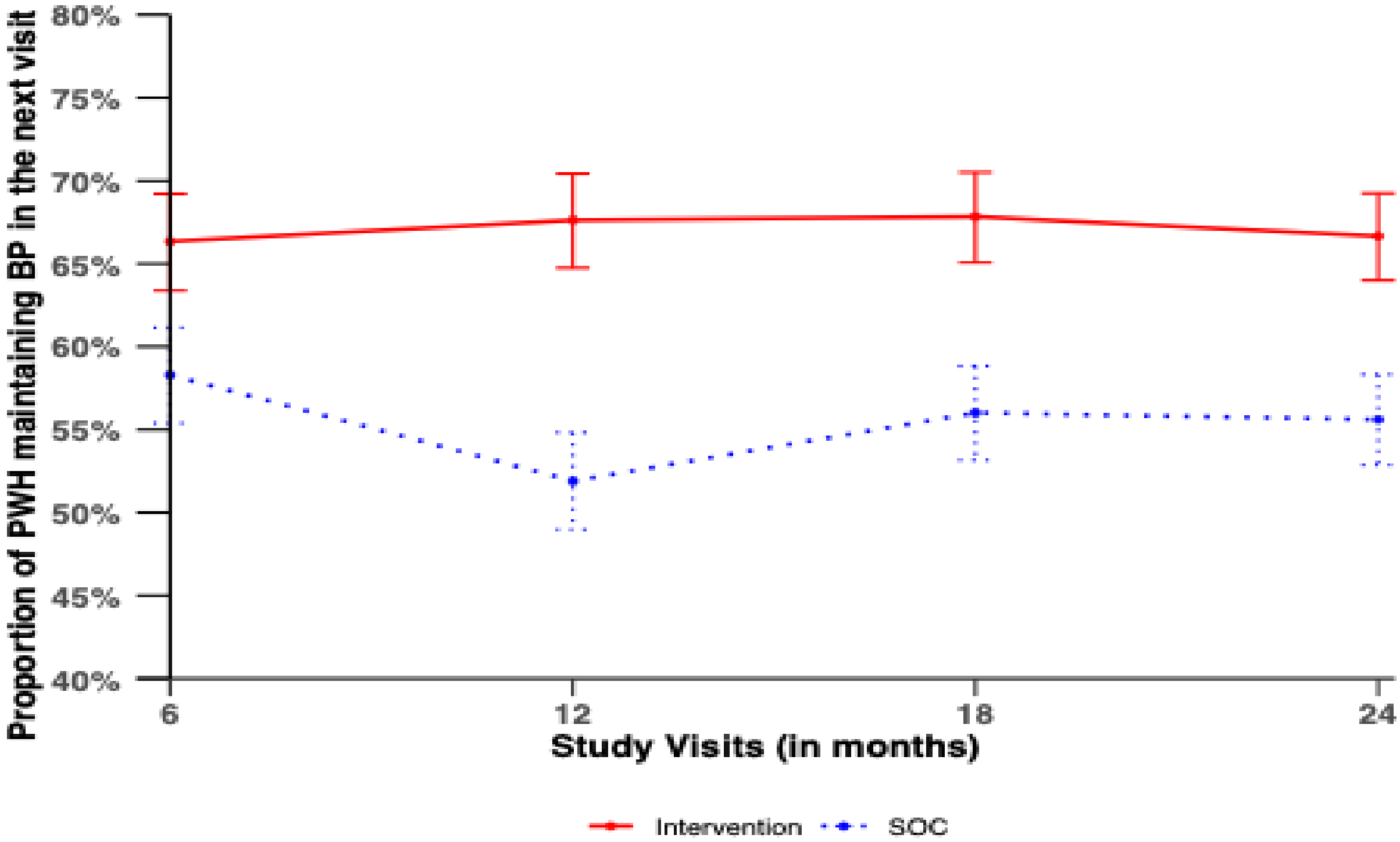

Study Visits	Intervention n/N (%)	Standard-of-care n/N (%)	Unadjusted Risk Ratio (95% CI)	P value
6 months	692/1,043 (66.3%)	678/1,163 (58.3%)	1.18 (1.05 – 1.32)	0.0040
12 months	725/1,072 (67.6%)	598/1,152 (51.9%)	1.29 (1.10 – 1.51)	0.0013
18 months	793/1,169 (67.8%)	675/1,205 (56.0%)	1.23 (1.07– 1.41)	0.0033
24 months	852/1,278 (66.7%)	728/1,309 (55.6%)	1.21 (1.09 – 1.24)	0.0003

Abbreviations: BP: Blood pressure; PWH: People living with HIV; SOC: Standard-of-care

Supplementary Figure 7: Proportion of the participants with 10-year cardiovascular risk calculated in the electronic health records (Primary intention-to-treat population)

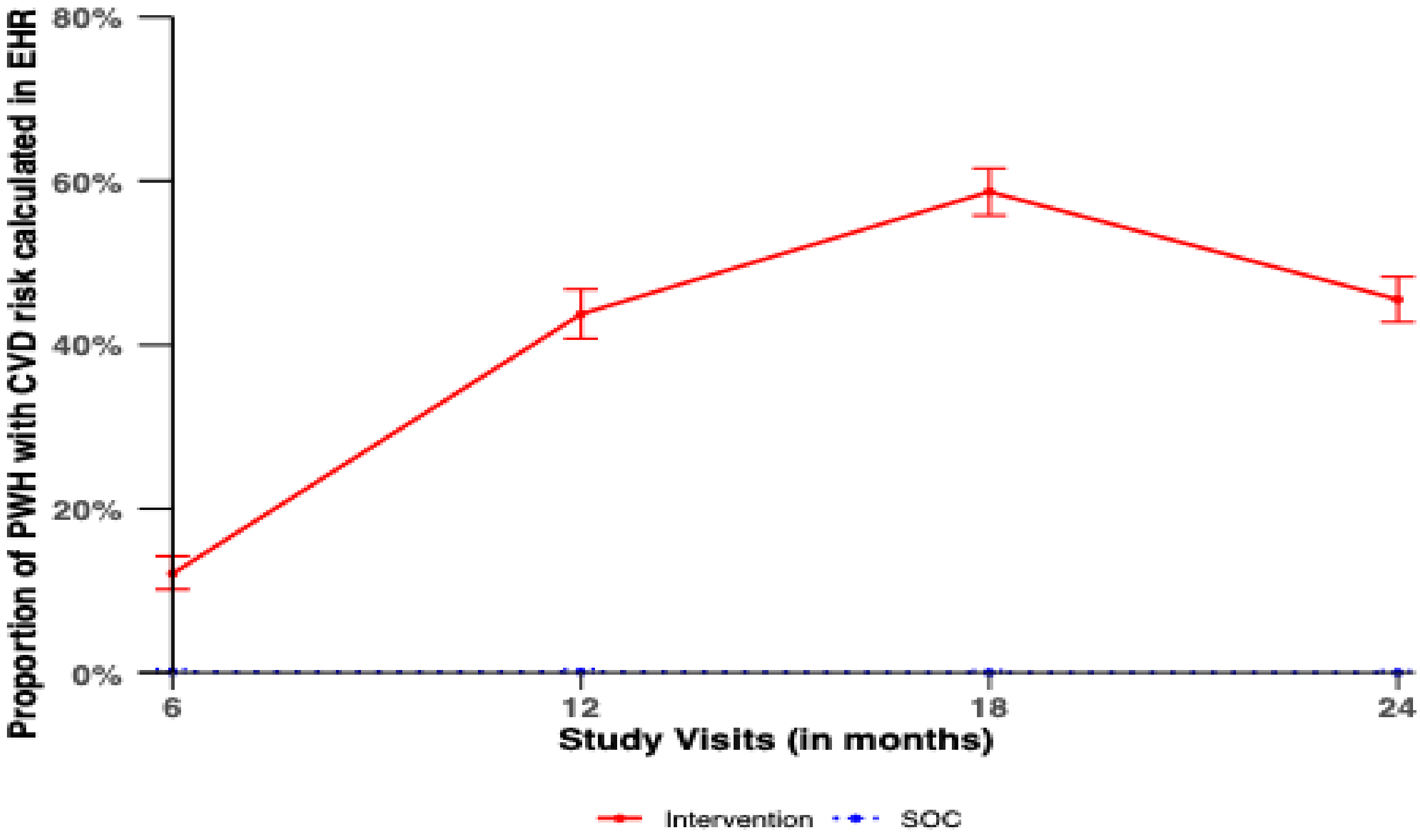

Study Visits	Intervention n/N (%)	Standard-of-care n/N (%)	Unadjusted Risk Ratio (95% CI)	P value
6 months	126/1,043 (12.1%)	1/1,163 (0.1%)	153.41 (21.40 – 1099.68)	<0.0001
12 months	469/1,072 (43.8%)	1/1,152 (0.1%)	437.96 (61.49 – 3119.16)	<0.0001
18 months	686/1,169 (58.7%)	0/1,205 (0.0%)	-	-
24 months	582/1,278 (45.5%)	0/1,309 (0.0%)	-	-

Abbreviations: CVD: Cardiovascular diseases; EHR: Electronic health record; PWH: People living with HI; SOC: Standard-of-care

Supplementary Figure 8: Proportion of the participants on medications with correct regimen per national guidelines (Primary intention-to-treat population)

Guideline concordance defined as CCB or HCTZ for monotherapy, CCB/HCTZ combo or with ACEI/ARB for dual therapy, and then CCB/HCTZ/ACEI/ARB for triple therapy, and finally, CCB/HCTZ/ACEI/ARB plus alpha/Beta-blocker

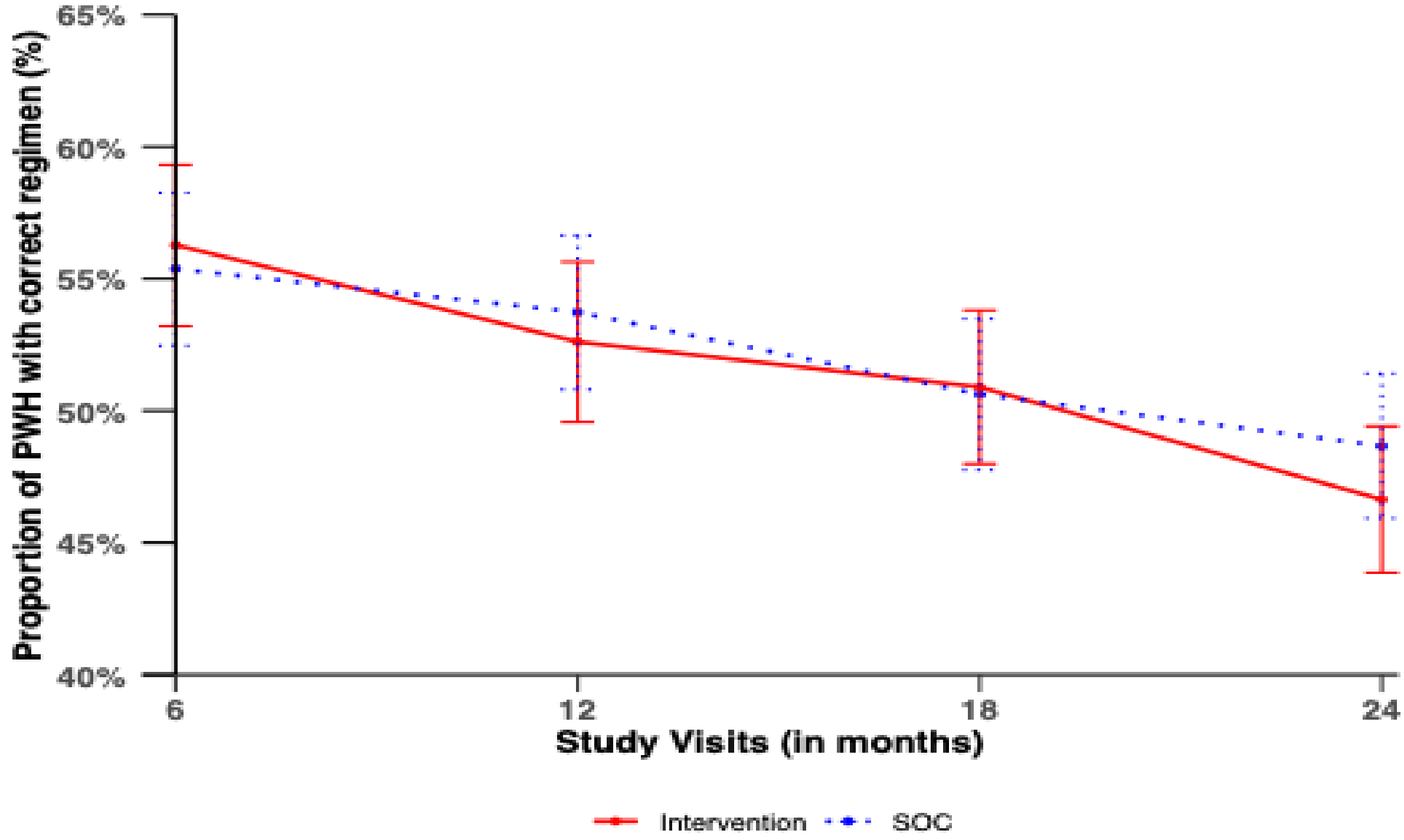

Study Visits	Intervention n/N (%)	Standard-of-care n/N (%)	Unadjusted Risk Ratio (95% CI)	P value
6 months	587/1,043 (56.3%)	644/1,163 (55.4%)	1.04 (0.91 – 1.20)	0.57
12 months	564/1,072 (52.6%)	619/1,152 (53.7%)	0.99 (0.86 – 1.14)	0.86
18 months	595/1,169 (50.9%)	610/1,205 (50.6%)	1.02 (0.87 – 1.19)	0.82
24 months	596/1,278 (46.6%)	637/1,309 (48.7%)	0.96 (0.81 – 1.13)	0.64

Abbreviations:PWH: People living with HIV; SOC: Standard-of-care

Supplementary Figure 9: Proportion of the participants with waist and hip circumference recorded in Electronic Health Records (Primary intention-to-treat population)

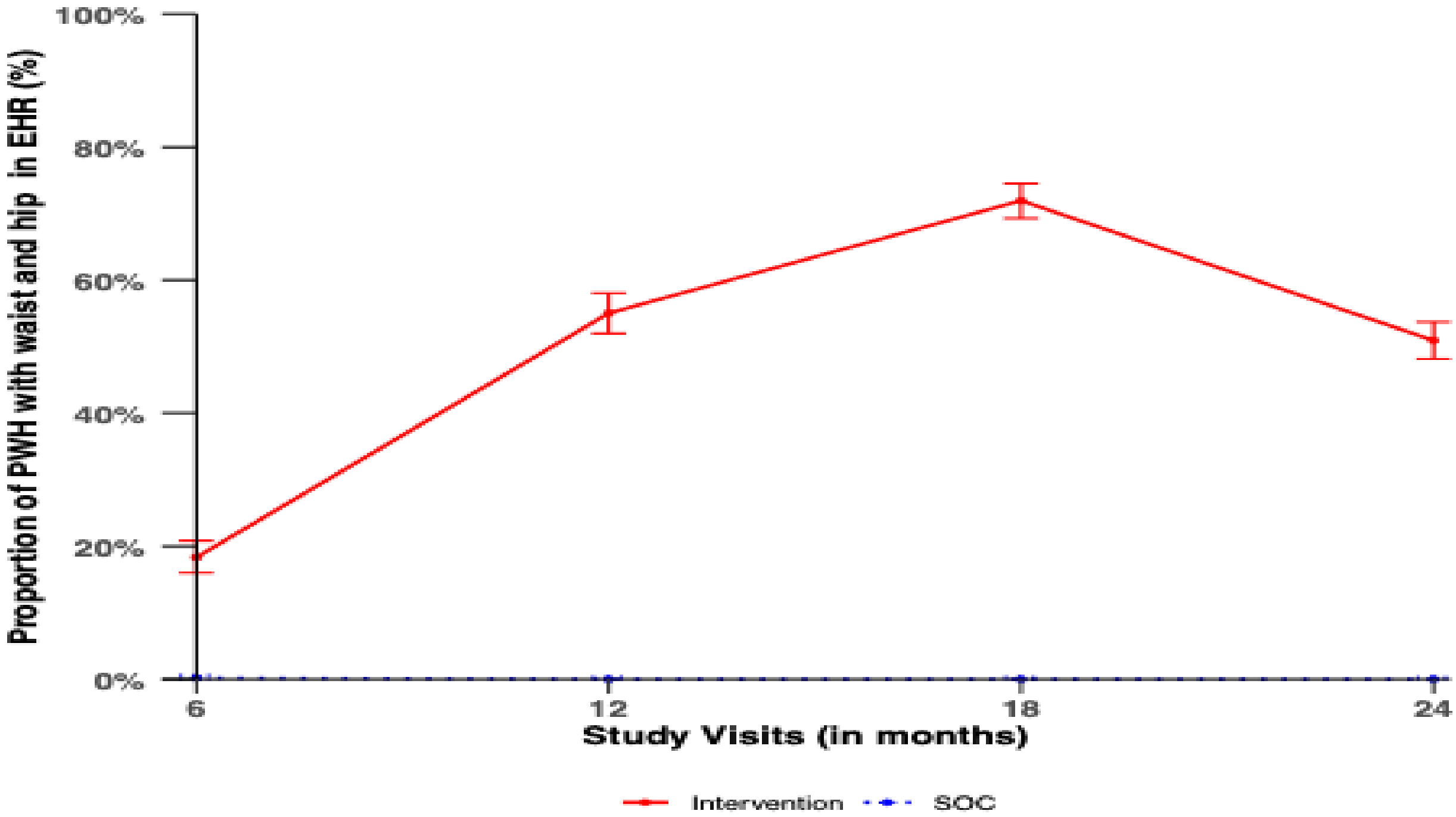

Study Visits	Intervention n/N (%)	Standard-of-care n/N (%)	Unadjusted Risk Ratio (95% CI)	P value
6 months	191/1,043 (18.3%)	2/1,163 (0.2%)	111.79 (27.70 – 451.14)	<0.0001
12 months	590/1,072 (55.0%)	0/1,152 (0.0%)	-	-
18 months	841/1,169 (71.9%)	0/1,152 (0.0%)	-	-
24 months	651/1,278 (50.9%)	0/1,309 (0.0%)	-	-

Abbreviations: EHR: Electronic health record; PWH: People living with HIV; SOC: Standard-of-care

Supplementary Figure 10: Proportion of participants with blood pressure control NOT on antihypertensives at 6, 12, 18 and 24 months study visits (REACH) (Primary intention-to-treat population)

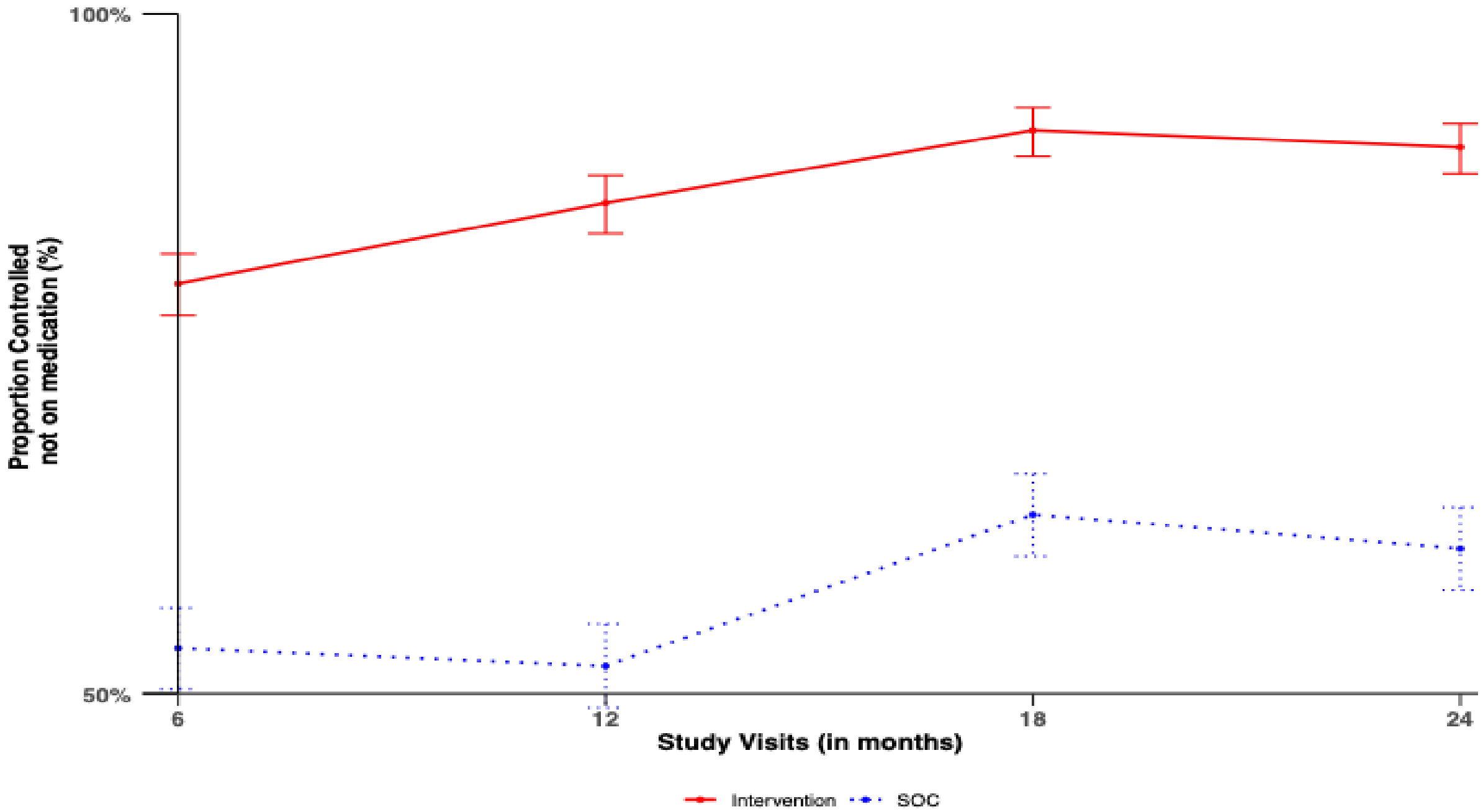

Study Visits	Intervention n/N (%)	Standard-of-care n/N (%)	Unadjusted Risk Ratio (95% CI)	P value
6 months	986/1,230 (80.2%)	586/1,099 (53.3%)	1.52 (1.14 – 2.03)	0.0042
12 months	908/1,055 (86.1%)	539/1,036 (52.0%)	1.64 (1.37 – 1.95)	<0.0001
18 months	907/992 (91.4%)	629/996 (63.2%)	1.45 (1.31 – 1.60)	<0.0001
24 months	946/1,049 (90.2%)	617/1,017 (60.7%)	1.49 (1.34 – 1.65)	<0.0001

Abbreviations: SOC: Standard-of-care

Supplementary Figure 21: Proportion of participants with blood pressure control NOT on anti-hypertensive medications at 6, 12, 18 and 24 months study visit (REACH) (Primary intention-to-treat population)

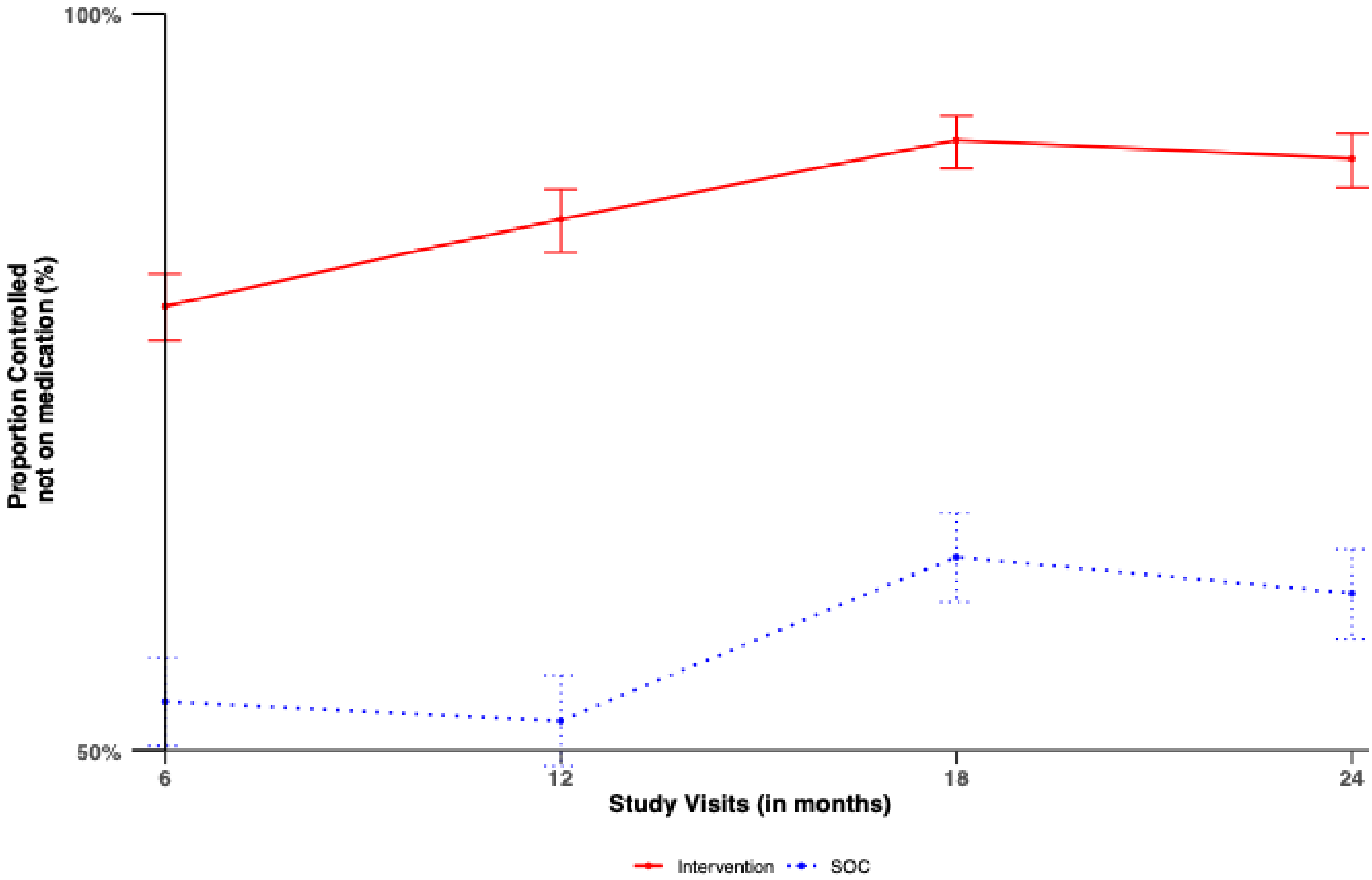

Study Visits	Intervention n/N (%)	Standard-of-care n/N (%)	Unadjusted Risk Ratio (95% CI)	P value
6 months	986/1,230 (80.2%)	586/1,099 (53.3%)	1.52 (1.14 – 2.03)	0.0042
12 months	908/1,055 (86.1%)	539/1,036 (52.0%)	1.64 (1.37 – 1.95)	<0.0001
18 months	907/992 (91.4%)	629/996 (63.2%)	1.45 (1.31 – 1.60)	<0.0001
24 months	946/1,049 (90.2%)	617/1,017 (60.7%)	1.49 (1.34 – 1.65)	<0.0001

Abbreviations: SOC: Standard-of-care

Supplementary Figure 11: Proportion of participants prescribed anti-hypertensive medication at 6, 12, 18 and 24 months study visits (Secondary intention-to-treat population)

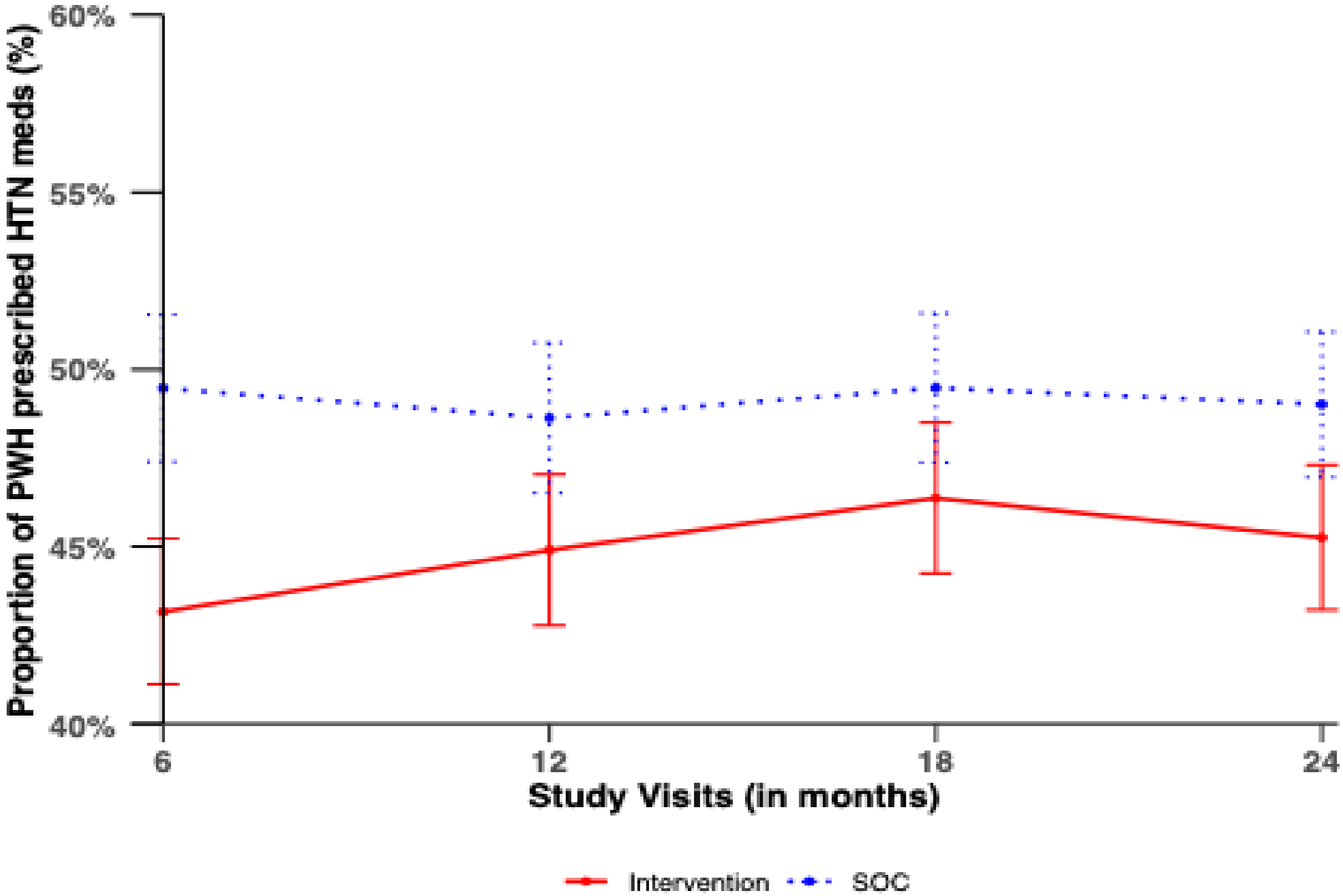

Study Visits	Intervention n/N (%)	Standard-of-care n/N (%)	Unadjusted Risk Ratio (95% CI)	P value
6 months	981/2,273 (43.2%)	1,119/2,262 (49.5%)	0.92 (0.78 – 1.09)	0.35
12 months	955/2,127 (44.9%)	1,064/2,188 (48.6%)	0.97 (0.82 – 1.15)	0.73
18 months	1,002/2,161 (46.4%)	1,089/2,201 (49.5%)	1.00 (0.85 – 1.17)	0.97
24 months	1,053/2,327 (45.3%)	1,140/2,326 (49.0%)	0.98 (0.83 – 1.15)	0.78

Abbreviations: HTN: Hypertension; PWH: People living with HIV

Supplementary Figure 12: Proportion of participants receiving anti-hypertension medication at 6, 12, 18 and 24 months study visits (Secondary intention-to-treat population)

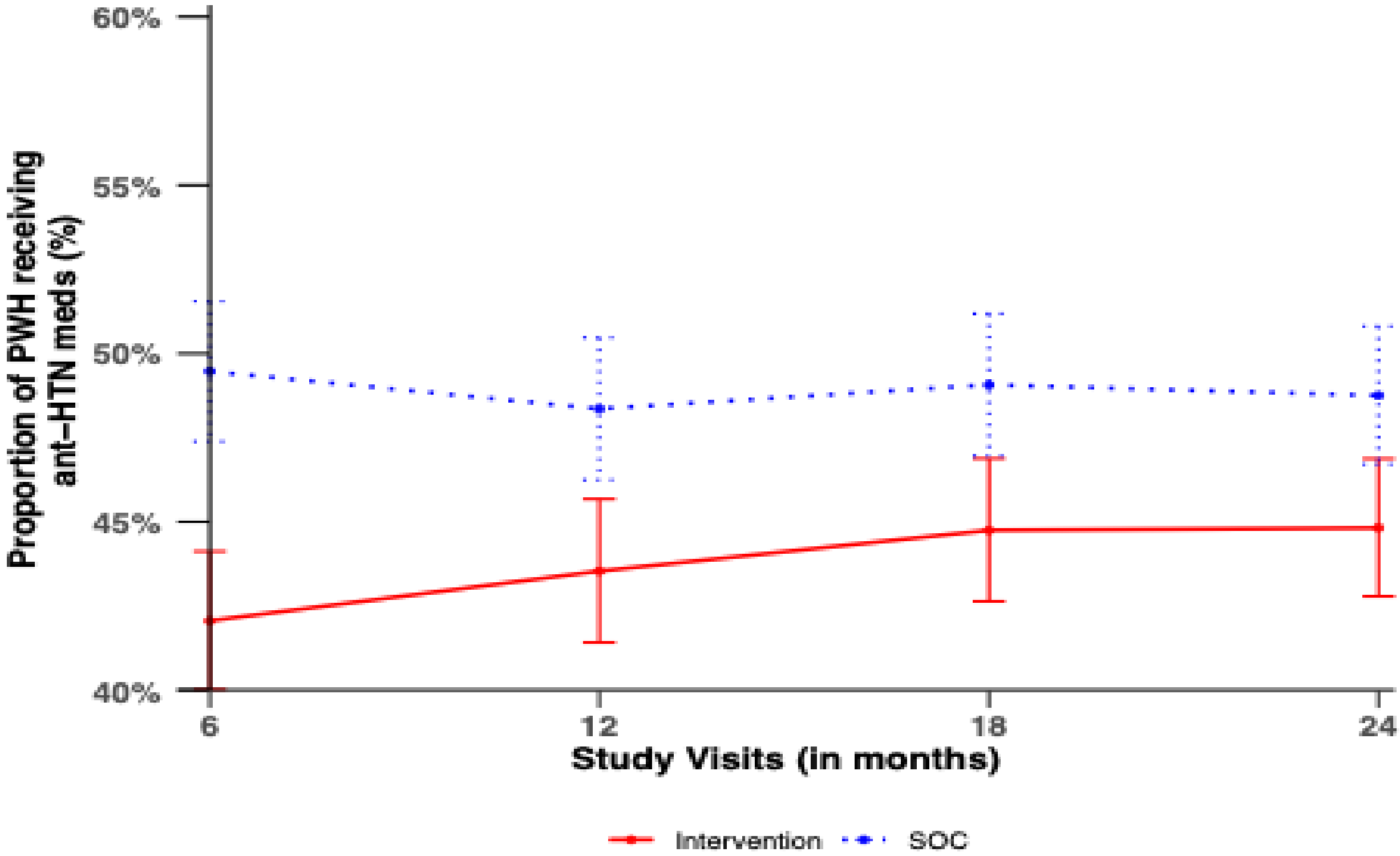

Study Visits	Intervention n/N (%)	Standard-of-care n/N (%)	Unadjusted Risk Ratio (95% CI)	P value
6 months	956/2,273 (42.1%)	1,119/2,262 (49.5%)	0.90 (0.76 – 1.06)	0.22
12 months	926/2,127 (43.5%)	1,058/2,188 (48.4%)	0.95 (0.80 – 1.13)	0.58
18 months	967/2,161 (44.7%)	1,0802,201 (49.1%)	0.98 (0.83 – 1.16)	0.79
24 months	1,043/2,327 (44.8%)	1,134/2,326 (48.8%)	0.97 (0.82 – 1.15)	0.74

Abbreviations: HTN: Hypertension; PWH: People living with HIV

Supplementary Table S3: Unintended effects of InterCARE (Primary intention-to-treat population)

Study visits	Viral load time period	Intervention	Standard-of-care	p-value
Baseline visit				
	Viral suppression within 6 month	1,028/1,042 (98.7%)	1,066/1,083 (98.4%)	0.8001
	Viral suppression within 12 months	1,561/1,580 (98.8%)	1,707/1,726 (98.9%)	0.91
12 months				
	Viral suppression within 6 month	1,222/1,242 (98.4%)	1,174/1,193 (98.4%)	0.46
	Viral suppression within 12 months	1,512/1,536 (98.4%)	1,567/1,585 (98.9%)	0.38
24 months				
	Viral suppression within 6 month	1,143/1,156 (98.9%)	1,272/1,283 (99.1%)	0.64
	Viral suppression within 12 months	475/481 (98.8%)	590/595 (99.2%)	0.55

HIV RNA viral suppression rates among those with a viral load within 6 and 12 months at Baseline, 12 and 24-month visit

Section 3: Secondary outcomes for the Primary Per Protocol population

Supplementary Figure 13: Proportion of participants aware of their hypertension status at 6, 12, 18 and 24 months study visits (REACH) (Primary per-protocol population)

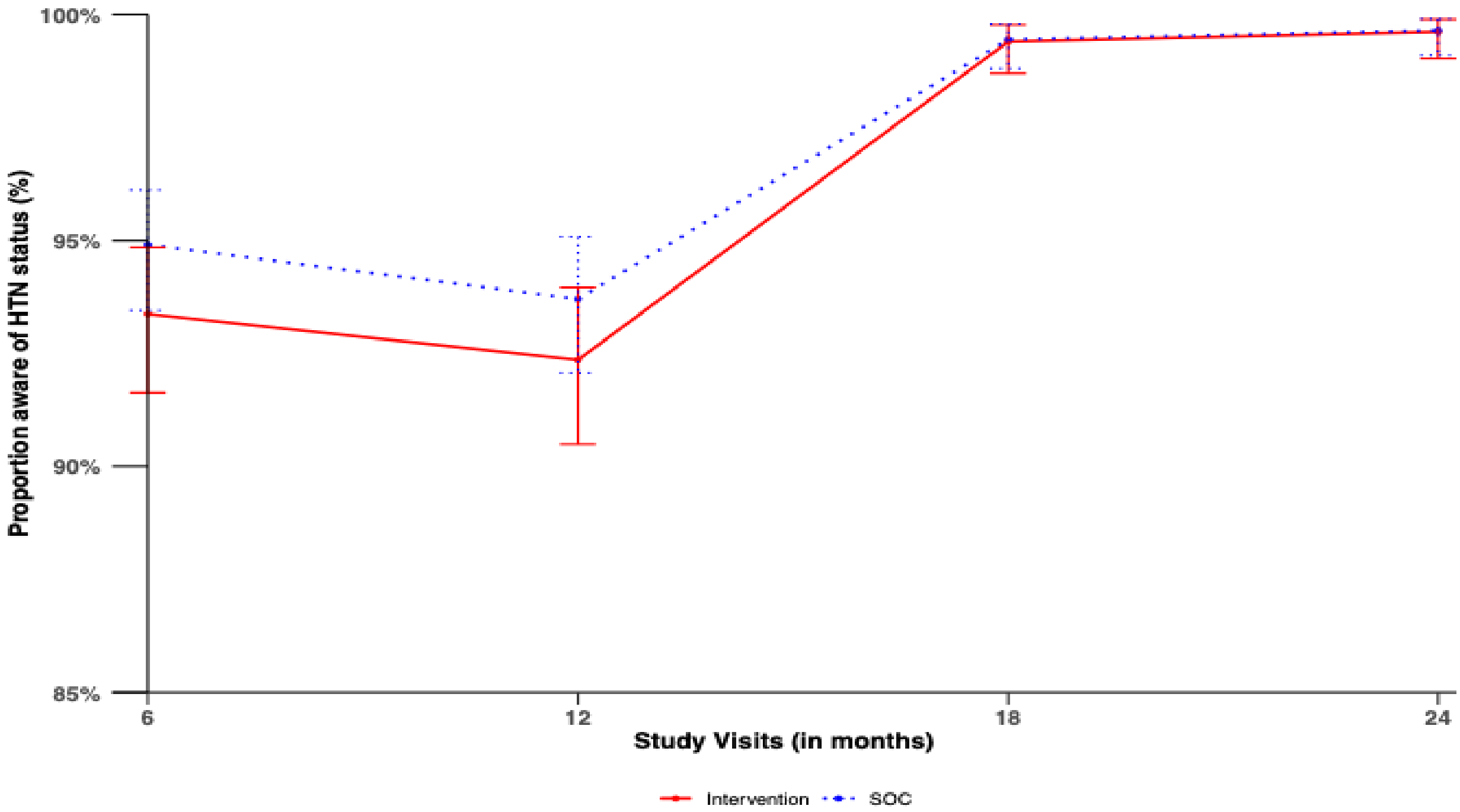

Study Visits	Intervention n/N (%)	Standard-of-care n/N (%)	Unadjusted Risk Ratio (95% CI)	P value
6 months	916/981 (93.4%)	1,062/1,119 (94.9%)	1.00 (0.92 – 1.10)	0.94
12 months	882/955 (92.4%)	997/1,064 (93.7%)	0.99 (0.91 – 1.09)	0.91
18 months	996/1,002 (99.4%)	1,083/1,089 (99.4%)	1.00 (0.92 – 1.09)	0.92
24 months	1,049/1,053 (99.6%)	1,136/1,140 (99.6%)	1.00 (0.92 – 1.09)	0.99

Abbreviations: HTN: Hypertension; SOC: Standard-of-care

Supplementary Figure 14: Mean decrease in systolic and diastolic blood pressure at 6, 12, 18 and 24 months study visits (EFFECTIVENESS) (Primary per-protocol population)

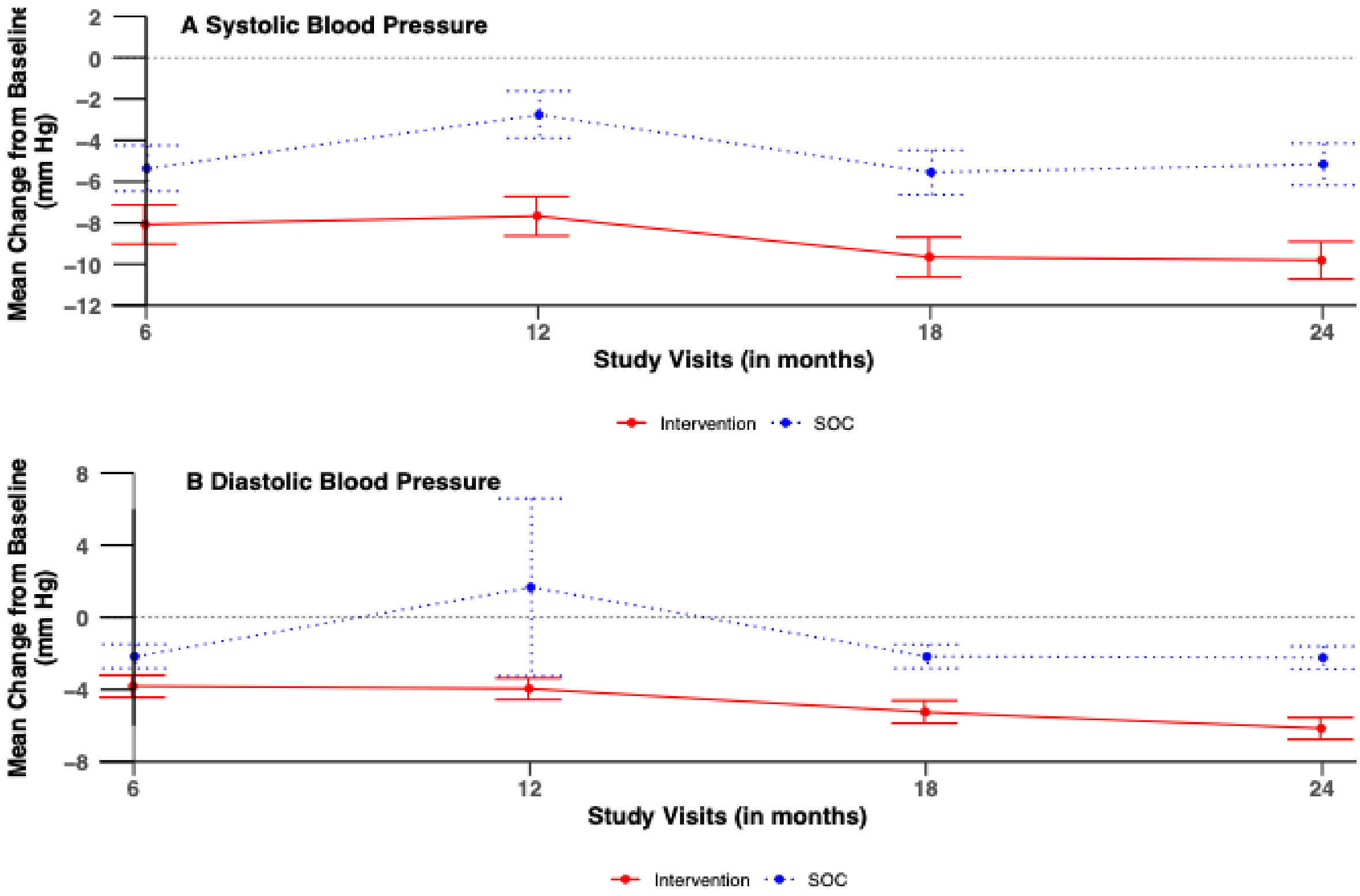

Study Visit	6 months		12months		18months		24 months	
	Intervention (N= 981)	SOC (N= 1,119)	Intervention (N= 955)	SOC (N=1,064)	Intervention (N= 1,002)	SOC (N= 1,089)	Intervention (N= 1,053)	SOC (N= 1,140)
Systolic Mean change (SD)	-8.1(14.9)	-5.4(18.7)	-7.7(14.5)	-2.8(18.5)	-9.7(15.6)	-5.6(18.1)	-9.8(15.0)	-5.2(17.4)
Systolic Mean (SD)	130 (14.9)	133 (18.9)	130 (14.5)	135 (18.5)	128 (15.6)	132 (18.1)	133 (17.4)	128 (15.0)
Mean diff (95%CI)	-1.93 (-6.3, 1.44)		-3.79 (-6.54, -1.04)		-3.42 (-7.22, 0.38)		-4.92 (-7.86, -1.97)	
P value	0.32		0.011		0.074		0.0072	
Diastolic mean change (SD)	-3.8(9.7)	-2.2(11.2)	-3.9(9.2)	1.7(80.0)	-5.2(10.2)	-2.2(11.0)	-6.1(10.0)	-2.2(10.8)
Diastolic mean (SD)	80.2(9.70)	81.8(11.2)	80.1(9.19)	85.7(80.0)	78.8(10.2)	81.8(11.0)	77.9(10.0)	81.8(10.8)
Mean diff (95%CI)	-1.29 (-4.5, 1.91		-5.33 (-12.6, 1.93)		-2.43(-5.42, 0.57)		-3.9 (-7.33, 0.46)	
P value	0.39		0.12		0.103		0.032	

Abbreviations: Hg: mercury; MM: millimetres; SOC: standard-of-care

Supplementary Figure 15: Proportion of participants receiving HIV and hypertension care at same clinic visit at 6, 12, 18 and 24 months study visit (Adoption) (Primary per-protocol population):

Integrated visit defined as a visit where HIV care visit coincides with HTN medications prescription on EHR or CVD risk (exercise, diet, salt intake) counselling.

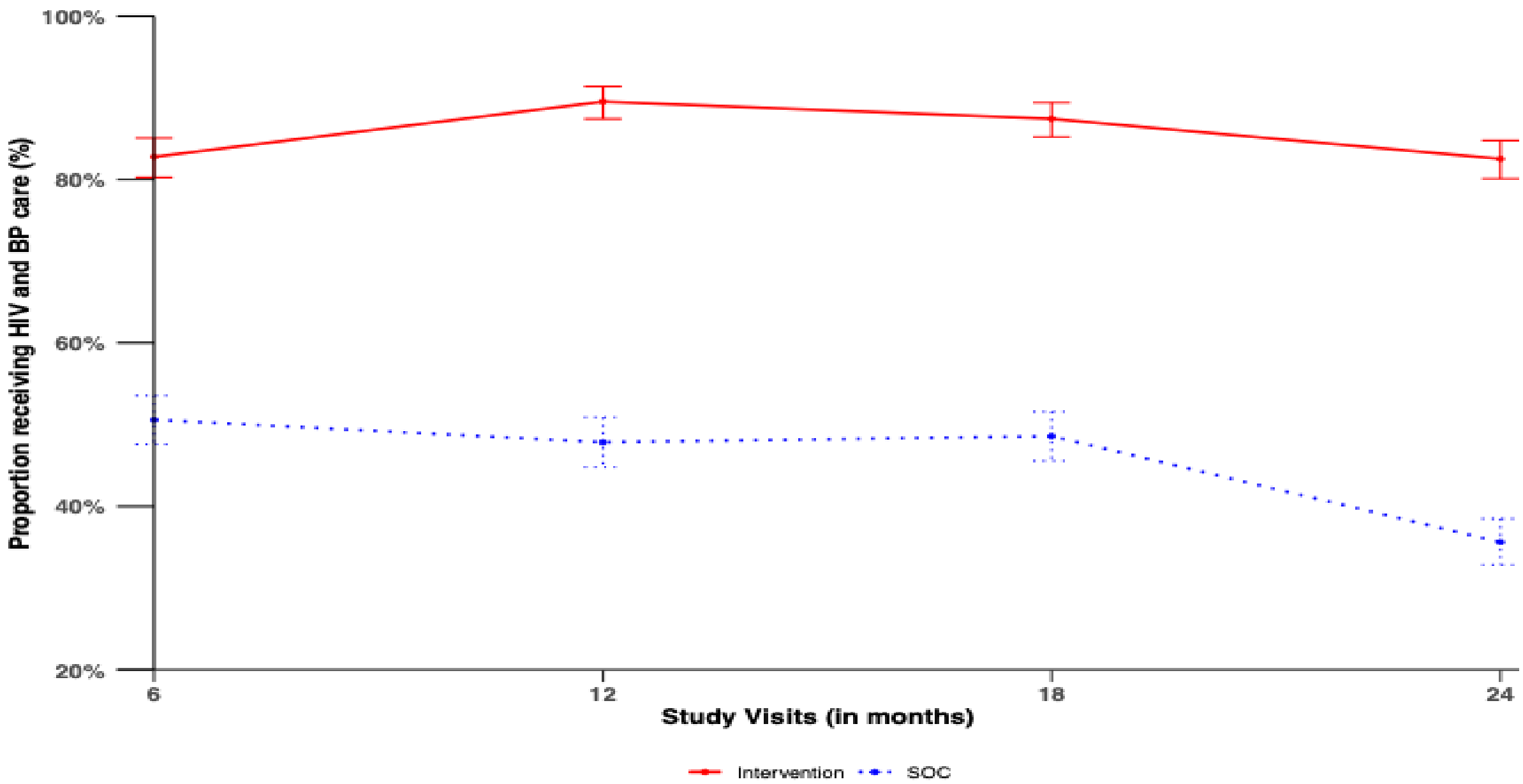

Study Visits	Intervention n/N (%)	Standard-of-care n/N (%)	Unadjusted Risk Ratio (95% CI)	P value
6 months	812/981 (82.8%)	566/1,119 (50.6%)	1.78 (1.05 – 3.01)	0.031
12 months	855/955 (89.5%)	509/1,064 (47.8%)	2.06 (1.39 – 3.03)	0.0003
18 months	876/1,002 (87.4%)	529/1,089 (48.6%)	2.99 (1.21 – 7.35)	0.017
24 months	869/1,053 (82.5%)	406/1,140 (35.6%)	3.92 (1.51 – 10.16)	0.0049

Abbreviations: BP: Blood pressure; HIV: Human immunodeficiency virus; SOC: Standard-of-care

Supplementary Figure 16: Proportion of participants with >70% pass on Heart Disease Fact Questionnaire (HDFQ) (EFFECTIVENESS) (Primary per-protocol population)

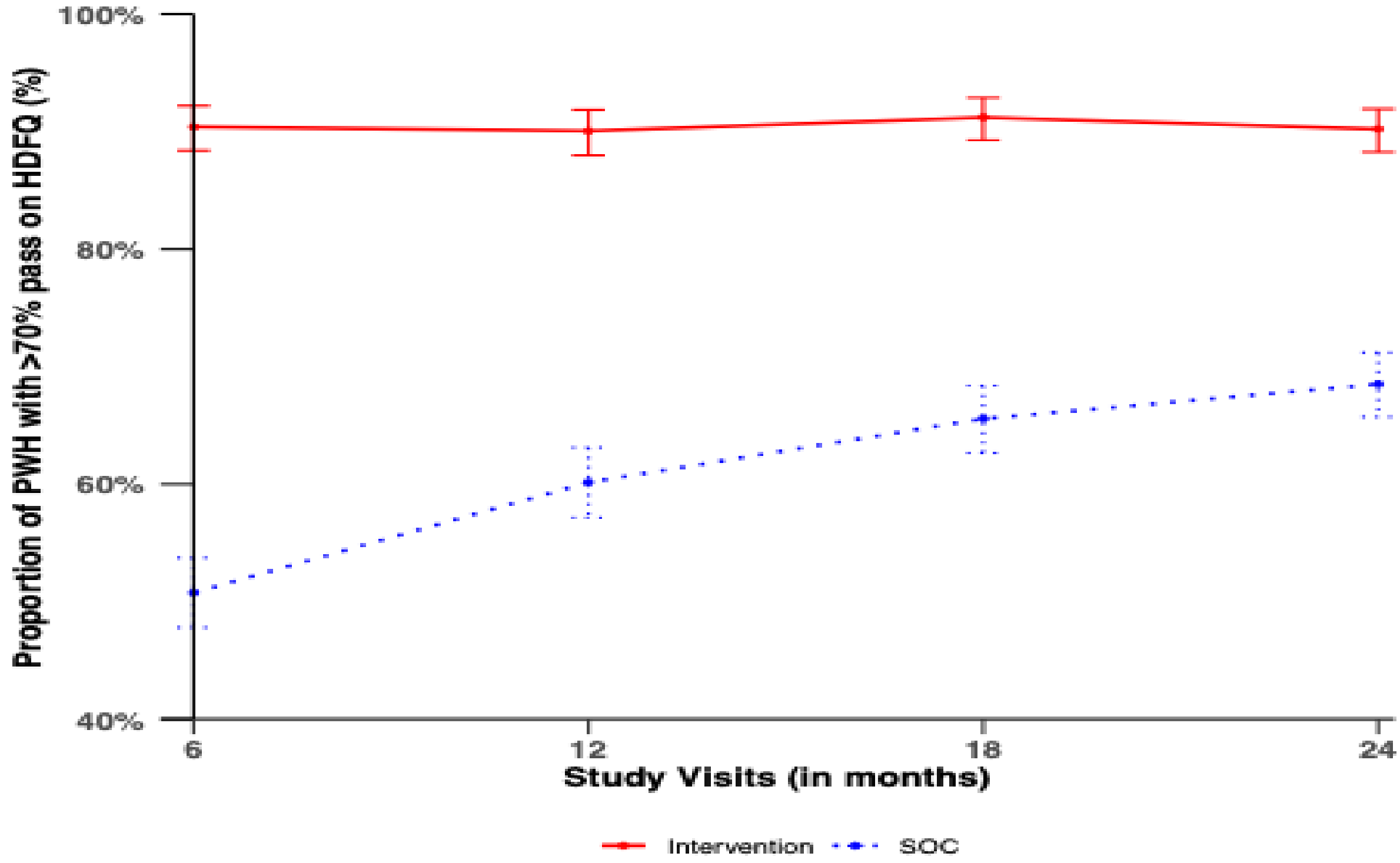

Study Visits	Intervention n/N (%)	Standard-of-care n/N (%)	Unadjusted Risk Ratio (95% CI)	P value
6 months	887/981 (90.4%)	568/1,119 (50.8%)	2.41 (1.12 – 5.20)	0.025
12 months	860/955 (90.1%)	640/1,064 (60.2%)	1.46 (0.94 – 2.25)	0.0902
18 months	914/1,002 (91.2%)	714/1,089 (65.6%)	1.26 (0.92 – 1.71)	0.15
24 months	950/1,053 (90.2%)	781/1,140 (68.5%)	1.21 (1.07 – 1.37)	0.0020

Abbreviations: HDFQ: Heart disease fact questionnaire; HIV: Human immunodeficiency virus; SOC: Standard-of-care

Supplementary Figure 17: Proportion of participants with blood pressure control at baseline who maintain blood pressure control in next study visit: (MAINTENANCE) (Primary per-protocol population)

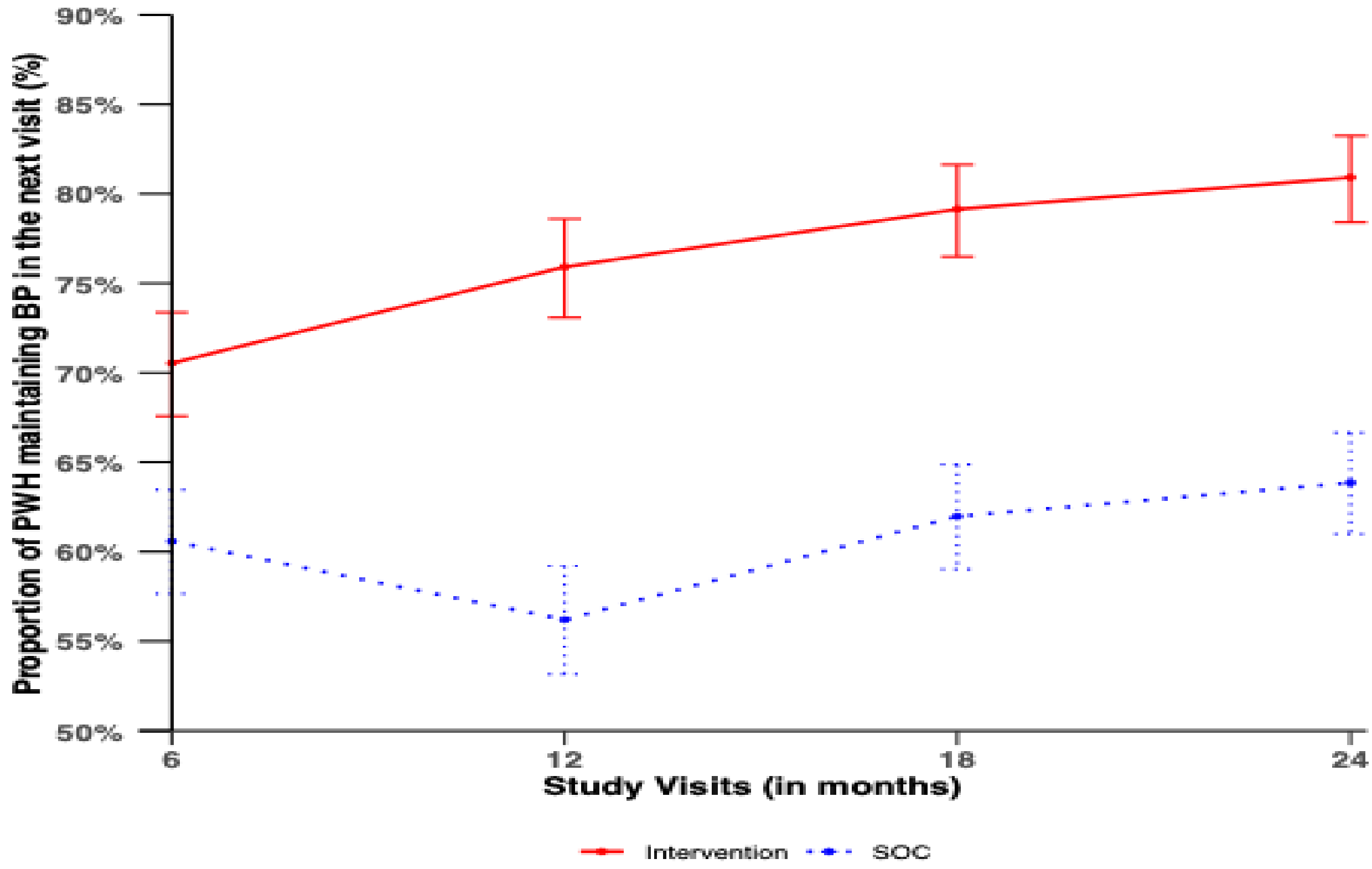

Study Visits	Intervention n/N (%)	Standard-of-care n/N (%)	Unadjusted Risk Ratio (95% CI)	P value
6 months	692/981 (70.5%)	678/1,119 (60.6%)	1.20 (1.07 – 1.34)	0.0021
12 months	725/955 (75.9%)	598/1,064 (56.2%)	1.33 (1.13 – 1.56)	0.0006
18 months	793/1,002 (79.1%)	675/1,089 (62.0%)	1.28 (1.12 – 1.46)	0.0002
24 months	852/1,053 (80.9%)	728/1,140 (63.9%)	1.28 (1.16 – 1.42)	<0.0001

Abbreviations: BP: Blood pressure; PWH: People living with HIV; SOC: Standard-of-care

Supplementary Figure 18: Proportion of participants with 10-year cardiovascular risk calculated in the electronic health records (Primary per-protocol population).

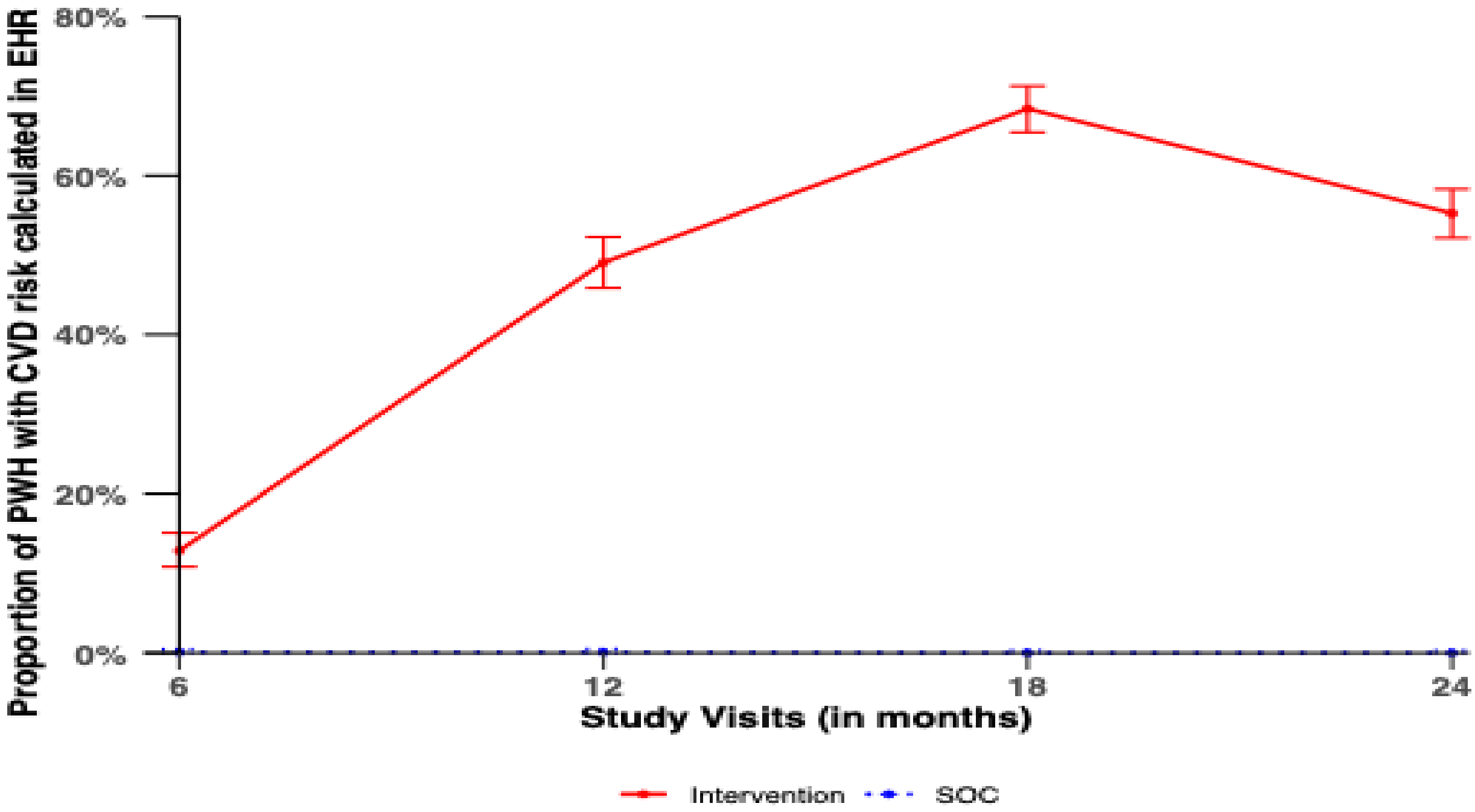

Study Visits	Intervention n/N (%)	Standard-of-care n/N (%)	Unadjusted Risk Ratio (95% CI)	P value
6 months	126/981 (12.8%)	1/1,119 (0.1%)	159.07 (22.19 – 1140.24)	<0.0001
12 months	469/955 (49.1%)	1/1,064 (0.1%)	461.09 (64.73 – 3284.33)	<0.0001
18 months	686/1,002 (68.5%)	0/1,089 (0.0%)	-	-
24 months	582/1,053 (55.3%)	0/1,140 (0.0%)	-	-

Abbreviations: CVD: Cardiovascular diseases; EHR: Electronic health records; PWH: People living with HIV; SOC: Standard-of-care

Supplementary Figure 19: Proportion of participants on medications with correct regimen as per the national guidelines at 6, 12, 18 and 24 months study visits (Primary per-protocol population)

Guideline concordance defined as CCB or HCTZ for monotherapy, CCB/HCTZ combo or with ACEI/ARB for dual therapy, and then CCB/HCTZ/ACEI/ARB for triple therapy, and finally, CCB/HCTZ/ACEI/ARB plus alpha/Beta-blocker

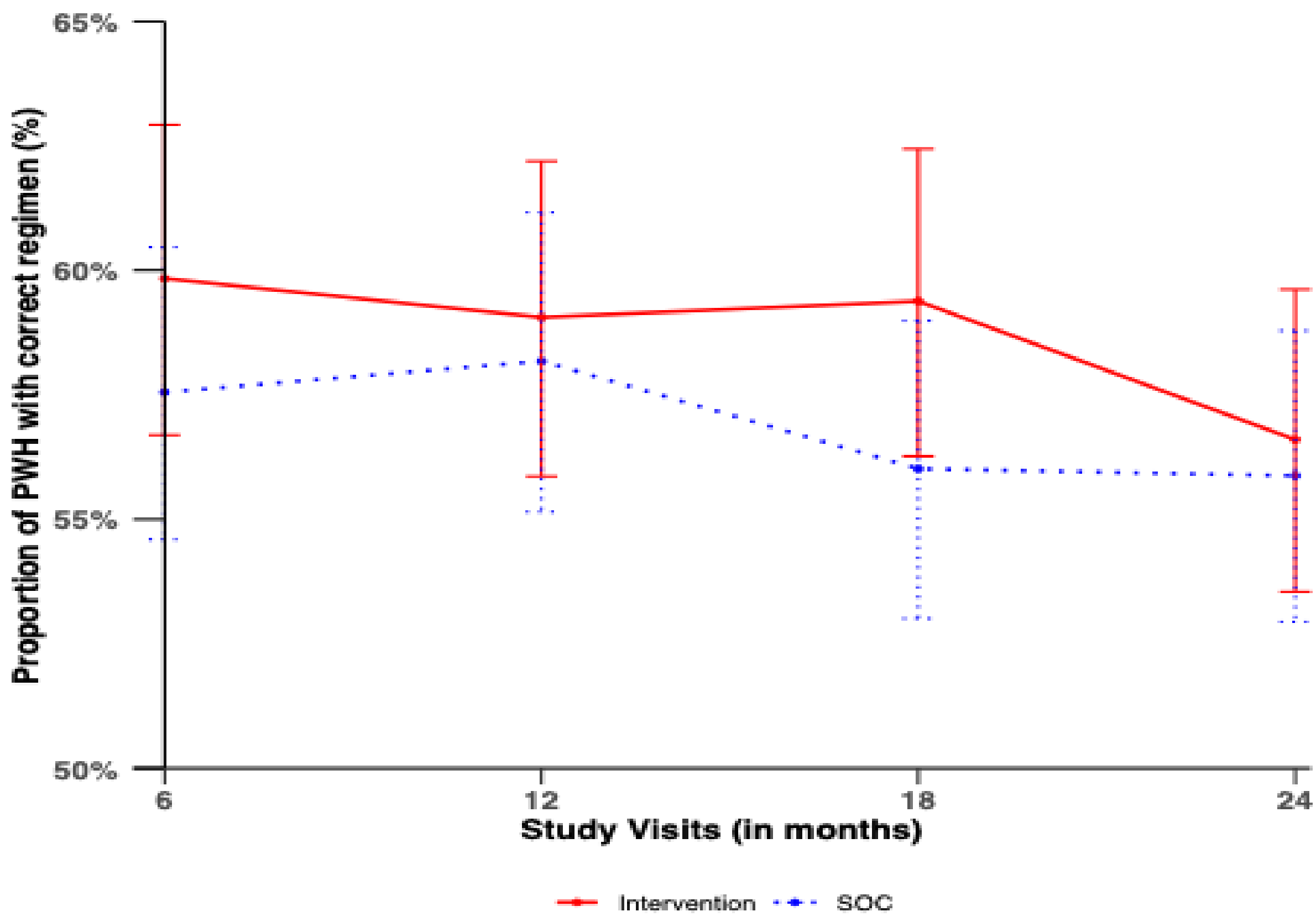

Study Visits	Intervention n/N (%)	Standard-of-care n/N (%)	Unadjusted Risk Ratio (95% CI)	P value
6 months	587/981 (59.8%)	644/1,119 (57.6%)	1.06 (0.94 – 1.19)	0.33
12 months	564/955 (59.1%)	619/1,064 (58.2%)	1.02 (0.91 – 1.15)	0.75
18 months	595/1,002 (59.4%)	610/1,089 (56.0%)	1.06 (0.94 – 1.19)	0.33
24 months	596/1,053 (56.6%)	637/1,140 (55.9%)	1.00 (0.89 – 1.13)	0.96

Abbreviations: ACEI: Angiotensin converting enzyme inhibitor; ARB: Angiotensin receptor blocker; CCB: Calcium channel blocker; HCTZ: Hydrochlorothiazide; SOC: Standard-of-care, PWH: People living with HIV

Supplementary Figure 20: Proportion of participants with waist and hip circumference recorded in Electronic Health Records at 6, 12, 18 and 24 months study visits (Primary per-protocol population)

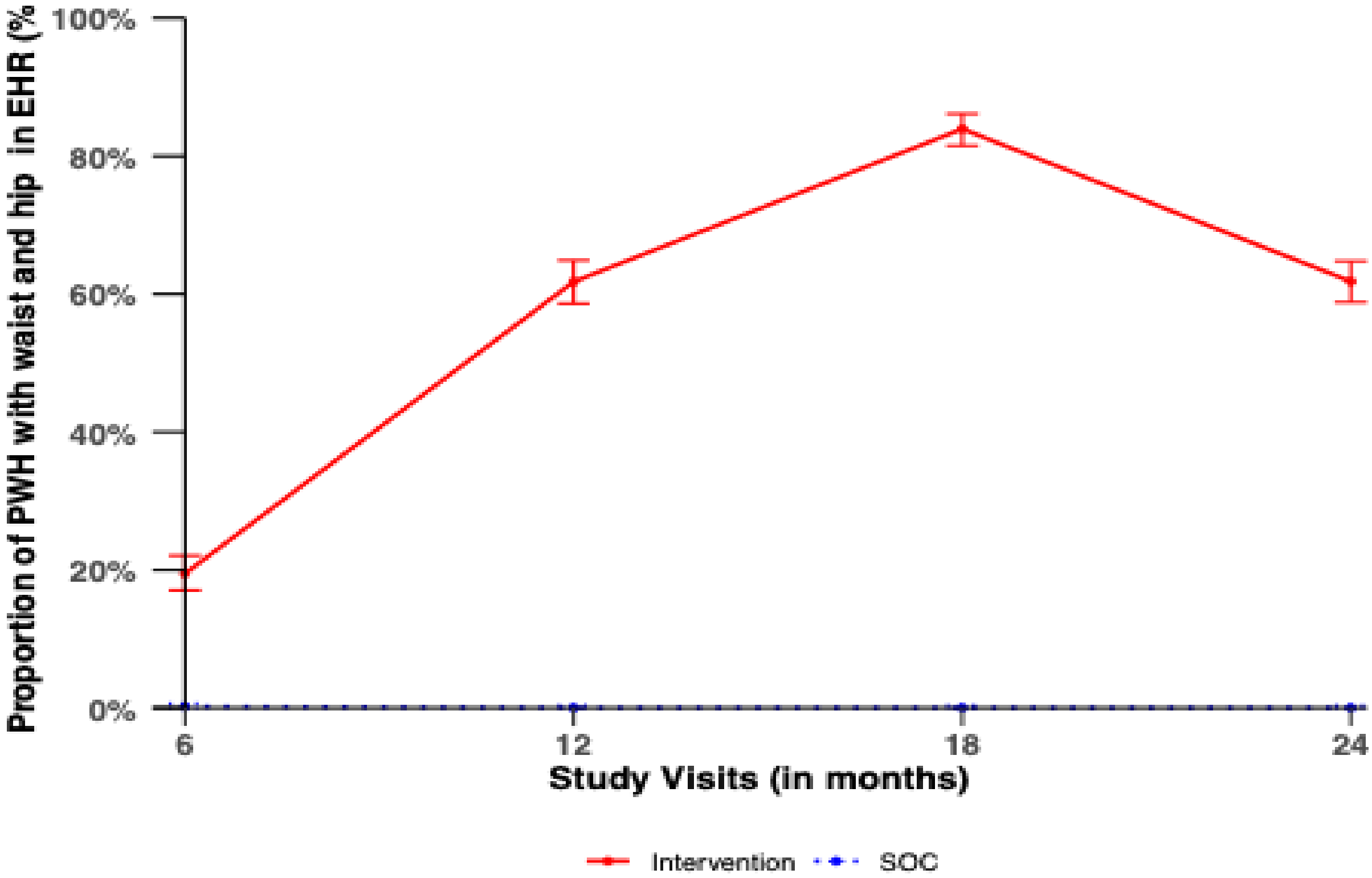

Study Visits	Intervention n/N (%)	Standard-of-care n/N (%)	Unadjusted Risk Ratio (95% CI)	P value
6 months	191/981 (19.5%)	2/1,119 (0.2%)	120.82 (29.94 – 487.57)	<0.0001
12 months	590/955 (61.8%)	0/1,064 (0.0%)	-	-
18 months	841/1,002 (83.9%)	0/1,089 (0.0%)	-	-
24 months	651/1,053 (61.8%)	0/1,140 (0.0%)	-	-

Abbreviations: EHR: Electronic health records; PWH: People living with HIV; SOC: Standard-of-care

Supplementary Figure 22: Proportion of participants prescribed anti-hypertensive medication at 6, 12, 18 and 24 months study visits (Secondary per-protocol population)

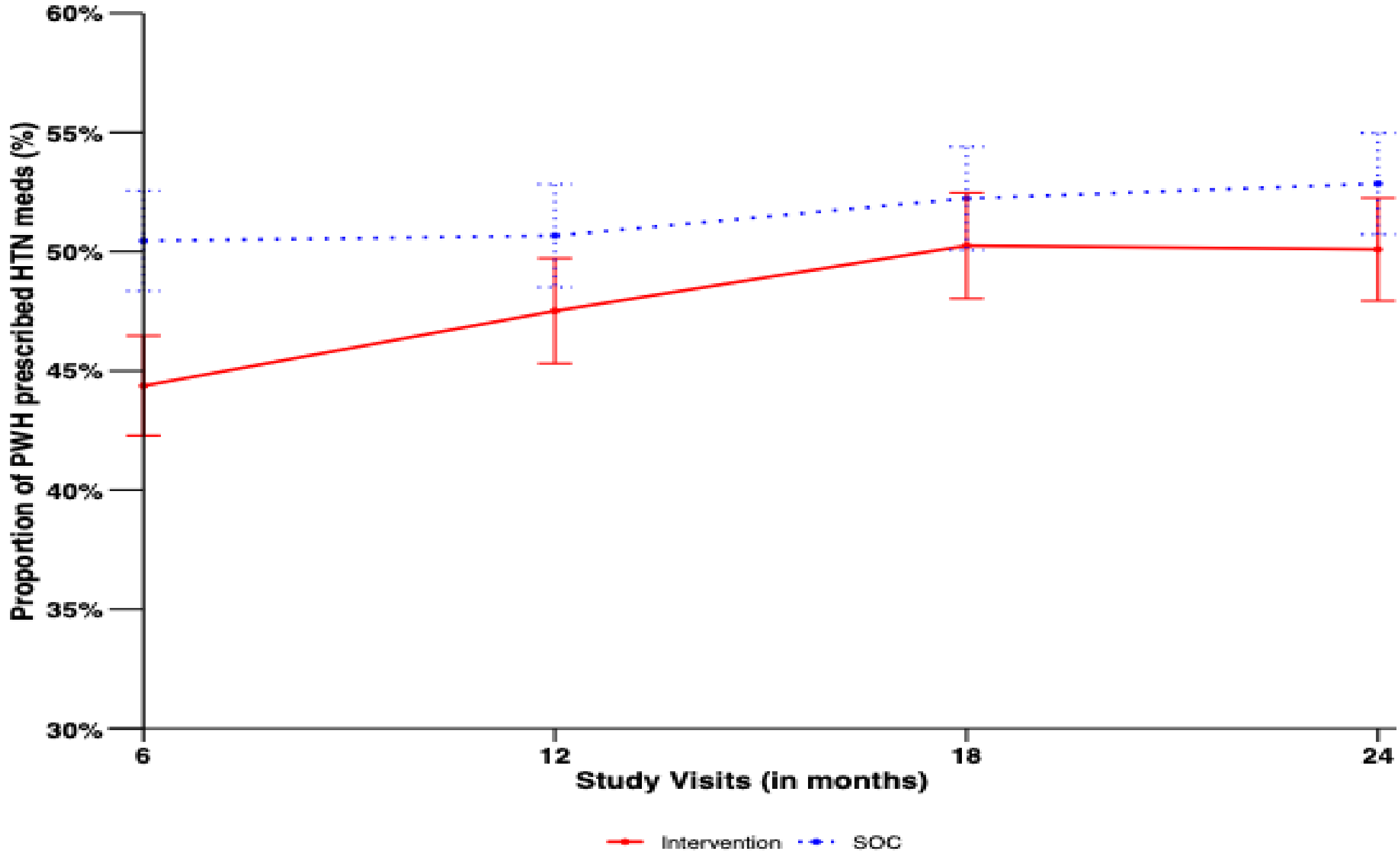

Study Visits	Intervention n/N (%)	Standard-of-care n/N (%)	Unadjusted Risk Ratio (95% CI)	P value
6 months	981/2,211 (44,4%)	1,119/2,218 (50.5%)	0.93 (0.79 – 1.09)	0.36
12 months	955/2,010 (47.5%)	1,064/2,100 (50.7%)	0.99 (0.85 – 1.15)	0.87
18 months	1,002/1,994 (50.3%)	1,089/2,085 (52.2%)	1.02 (0.89 – 1.17)	0.75
24 months	1,053/2,102 (50.1%)	1,140/2,157 (52.9%)	1.00 (0.86 – 1.16)	0.99

Abbreviations: HTN: Hypertension; PWH: People living with HIV; SOC: Standard-of-care

Supplementary Figure 23: Proportion of participants with receiving anti-hypertension medication at 6, 12, 18 and 24 months study visit (Secondary per-protocol population).

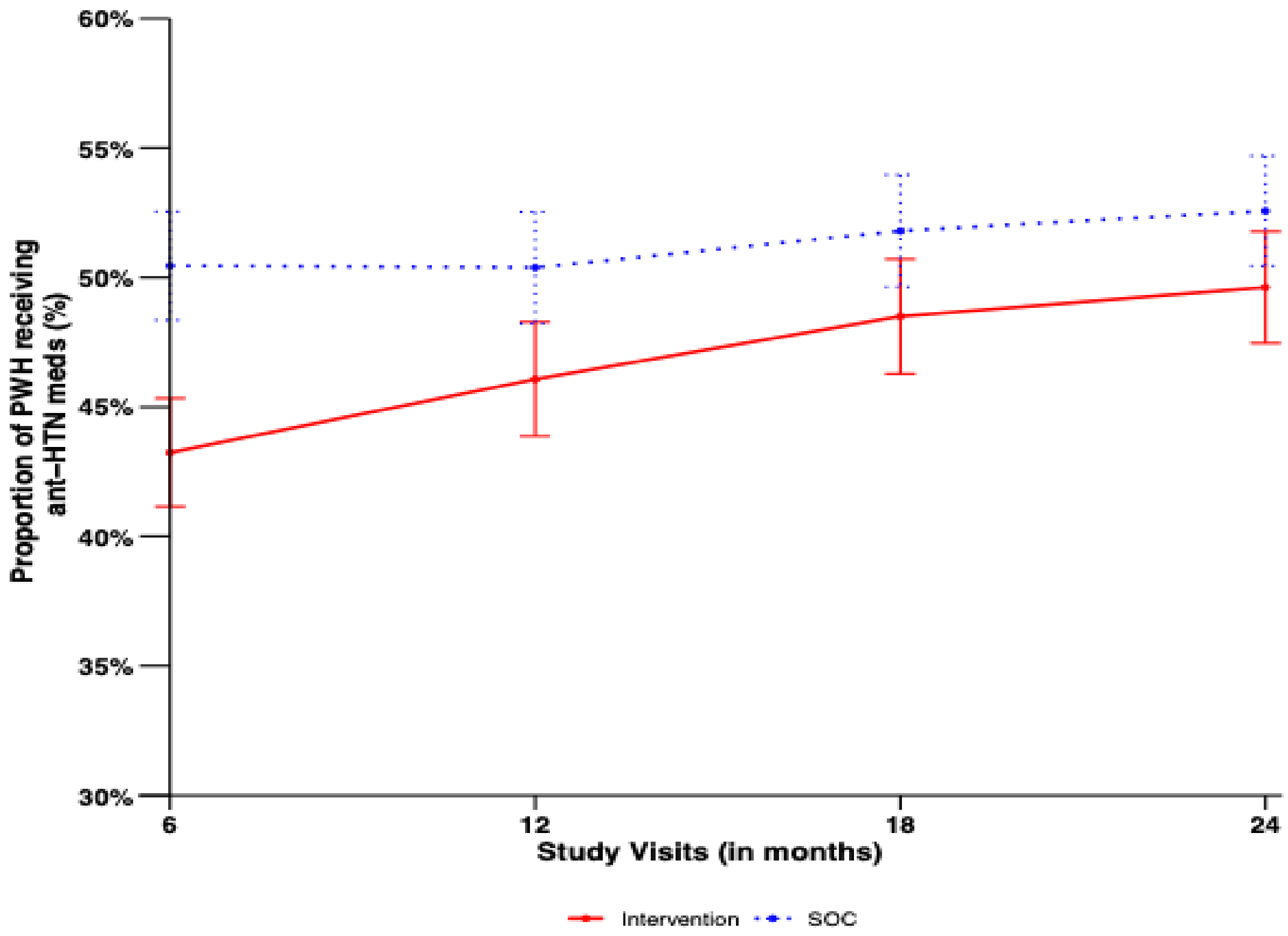

Study Visit	Intervention n/N (%)	Standard-of-care n/N (%)	Unadjusted Risk Ratio (95% CI)	P value
6 months	956/2,211 (43.2%)	1,119/2,218 (50.5%)	0.91 (0.77 – 1.06)	0.22
12 months	926/2,010 (46.1%)	1,058/2,100 (50.4%)	0.97 (0.83 – 1.13)	0.70
18 months	967/1,994 (48.5%)	1,068/2,085 (51.8%)	1.00 (0.87 – 1.16)	0.97
24 months	1,043/2,102 (49.6%)	1,117/2,157 (52.6%)	0.99 (0.85 – 1.16)	0.95

Abbreviations: Anti-HTN: Antihypertensive Meds: Medication; PWH: People living with HIV; SOC: Standard-of-care

Supplementary Table S4: Proportion of intervention clinics that implemented all three InterCARE strategies at 24 months study visit (Primary intention-to-treat population)

Site name	EHR	Mopati	HCW training
Sefophe	100%	325/326 (99.7%)	11/11 (100%)
Lentweletau	100%	327/374 (87.4%)	12/22 (54.5%)
Mmathethe	100%	284/338 (84.0%)	16/19 (84.2%)
Oodi	100%	295/347 (85.0%)	19/22 (86.4%)
Molapowabojang	100%	304/308 (98.7%)	21/21 (100%)
Tati-siding	100%	460/470 (97.9%)	12/30 (40.0%)
Nkange	100%	145/164 (88.4%)	27/27 (100%)

Abbreviations: EHR: Electronic health record; HCW: Healthcare worker

Supplementary Table S5: Unintended effects of InterCARE at 12 and 24 months study visits (Secondary intention-to-treat population)

Reported by treatment arms (including grade as per DAIDS)

12 months follow up

	Intervention (N = 2,127)	Standard-of-Care (N = 2,188)	P-value
Grade I	0 (0.0%)	0 (0.0%)	-
Grade II	0 (0.0%)	1 (0.05%)	>0.99
Grade III	3 (0.14%)	3 (0.14%)	>0.99
Grade IV	0 (0.0%)	4 (0.17%)	0.13
Grade V	22 (1.0%)	20 (0.91%)	0.69
Viral Suppression	1,759 (98.6%)	1,821 (99.0%)	0.38

24 months follow up

	Intervention N = 2,327	Standard-of-care N = 2,326	p-value
Grade I	0 (0.0%)	0 (0.0%)	-
Grade II	1 (0.04%)	1 (0.04%)	>0.99
Grade III	9 (0.39%)	5 (0.21%)	0.42
Grade IV	0 (0.0%)	4 (0.17%)	0.062
Grade V	39 (1.68%)	30 (1.29%)	0.28
Viral Suppression	1,453 (98.9%)	1,626 (99.2%)	0.503

Abbreviations: DAIDS = Department of Acquired Immunodeficiency Syndrome

Section 4: Sensitivity Analysis of the primary outcomes

Supplementary Table S6: Sensitivity analysis of the primary outcomes adjusting for cluster level characteristics using the primary intention-to-treat population at 12 months study visit

12 months	**Intervention n/N (%)**	**Standard-of-care n/N (%)**	**Unadjusted Risk # Ratio (95% CI)**	**P value**	**Adjusted Risk * Ratio (95% CI)**	**P value**
Blood pressure control	719/1072 (67.1%)	593/1152 (51.5%)	1.29 (1.10 – 1.51)	0.0013	1.23 (1.04 – 1.45)	0.018
Antihypertensive medicine electronic prescribing	428/1072 (39.9%)	124/1152 (10.8%)	5.95 (2.18 – 16.2)	0.0005	6.65 (2.23 – 19.78)	0.0007

Risk ratios with 95% CIs were calculated from generalised linear mixed models (GLMMs) with a Poisson distribution and log link function and with the treatment (treatment vs. control) as a fixed effect and clusters and pairs as random effects
* The above GLMMs were further adjusted for age, sex, level of income, waist–hip ratio, physical activity (estimated number of minutes walked per week), and alcohol and tobacco consumption with clusters and pairs of clinics as fixed effects.

Supplementary Table S7: Adjusted generalised linear mixed model for primary effectiveness outcome with clinic pairs as fixed effects

Variable	Adjusted Risk ratio	95% CI	p-value
Intervention	1.27	1.11 – 1.44	0.0003
Clinic Pairs			
Pair 1 (Ref)	1.00	–	–
Pair 2	1.36	1.09 – 1.71	0.0070
Pair 3	1.29	1.05 – 1.59	0.014
Pair 4	1.25	1.01 – 1.54	0.039
Pair 5	1.13	0.93 – 1.38	0.22
Pair 6	1.46	1.19 – 1.79	0.0004
Pair 7	1.20	0.98 – 1.48	0.076
Sex			
Male (ref)	1.00		
Female	1.26	1.10 – 1.44	0.0008
Age (in years)			
≤40 years (ref)	1.00		
>40 years	1.30	1.06 – 1.59	0.011
Income			
<P 5,000.00 (ref)	1.00		
≥P 5,000.00	1.05	0.84 – 1.31	0.67
Exercise			
None (ref)	1.00		
Some exercise	1.04	0.92 – 1.19	0.52
Waist hip ratio			
Normal (ref)	1.00		
Not normal	0.99	0.87 – 1.11	0.83
Alcohol			
No (ref)	1.00		
Yes	0.95	0.80 – 1.14	0.59
Tobacco			
No (ref)	1.00		
Yes	1.03	0.84 – 1.26	0.78

Supplementary Figure 23: Post-Hoc Pairwise Comparisons of Clinic Pairs for Effectiveness Outcome

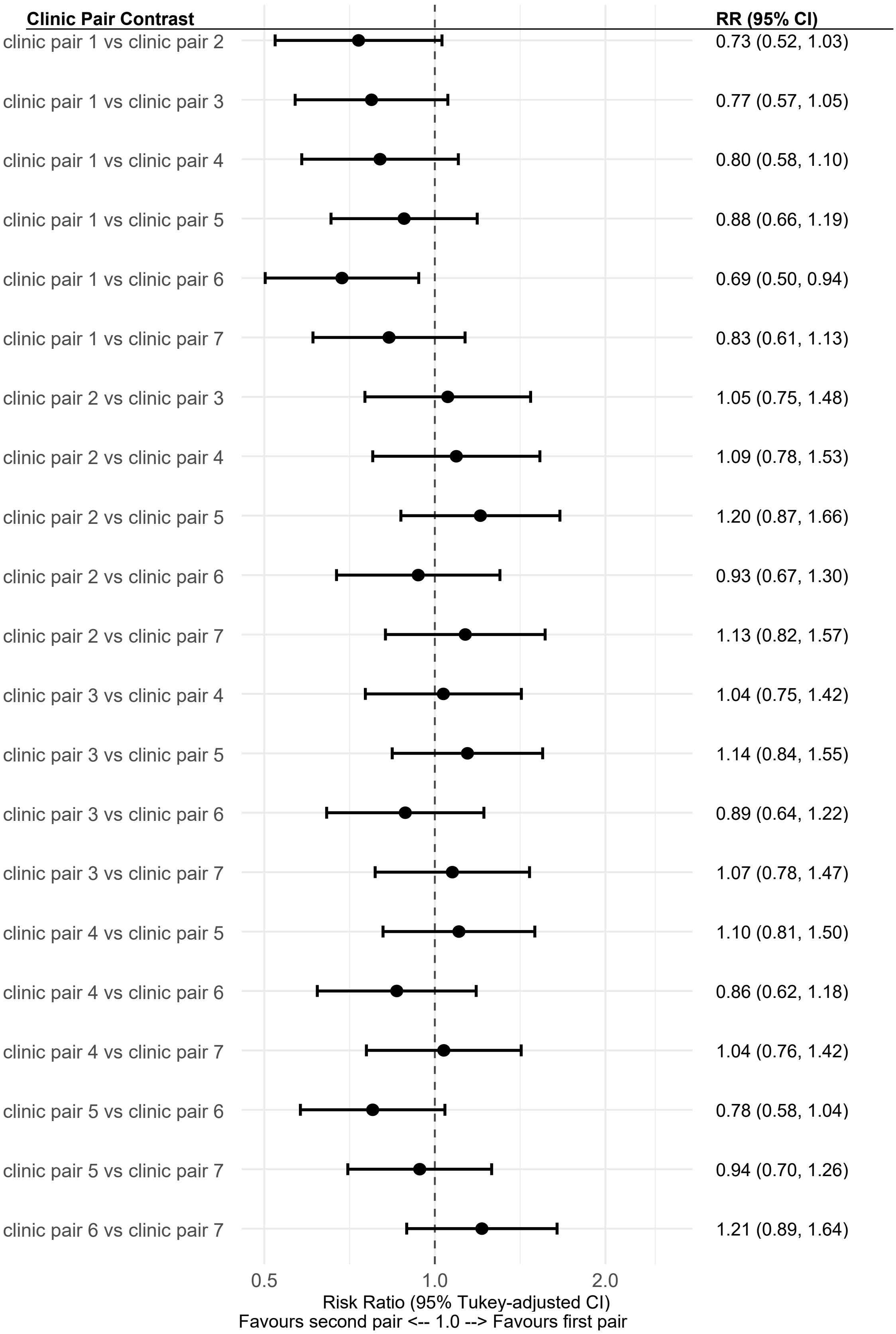

Supplementary Table S8: Adjusted generalised linear mixed model for primary implementation outcome with clinic pairs as fixed effects

Variable	Adjusted Risk ratio	95% CI	P-value
Intervention	5.55	2.57 – 12.0	<0.0001
Clinic Pairs			
Pair 1 (Ref)	1.00	–	–
Pair 2	0.20	0.05 – 0.86	0.031
Pair 3	0.21	0.05 – 0.81	0.024
Pair 4	0.19	0.05 – 0.73	0.016
Pair 5	0.55	0.15 – 1.96	0.35
Pair 6	0.35	0.09 – 1.37	0.13
Pair 7	0.52	0.14 – 1.86	0.32
Sex			
Male (ref)	1.00	–	–
Female	0.97	0.80 – 1.17	0.71
Age (in years)			
≤40 years (ref)	1.00	–	–
>40 years	1.20	0.89 – 1.62	0.24
Income			
<P 5,000.00 (ref)	1.00	–	–
≥P 5,000.00	1.07	0.76 – 1.50	0.72
Exercise			
None (ref)	1.00	–	–
Some exercise	1.06	0.87 – 1.29	0.56
Waist hip ratio			
Normal (ref)	1.00	–	–
Not normal	0.91	0.75 – 1.11	0.37
Alcohol			
No (ref)	1.00	–	–
Yes	1.04	0.80 – 1.35	0.77
Tobacco			
No (ref)	1.00	–	–
Yes	0.97	0.71 – 1.33	0.86

Supplementary Figure 24: Post-Hoc Pairwise Comparisons of Clinic Pairs for Implementation Outcome

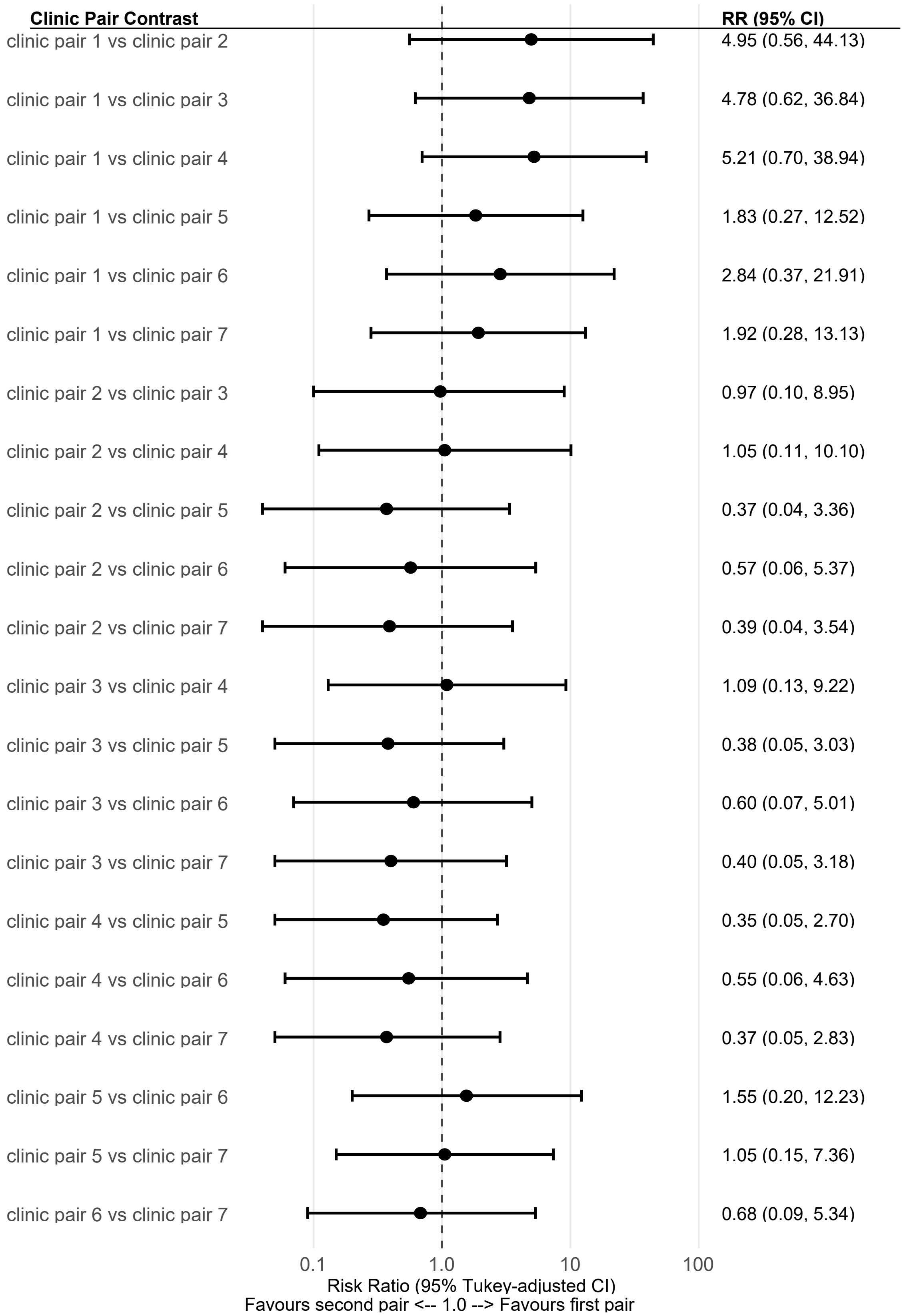

Supplement: Supplementary file 2 [file NIHMS2215925-supplement-2.pdf]
