# Supplementary material for: Integrating hypertension care into the existing HIV services package in Botswana (InterCARE): a pair-matched, cluster-randomised, type 2 hybrid effectiveness–implementation trial
Source: Lancet Glob Health. Author manuscript; Available in PMC 2026 Oct 23. (PMC13691192; doi:10.1016/j.langlo.2026.104041)
Supplement: Supplementary file 3 [file NIHMS2215925-supplement-3.pdf]

THE LANCET
Global Health

Supplementary appendix 3

This appendix formed part of the original submission and has been peer reviewed.
We post it as supplied by the authors.

Supplement to: Mosepele M, Gaolathe T, Kebotsamang K, et al. Integrating hypertension care into the existing HIV services package in Botswana (InterCARE): a pair-matched, cluster-randomised, type 2 hybrid effectiveness–implementation trial. *Lancet Glob Health* 2026. https://doi.org/10.1016/j.langlo.2026.104041

Equitable Partnership Declaration

If any questions do not apply to your study, please indicate "N/A" for "not applicable.
For more information on how to complete this form see the Information for Authors document.

Researcher considerations

1. Please detail the involvement that researchers who are based in the country or countries of study had during a) study design; b) clinical study processes, such as processing blood samples, prescribing medication, or patient recruitment; c) data interpretation; and d) manuscript preparation, commenting on all aspects. If they were not involved in any of these aspects, please explain why.

   This should include a thorough description of their leadership roles in the study. Are local researchers named in the author list or the acknowledgements, or are they not mentioned at all (and, if not, why)? Please also describe the involvement of early career researchers based in the location of the study. Some of this information might be repeated from the Contributors section in the manuscript. Note: we adhere to ICMJE authorship criteria for naming authors on a paper.

**a) Study design:**<br>Prof Mosepele Mosepele<br>Prof Nthabiseng Phaladze<br>Dr Tendani Gaolathe<br>Mr Kago Kebotsamang
**b) Clinical study processes:**<br>Prof Mosepele Mosepele<br>Prof Nthabiseng Phaladze<br>Dr Tendani Gaolathe<br>Dr Mareko Ramotsababa<br>Dr Ponego Ponatshego<br>Dr Onkabetse Julia Molefe-Baikai<br>Dr Thato Moshomo<br>Dr Kesaobaka Molebatsi<br>Mr Kago Kebotsamang<br>Mr Moagedi Mawi
**c) Data interpretation:**<br>Prof Mosepele Mosepele<br>Prof Nthabiseng Phaladze<br>Dr Tendani Gaolathe<br>Dr Onkabetse Julia Molefe-Baikai<br>Dr Thato Moshomo<br>Dr Kesaobaka Molebatsi<br>Mr Kago Kebotsamang<br>Mr Moagedi Mawi
**d) Manuscript preparation:**<br>Prof Mosepele Mosepele<br>Prof Nthabiseng Phaladze<br>Dr Tendani Gaolathe

Dr Onkabetse Julia Molefe-Baikai
Dr Thato Moshomo
Dr Kesaobaka Molebatsi
Mr Kago Kebotsamang
Mr Moagedi Mawi

2. How was funding used to remunerate and enhance the skills of researchers in the countries of study? And how was funding used to improve research infrastructure at the study sites?

Potentially effective investments into long-term skills and opportunities within local institutions could include training or mentorship in analytical techniques and manuscript writing, opportunities to lead all or specific aspects of the study, financial remuneration rather than requiring volunteers, and other professional development and educational opportunities.

Improvements to research infrastructure could include funding extended trial designs (eg, platform trials), establishment of long-term contracts for research staff, building research facilities, and setting up local control of funding allocation.

Skills:
The following junior members of the team were mentored on implementing a clinical trial, data management
Dr Onkabetse Julia Molefe-Baikai
Dr Thato Moshomo
Dr Kesaobaka Molebatsi
Mr Kago Kebotsamang
Mr Moagedi Mawi

The following junior mentees proposed nested sub-studies in InterCARE
Dr Mareko Ramotsababa
Dr Ponego Ponatshego
Dr Onkabetse Julia Molefe-Baikai
Dr Thato Moshomo
Dr Kesaobaka Molebatsi
Mr Kago Kebotsamang
Mr Moagedi Mawi

The following mentees received external research funding for nested sub-studies within InterCARE
Dr Mareko Ramotsababa
Dr Ponego Ponatshego
Dr Onkabetse Julia Molefe-Baikai
Dr Matlhogonolo Kelepile

The following mentees led manuscripts (as first Authors) on some baseline studies using InterCARE data
Dr Onkabetse Julia Molefe-Baikai
Dr Thato Moshomo
Mr Moagedi Mawi
Dr Matlhogonolo Kelepile

**Research infrastructure:**<br><br>InterCARE data set will be used for mentorship of local junior researchers and post-doctoral students at the University of Botswana<br><br>InterCARE set up is serving as the basis for new active ongoing new clinical trials

3. How did you safeguard the researchers who implemented the study?

   Please describe how you guaranteed safe working conditions for study staff, including provision of appropriate personal protective equipment, protection from violence, and prevention of overworking.

> Consistent with University of Botswana and Botswana Labour laws
> - Employees often responded to public adverts (fair opportunity to get employees)
> - Those short listed for work interview would have a panel of 3 or more people interviewing applicants. All applicants for the same job would be asked the same question(s), and scored using the same scoring sheet
> - Those offered employment, received this offer from office of Human Resource Manager (Faculty of Medicine)
> - Offer of employment included a contract (consistent with Botswana labor law provision and protections)- this contract states working hours, renumerations, etc
> - Where required (e.g., data, cell phones, tablets), were provided

<u>*Benefits to the communities and regions of study*</u>

4. How does the study address the research and policy priorities of its location?

   How were the local priorities determined and then used to inform the research question? Who decided which priorities to take forward? Which elements of the study address those priorities?

> The basis InterCARE is the Botswana Multisectoral NCD strategy- which stipulated that 70% or more people living with hypertension in Botswana should achieve blood pressure control. So, InterCARE was intended to develop strategies help people living with hypertension to achieve the stated national target.

5. How will research products be shared in the community of study?

   For instance, will you be providing written or oral layperson summaries for non-academic information sharing? Will study data be made available to institutions in the region(s) of study? The Lancet Group *encourages authors to translate the summary (abstract) into relevant languages after paper editing; do you intend to translate your summary?*

There are several plans for dissemination:

- Flyers is simple and easy to understand language
- In person meeting with community members
- In person meetings with Ministry of Health and Ministry of Local government officials
- Radio and TV interviews

6. How were individuals, communities, and environments protected from harm?

a) *How did you ensure that sensitive patient data were handled safely and respectfully? Was there any potential for stigma or discrimination against participants arising from any of the procedures or outcomes of the study?*

All study staff completed "Good Clinical Practice" (GCP) certification
Data was kept on RedCap© and there were restriction [required pass word to access data, and study team had different levels of access based on role]

b) *Might any of the tests be experienced as invasive or culturally insensitive?*

N/A

c) *How did you determine that work was sensitive to traditions, restrictions, and considerations of all cultural and religious groups in the study population?*

Community members and their healthcare providers provided feedback on our implementation strategies, that way, we made sure our work was sensitive to traditions, restrictions and considerations of the community

d) *Were biowaste and radioactive waste disposed of in accordance with local laws?*

N/A

e) *Were any structures built that would have impacted members of the community or the environment (such as handwashing facilities in a public space)? If so, how did you ensure that you had appropriate community buy-in?*

N/A

f) *How might the study have impacted existing health-care resources (such as staff workloads, use of equipment that is typically employed elsewhere, or reallocation of public funds)?*

The study promoted integrated care to reduce work load for staff. No new resources were required. No reallocation of funds was required.

7. Confirm that local ethics review was sought, and please provide the approval number. If not sought, please explain why.

Local Ethics Approval was granted. Permit number is HPRD6/14/1

Secondary analyses

8. Have the data analysed in your study been extracted from another source, such as a national survey, rather than being directly collected by the authors of this paper?

N/A

If the authors of this paper were not involved in data collection, how were the findings interpreted with sufficient contextual knowledge?
The Lancet Group *believe contextual understanding is crucial for informed data analysis and interpretation.*

N/A. See above. Several local authors were involved in data collection

---

9. Please provide the title (eg, Dr/Prof, Mr/Mrs/Ms/Mx), name, and email address of an author who can be contacted about this statement.

Name: Prof Mosepele Mosepele
Email: mosepelemosepele@gmail.com

10. Finally, please provide the title and name of an author from one country of study who has seen and approved this form.

Name: Dr Thato Moshomo
